# Creating Novel Activated Factor XI Inhibitors through Fragment Based Lead Generation and Structure Aided Drug Design

**DOI:** 10.1371/journal.pone.0113705

**Published:** 2015-01-28

**Authors:** Ola Fjellström, Sibel Akkaya, Hans-Georg Beisel, Per-Olof Eriksson, Karl Erixon, David Gustafsson, Ulrik Jurva, Daiwu Kang, David Karis, Wolfgang Knecht, Viveca Nerme, Ingemar Nilsson, Thomas Olsson, Alma Redzic, Robert Roth, Jenny Sandmark, Anna Tigerström, Linda Öster

**Affiliations:** 1 Medicinal Chemistry, Cardiovascular & Metabolic Diseases Innovative Medicines, AstraZeneca R&D, Mölndal, Sweden; 2 Discovery Sciences, AstraZeneca R&D, Mölndal, Sweden; 3 Bioscience, Cardiovascular & Metabolic Diseases Innovative Medicines, AstraZeneca R&D, Mölndal, Sweden; 4 Drug Metabolism and Pharmacokinetics, Cardiovascular & Metabolic Diseases Innovative Medicines, AstraZeneca R&D, Mölndal, Sweden; University of Parma, ITALY

## Abstract

Activated factor XI (FXIa) inhibitors are anticipated to combine anticoagulant and profibrinolytic effects with a low bleeding risk. This motivated a structure aided fragment based lead generation campaign to create novel FXIa inhibitor leads. A virtual screen, based on docking experiments, was performed to generate a FXIa targeted fragment library for an NMR screen that resulted in the identification of fragments binding in the FXIa S1 binding pocket. The neutral 6-chloro-3,4-dihydro-1H-quinolin-2-one and the weakly basic quinolin-2-amine structures are novel FXIa P1 fragments. The expansion of these fragments towards the FXIa prime side binding sites was aided by solving the X-ray structures of reported FXIa inhibitors that we found to bind in the S1-S1’-S2’ FXIa binding pockets. Combining the X-ray structure information from the identified S1 binding 6-chloro-3,4-dihydro-1H-quinolin-2-one fragment and the S1-S1’-S2’ binding reference compounds enabled structure guided linking and expansion work to achieve one of the most potent and selective FXIa inhibitors reported to date, compound 13, with a FXIa IC_50_ of 1.0 nM. The hydrophilicity and large polar surface area of the potent S1-S1’-S2’ binding FXIa inhibitors compromised permeability. Initial work to expand the 6-chloro-3,4-dihydro-1H-quinolin-2-one fragment towards the prime side to yield molecules with less hydrophilicity shows promise to afford potent, selective and orally bioavailable compounds.

## Introduction

A well balanced haemostasis system is important to both minimize blood loss and disturbances of blood flow. Upon injury of the vessel wall, blood is exposed to tissue factor which via a cascade reaction leads to thrombin generation and a fibrin cross-linked clot to mend the injury and stop bleeding. Factor XI (FXI) has an important role in thrombin generation in the amplification phase of the coagulation process. However, over-production of thrombin may lead to excessive clots resulting in thrombosis. Also, high levels of thrombin cause activation of thrombin activated fibrinolysis inhibitor which hinders fibrinolysis. Therefore, decreased levels of thrombin will indirectly increase the rate of fibrinolysis. Inhibition of activated FXI (FXIa) should decrease thrombin generation in the amplification phase, but not in the initiation phase, and thus yield an antithrombotic and profibrinolytic effect with minimal risk of bleeding (see reviews [1–3]). Bleeding is a serious concern with current antithrombotic drugs and FXIa inhibitors could address this issue.

The role of FXIa in haemostasis and thrombosis in human has been extensively studied. Human haemophilia C patients who are severely deficient in FXI display reduced incidence of ischemic stroke [4]. Unlike haemophilia A and B patients, who are deficient in FVIII and FIX, respectively, haemophilia C patients seldom experience spontaneous bleeding [5]. The bleeding associated with FXI deficiency usually occurs after trauma or surgery in the tissues with high fibrinolytic activity [6,7]. An increased level of factor XI has been reported as a risk factor for deep venous thrombosis [8,9], myocardial infarction [10] and ischemic stroke [11,12]. There is also much research on the role of FXI in animals. Several studies have demonstrated that FXI-null mice are protected against venous and arterial thrombosis without an adverse effect on bleeding time [13–18]. Recent reports present similar effects in mice [19] and primates [20] using antisense oligonucleotides to inhibit FXI production [19]. Antibodies against FXI/FXIa have been shown in one study to reduce thrombus growth in the rabbit iliac artery in the presence of repeated balloon injury [21], and in another study to increase endogenous thrombolysis in rabbit about two-fold in comparison to control antibodies [22]. Also, an anti-human antibody, aXIMab, prevented vascular graft occlusion in baboons [23]. In summary, there is ample evidence in support of FXIa as an attractive antithrombotic and profibrinolytic target.

FXIa small molecule inhibitors have not reached the same level of maturity as thrombin and activated factor X (FXa) inhibitors. The thrombin inhibitor dabigatran [24] and the FXa inhibitor rivaroxaban and apixaban [25] are approved anticoagulant drugs in several markets, but adverse bleeding remains an area where improvement is requested. In contrast, inhibitors of FXIa are still in preclinical development. Daiichi Sankyo Co has reported on potent and selective peptidomimetic alpha-ketothiazole arginine based covalent FXIa inhibitors [26,27], and one compound was shown to display similar antithrombotic efficacy as heparin in a rat venous thrombosis model [26]. Similarly, Bristol Myers Squibb (BMS) demonstrated *in vivo* antithrombotic efficacy in rat models with BMS-262084, a potent and selective beta-lactam arginine that irreversibly inhibits FXIa with an IC_50_ of 2.8 nM [28]. Recently, BMS also showed antithrombotic efficacy without increased bleeding in a rabbit model with a reversible selective small molecule FXIa inhibitor [29]. Patent applications from BMS display lists of selective FXIa inhibitors, or dual FXIa and plasma kallikrein inhibitors, with IC_50_ values in the low nM range [30–32]. These examples encourage further work with the aim of reaching the clinical setting for small molecule FXIa inhibitors.

In-house high throughput screening (HTS) attempts had previously failed to identify viable leads. Therefore, structure aided fragment based lead generation (FBLG) was chosen as a rescue strategy to create new FXIa inhibitor leads. The choice was supported by the feasibility to generate X-ray structures, to perform a fragment screen using nuclear magnetic resonance (NMR) and to apply structure based virtual screening to select a fragment library.

A typical fragment campaign involves screening of a relatively small set (< 5000) of small molecules of low structural complexity against a biological target. Binding to the target is identified using a robust assay, usually a biophysical technique like NMR [33–35], surface plasmon resonance (SPR) [36–40], mass spectroscopy (MS) [41] or X-ray crystallography [42–44]. These techniques are suitable in fragment campaigns, as they allow for detection of the very weak binding (dissociation binding constant (*K*_D_) in the mM range) that can be expected for the small molecules screened. Fragment based approaches to drug discovery have during the last years presented many successful examples [45,46], and at least one campaign has lead to a product on the market [46,47].

**Fig 1:**
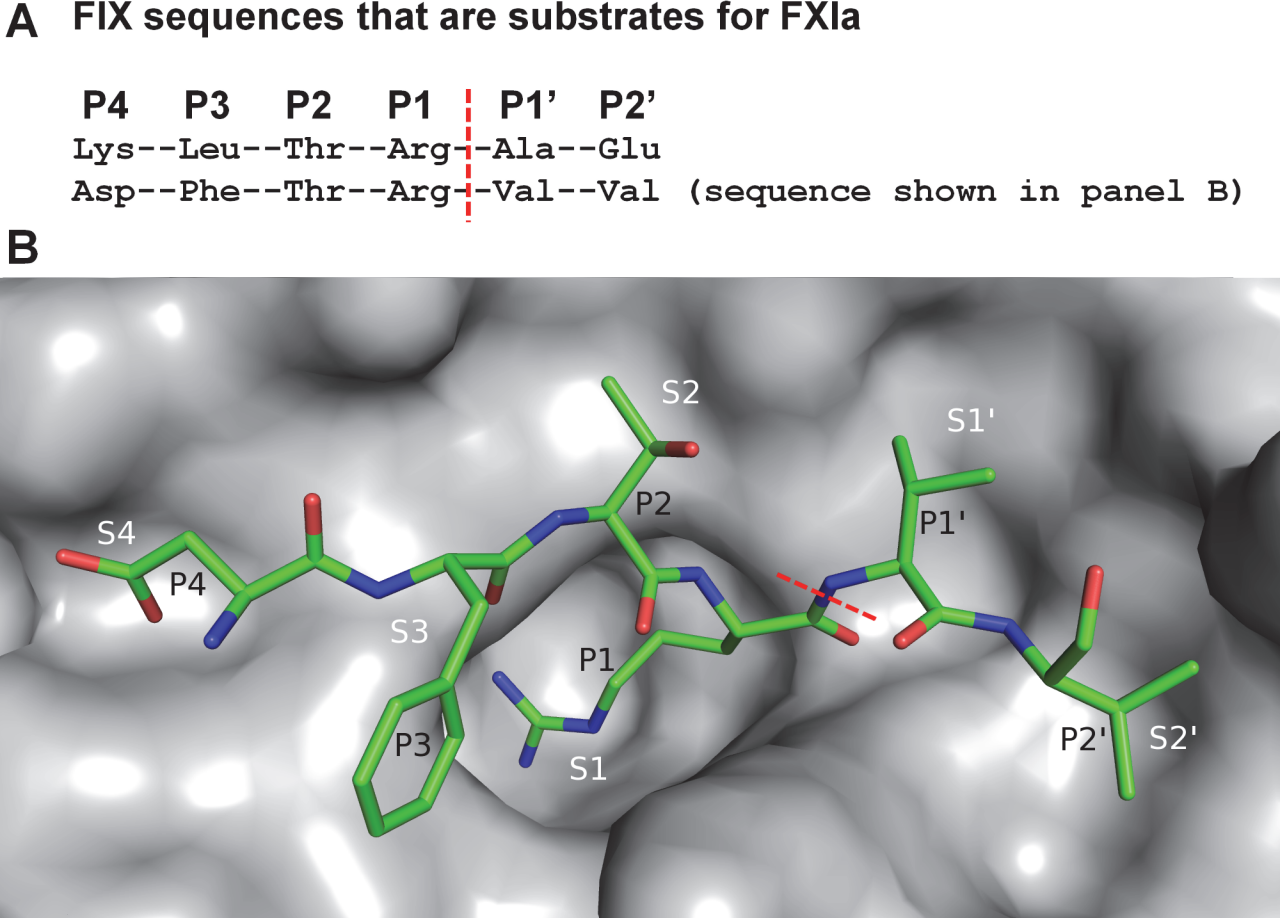
Nomenclature for FXIa substrates and corresponding binding sites. (A) FIX sequences that are substrates for FXIa. The scissile bonds cleaved by FXIa are marked with a red dashed line. Residues N- and C-terminal of the scissile bond are referred to as P1, P2 etc. and P1’, P2’ etc., respectively. (B) Depiction of FXIa active site in complex with FIXa substrate residues (from PDB entry 1XXD [82]). According to standard nomenclature, the substrate P1 residue binds the enzyme S1 site, the P1’ residue binds the S1’ site, and so on. The scissile bond is marked with a red dashed line.

Fig. 1 depicts the FXIa active site and describes the standard nomenclature according to Schechter and Berger [48] for substrate and ligand binding to this site. P1 and P1’ denote the peptide residues N- and C-terminal of the scissile bond, and S1 and S1’ denote the corresponding enzyme binding sites.

The aim of the present study was to create novel FXIa inhibitors with potential to be developed into potent, selective and orally bioavailable compounds by targeting the S1-S1’-S2’ FXIa binding pockets. In the FXIa FBLG campaign presented here, virtual screening, NMR, SPR and X-ray crystallography together with enzymatic inhibition assays were successfully applied. We report the identification of two novel FXIa S1 binding fragments and the evolution of these into nM inhibitors using X-ray structures to guide the design. Initial fragment expansion work shows promise to afford potent, selective and orally bioavailable compounds, which motivates further optimization work on presented compounds.

## Results and Discussion

### Fragment library selection

The structural characteristics of the target protein binding site and the quality of the initial library of fragments to be screened are two critical determinants for the success of an FBLG campaign. The most prominent and probable fragment binding site of FXIa is the S1 pocket. A key feature of the S1 pocket is Asp189 (trypsin numbering) deep down in the cavity and most known ligands contain a strongly basic group that forms ionic interactions with this residue [26,27,49]. However, it has been shown that the basicity is not required [50,51], and since strongly basic P1 groups often pose high hurdles in achieving adequate oral bioavailability, we steered our fragment selection towards non-basic or weakly basic compounds. Importantly, the S1 pocket in FXIa harbors an alanine in position 190, a feature shared with thrombin and FXa whereas the corresponding residue in activated factor IX (FIXa) and activated factor VIIa is serine. The Ala190 provides a pocket where a neutral P1 is likely to be more easily accommodated than when this residue is a serine. Apart from S1, cavity analyses using the Schrödinger Sitemap tool [52] suggested that favorable ligand-protein interactions could be picked up towards the S1’-S2’ sites in FXIa. Also, comparisons of primary sequences and structural features around the S1’-S2’ sites for thrombin, FXa and FXIa suggested opportunities for achieving selective FXIa inhibitors. Thus, the project set out to primarily target the S1-S1’-S2’ binding pockets.

**Fig 2:**
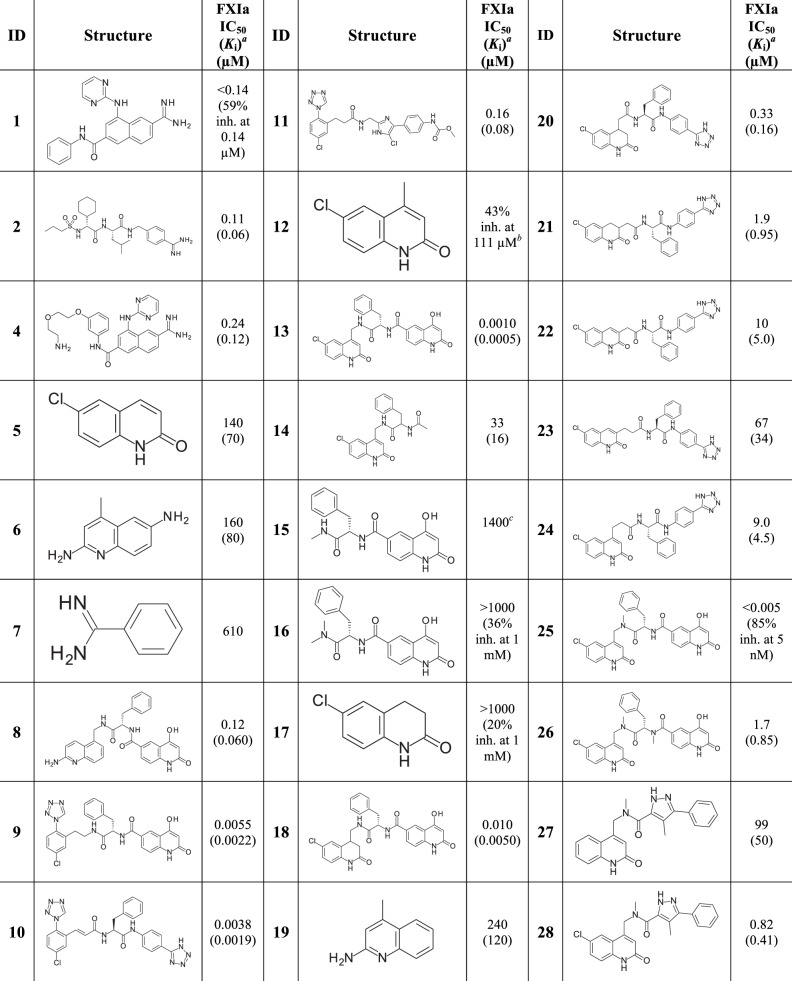
Structures with associated FXIa potencies. ^*a*^ *K*_i_ values are shown in parenthesis. ^*b*^Precipitates at higher concentration than 111 μM. ^*c*^ *K*_D_ value from Biacore binding experiment.

The initial fragment selection focused on targeting the S1 site. A total of about 65000 fragments from the AstraZeneca screening collection with MW < 250 g/mol were docked using the Glide software from Schrödinger [53–55] to the FXIa X-ray structure (PDB id 1ZSJ), with the N-(7-Carbamimidoyl-naphthalen-1-yl)-3-hydroxy-2-methyl-benzamide ligand and all water molecules removed. Docked fragments with a halogen and/or nitrogen interacting with S1 pocket residues were then manually inspected for a selection of about 1200 structures. The same 65000 fragments were also docked into an in-house structure of FXIa catalytic domain (CD) in complex with compound **1**, see Fig. 2, with the ligand and waters removed. From these docking experiments, an additional 600 structures were selected based on Glide score and chemical tractability. In total, 1800 structures were submitted to experimental screening.

### Screening cascade

Ligand detected one dimensional (1D) NMR spectroscopy [56–58] was used as the primary fragment screening tool, followed by SPR and enzymatic assays to determine *K*_D_ and IC_50_ values, respectively. Finally, structures of the FXIa catalytic domain, containing the four mutations described by Jin et al., [59] (herein referred to as FXIa CD) in complex with ligands were solved by X-ray crystallography.

**Fig 3:**
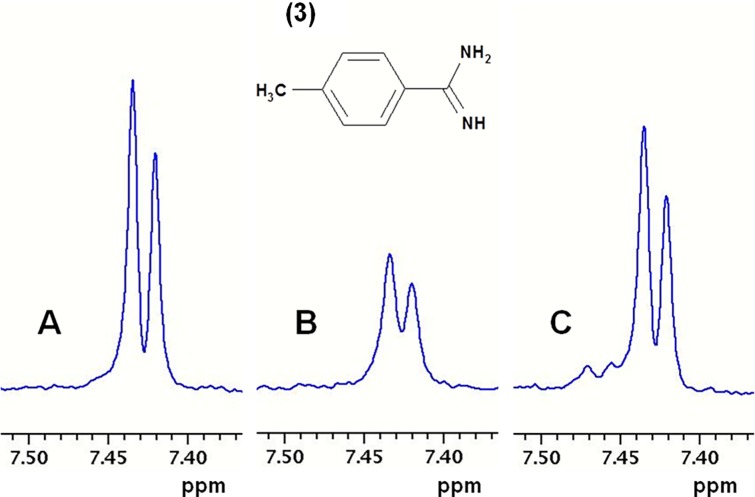
Ligand detected one dimensional NMR spectroscopy. Part of ^1^H T1rho NMR spectra of p-methyl-benzamidine (pMeBza): (A) 100 μM pMeBza in buffer; (B) after addition of 5 μM FXIa CD; and (C) after addition of 10 μM compound 2. Shown is the doublet from the protons in the ortho position. The extra signals in C are from the inhibitor.

**Ligand detected 1D NMR**. Compounds were screened at 100 μM in mixtures of six. Weakly binding fragments were identified from intensity changes through measurements on three samples *viz*. (1) mixtures of fragments at 100 μM in buffer; (2) with addition of 5 μM FXIa CD; (3) with subsequent addition of 10 μM of the high affinity inhibitor compound **2** (see Fig. 2). The signal from a compound binding with low affinity to FXIa CD decreased upon addition of protein and regained upon addition of a high affinity reference compound. Comparison with reference spectra of individual compounds enabled identification of the binding fragment in a mixture. Compound **2** binds to the S1-S2-S3-S4 pockets of FXIa CD with an IC_50_ of 110 nM, and is expected to displace any fragment binding weakly to the S1 site as well as to the non-prime side of the binding pocket, i.e. S2-S3-S4. As a positive control, the S1 binding ligand para-methyl-benzamidine (pMeBza), compound **3**, was used, and in Fig. 3 the doublet at 7.66 ppm is shown.

**Fig 4:**
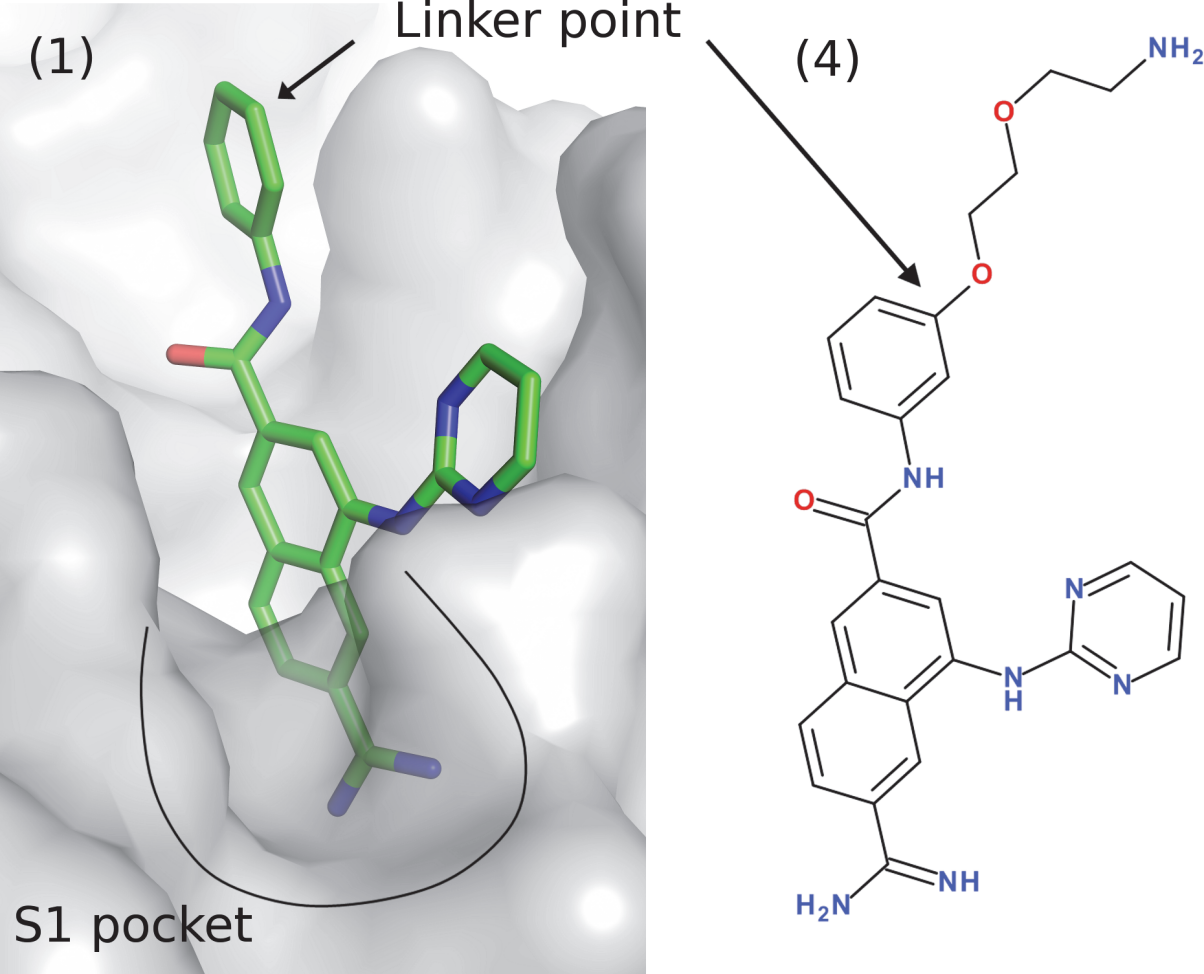
Crystal structure of compound 1 in complex with FXIa. The surface exposed point for linker attachment used to develop compound 4 is highlighted. The *K*_D_ of the compound 4 was determined to 390 nM. Compound 4 was linked to the sensor chip *via* the primary amine.

**SPR**. Compounds that displayed binding to FXIa CD in the NMR screen were followed up by *K*_D_ determinations using SPR. The SPR data were then used to rank and select compounds for X-ray crystallography experiments. The SPR method was based on an inhibition in solution assay in which compound **4**, immobilized on the biosensor surface, served as a probe for the FXIa catalytic site. The advantages of inhibition in solution assay as compared to the more conventional direct binding assays where the protein is immobilized include (1) substantially increased sensitivity, (2) generic and rapid assay development and (3) immediate verification of competitive binding. Fig. 4 illustrates the design of compound **4** based on the crystal structure of FXIa in complex with compound **1**. Since any ligand that binds to the S1 pocket competes with compound **4** for binding to FXIa CD, it is possible to derive the *K*_D_ for that ligand [60]. The initial binding rate of the protein to compound **4,** *K*_on_, was used to determine the percentage of free protein in solution which changed by varying the concentration of the competing ligand. The free protein concentration was plotted against the logarithm of the ligand concentration and a sigmoidal dose–response curve-fit model was applied to determine the *K*_D_-value.

**Enzymatic assay**. All fragments that showed competitive binding in the NMR assay (fragment hit compounds) and all compounds resulting from expansions of fragments were screened in an enzymatic assay to determine their inhibitory activity against FXIa. Selected compounds were also screened against other serine proteases to evaluate their selectivity profiles. All protease activities were determined by chromogenic assays monitoring the release of para-nitroanilide at 405 nm.

**X-ray crystallography**. Selected fragments and expanded compounds that had been confirmed to be binding to the active site by either NMR or SPR were attempted for structure determination by X-ray crystallography. To improve crystallisability, the quadruple mutant (Ser75Ala, Lys78Ala, Thr97Ala and Cys123Ser), first reported by Jin et al., [59] was generated. The mutated residues in this construct (FXIa CD) are located at least 16 Å from the catalytic residue Ser195 and the alterations are therefore not anticipated to impact ligand binding in the active site. The selected fragments were either co-crystallized with FXIa CD, or the protein crystals were soaked in a solution containing the ligand. The crystals typically diffracted to around 2 Å on an in-house X-ray source. Fragments were considered for chemical expansion if their binding mode could be determined by X-ray crystallography.

### Screening results

**Fig 5:**
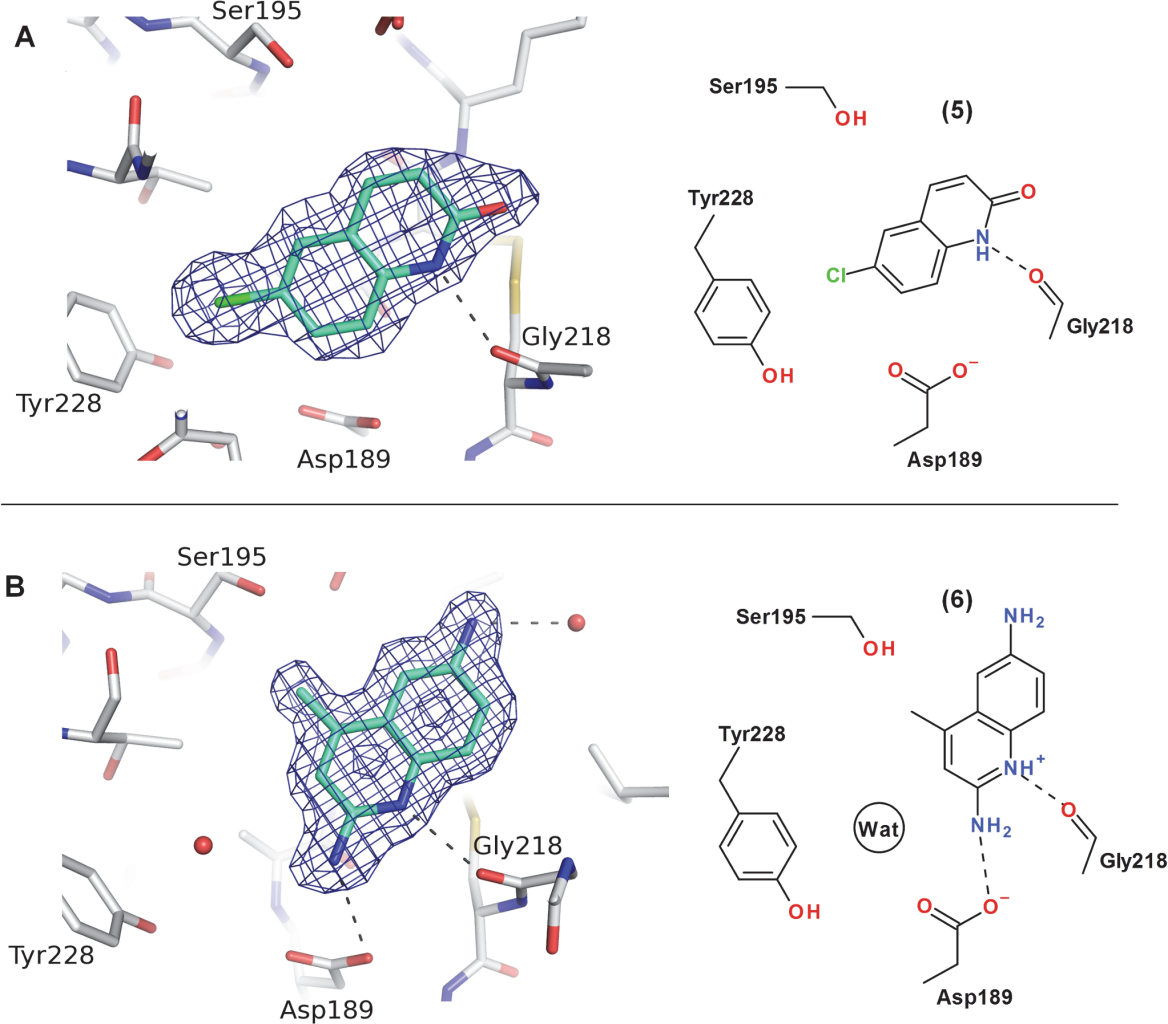
Crystal structures of compounds 5 (A) and 6 (B) in complex with FXIa. Hydrogen bonds are depicted as dashed lines and the refined 2F_o_F_c_ electron density maps are contoured at 1σ. Compound 6 is depicted in the charged form to satisfy the hydrogen bond to Gly218.

NMR screening of the 1800 structures selected by virtual screening resulted in 13 neutral or weakly basic fragment hits (calculated pKa < 9.0, Advanced Chemistry Development, Inc. ACD/LabspKaDB (version 6.0) software [61]). These hits were FXIa S1 binding fragments as demonstrated by pMeBza competition in NMR experiments. About 600 near neighbours to the original 13 hits were selected and screened by NMR to yield a total of 50 hits. Several of the fragment hit compounds were only weakly basic, such as aminopyridines and aminoquinolines, and neutral compounds, such as chloroquinolinones and the known p-chlorophenyltetrazoles [32], were also identified. Two prioritized fragment hits, 6-chloro-3,4-dihydro-1H-quinolin-2-one (**5**) and 4-methylquinolin-6-amine (**6**) are shown in Fig. 5. To our knowledge, **5** is a novel serine protease P1 structure, and **6** is here described for the first time to bind FXIa. The crystal structures of compounds **5** and **6** in complex with FXIa were solved to 2.0 and 1.7 Å resolution, respectively, and the fragments could be unambiguously placed in the electron density maps (Fig. 5). The two fragments display distinct binding modes as depicted in Fig. 5. These binding modes were predicted from the GLIDE dockings of compounds **12** and **19** in the virtual screen. After alignment of the proteins using the SSM function in Coot [62], a root mean square deviation (r.m.s.d.) of 0.44 Å for the compound **12** docking pose and crystal structure was calculated using heavy atom positions only. For compound **6**, the binding mode was predicted using the docking pose of compound **19** (r.m.s.d. of 0.61 Å including all heavy atoms except for the amino group in the 6-position).

Fragment **5** displayed a FXIa IC_50_ of 140 μM. The chloro atom is directed almost perpendicular to the aromatic ring of Tyr228 and is located at a distance of 3.8 Å to the closest carbon atom of the phenyl ring. This chloro atom replaces a highly conserved water molecule (see Fig. 5B) and its position is similar to the chloro atom in chloro-phenyl containing P1s of FXIa, thrombin and FXa inhibitor [63,64]. The halogen bonding between electron rich groups including pi systems and the σ-hole of the halogen with a clear directionality has recently received attention and has been reviewed in several articles [65–68]. The chloroquinolinone compound **5** picks up an additional interaction with FXIa, *i*.*e*. the quinolinone NH group forms a 2.7 Å hydrogen bond with the backbone carbonyl of Gly218. Notably, the chloroquinolinone is slightly more potent than benzamidine, compound **7** (see Fig. 2), despite the fact that the former is neutral and does not form polar interactions with Asp189. This suggests that the chloroquinolinone P1 is a promising FXIa fragment inhibitor, with potential to be expanded and developed into an orally active drug.

The aminoquinoline fragment **6** shown in Fig. 5 binds to the S1 pocket with an IC_50_ of 160 μM. The calculated pKa 7.9 (ACD/LabspKaDB [61]), is supported by an experimental pKa value of 7.1 from the expanded analogue, compound **8**. The amino group of the ligand interacts with the carboxyl group in Asp189 through a 3.1 Å hydrogen bond, and the quinoline nitrogen forms a 2.9 Å hydrogen bond with the carbonyl group of Gly218 (Fig. 5). Both the quinolinone and the aminoquinoline fragments form hydrogen bonds to Gly218, even though their overall binding geometries in the S1 pocket are quite different. The conserved water molecule stacking against Tyr228 is bound in the S1 pocket close to the fragment with a distance of 3.6 Å between the ligand amine nitrogen and the water oxygen.

### Structure aided fragment expansion

**Fig 6:**
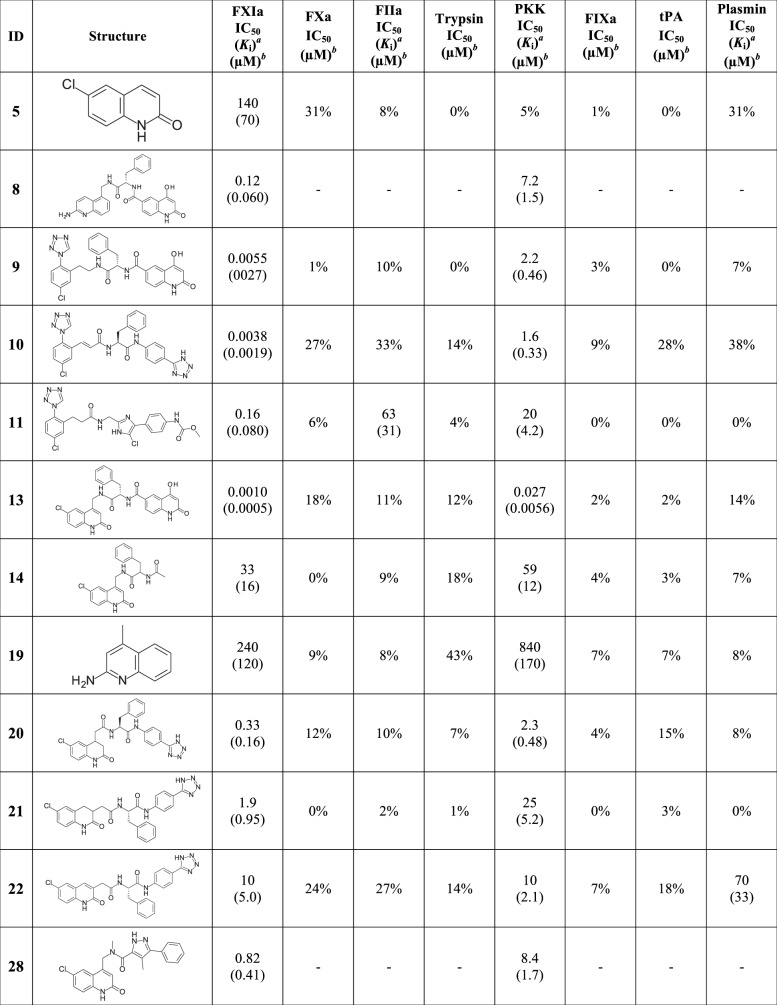
Structures with associated selectivity data. ^*a*^ *K*_i_ values are shown in parenthesis. ^*b*^% inhibition of the compounds at 99 μM are given if the IC_50_ was above 99 μM.

**Fig 7:**
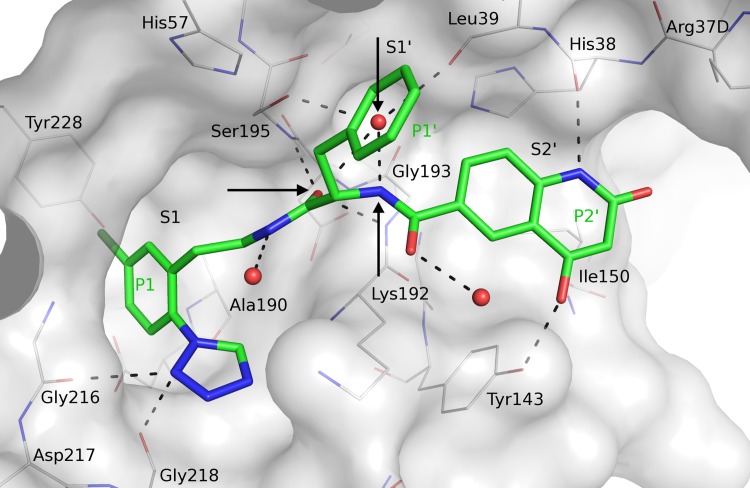
Crystal structure of compound 9 [31] in complex with FXIa. Compound 9 is displayed in thick sticks with green carbons, selected binding site residues in thin sticks with grey carbons, water oxygens in red spheres, and hydrogen bond interactions with dashed lines. The arrows indicate: (1) the central water molecule, (2) the hydrogen acceptor of the P1-P1’ amide and (3) the hydrogen donor of the P1’-P2’ amide.

In parallel with the fragment hit identification work, compounds **9**–**11** from patent applications from Bristol Myers Squibb [30,31] were synthesized and characterized. The compounds are potent FXIa and plasma prekallikrein inhibitors and selective against *e*.*g*. thrombin and FXa as seen in Fig. 6. The crystal structures of **9**–**11** in complex with FXIa CD were not available in the public domain and were therefore solved by X-ray crystallography. Given our aim to target the prime side, it was exciting to note the S1-S1’-S2’ binding mode for all three compounds, as shown for compound **9** in Fig. 7. Superposition of the crystal structures showed that the chloro-phenyl group of compound **9** overlays with the corresponding group of the chloroquinolinone fragment (compound **5**), and the chloro-phenyltetrazole group binds as anticipated from previously reported thrombin structures in complex with ligands carrying the same P1 [63,64]. The P1-P1’ amide group, extended from the P1 via an ethyl linker, interacts with the active site Ser195 where the carbonyl oxygen forms hydrogen bonds with the amide nitrogen of Ser195 and a central tightly bound water molecule (Fig. 7). The benzyl group acts as P1’ and binds in the S1’ cavity and the amine of the amide group linking P1’ and P2’ forms a hydrogen bond with the central water molecule that is not only located within hydrogen bond distances to both amide groups of the inhibitor, but also to the hydroxyl of Ser195 and to the backbone carbonyl of Leu39. The carbonyl from the P1-P1’ amide is positioned in the oxyanion hole with hydrogen bonds to the main chain nitrogens of Gly193 and Ser195. The P2’ hydroxyquinolinone moiety interacts with His38 and Tyr143 in the S2’ pocket. Two glycerol molecules, originating from the cryo solution from the crystal freezing step, were bound in close vicinity to the ligand, one stacking against the hydroxyquinolinone in the S2’ region and the second was bound close to the benzyl moiety (not shown). However, since the S2’ substituent of compound **11** shows a highly similar pose (not shown), the glycerol molecules do not appear to alter the binding mode. It should be noted that after completion of the experimental work presented here, the structures of two FXIa inhibitors with closely analogous P1’-P2’ moieties that show similar binding modes have been published [51].

**Fig 8:**
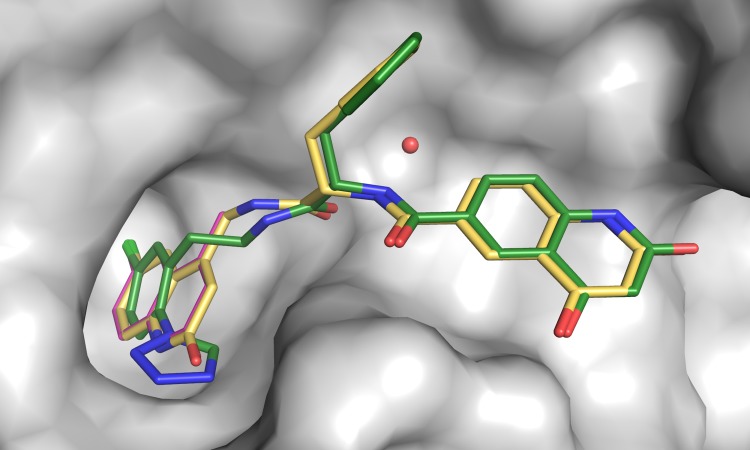
Crystal structures of compounds 9, 12 and 13 in complex with FXIa. Compounds 9 (green), 12 (magenta) and compound 13 (yellow) are overlaid. The protein surface from the FXIa CD:compound 9 complex is shown as grey surface and the central water molecule that interacts with both amides is shown as sphere.

The crystal structures of compounds **9**–**11** in complex with FXIa CD gave a clear handle on directed expansion of fragments towards the prime side and allowed linking exercises. Fragment **5** was selected as the primary target for linking/expansion work. Disregarding overall physicochemical properties in general and ADME properties in particular, the first key question addressed was if the novel chloroquinolinone P1 could be linked to the P1’-P2’ parts of compound **9** to yield highly potent and selective FXIa inhibitors. Fig. 8 outlines the structural basis and outcome of this exercise. The X-ray structures of the FXIa CD complexes with the chloroquinolinone methylated in the 4-position (compound **12**) and compound **9** were aligned in order to guide linking. Based on X-ray structure information and docking studies, we deduced that the hydrogen bond acceptor in the defined space as exemplified by the P1-P1’ amide carbonyl oxygen in compound **9** (see Fig. 7) was favorable and thus judged critical to maintain in any designed new compounds. Another key feature is a hydrogen donor moiety, exemplified by the P1’-P2’ amide nitrogen that interacts with a central water molecule that is highly conserved in position (see Fig. 7). Thus, linkers were designed to preserve the experimentally determined positions of both the P1 fragment, the P1-P1’ amide carbonyl oxygen and the P1’-P2’ amide nitrogen, without introducing steric strain. A range of possible linker sizes and geometries were conceived by visual inspection of the overlay of available protein-ligand crystal structures, and subsequently tested and prioritized by docking experiments of linked full length compounds using GOLD [69] and GLIDE [53–55]. The most promising compounds with unstrained geometry, maintaining the linked moieties in place, were chosen for synthesis. No estimate for the free energy of binding was used, but the approach rather selected compounds for synthesis based on good predicted geometry, leaving the actual binding affinity to be determined experimentally. The first linker chosen to connect compound **12** and the P1’-P2’ parts of compound **9** resulted in compound **13**, which binds FXIa CD with a *K*_D_ of 0.5 nM and is one of the most potent FXIa inhibitors (IC_50_ = 1.0 nM) reported to date.

**Fig 9:**
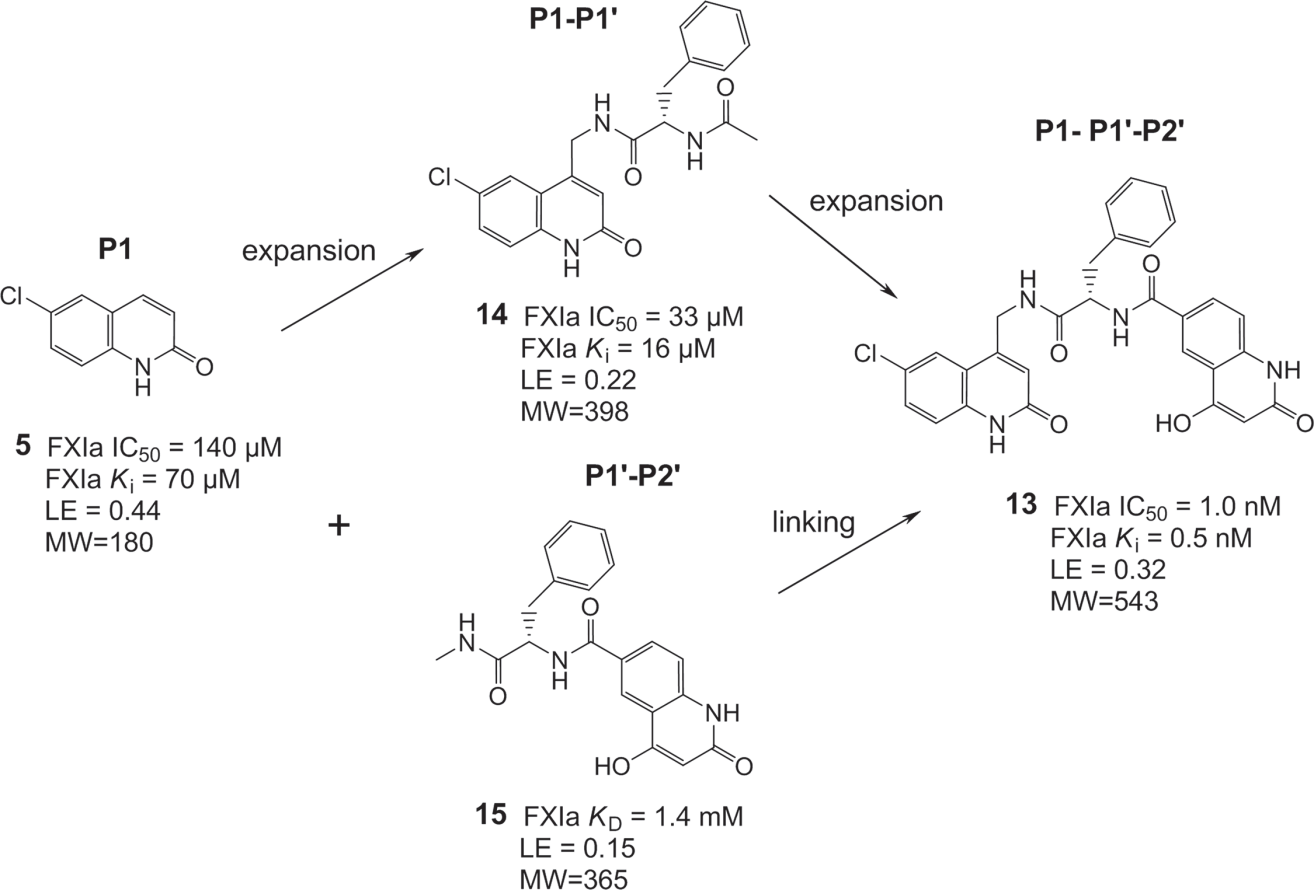
Illustration of potency SAR for initial expansion/linking.

The potency SAR observed in this first expansion/linking work is summarized in Fig. 9. The FXIa IC_50_ of the chloroquinolinone **5** P1 binder is 140 μM with a ligand efficiency [70] (LE) of 0.44. The P1-P1’ compound **14** increasing in MW from 180 to 398 resulted in only minor improvement in potency with an IC_50_ of 33 μM and a drop in LE to 0.22. Adding on the P2’ part boosted the potency more than four orders of magnitude to an IC_50_ of 1 nM and a LE of 0.32 (compound **13**). The isolated P1’-P2’ moiety, compound **15**, showed a poor binding affinity (*K*_D_ = 1.4 mM), and the N,N-dimethylated analogue, compound **16**, confirmed S1’-S2' binding with a nearly perfect overlap with the identical P1’-P2’ groups of compound **13** (X-ray data not shown). The SAR outlined above can be regarded as an example of superadditivity energetics achieved by fragment linking, similarly as recently described in detail by Nazaré et al. for FXa inhibitors [71]. Rigid body entropy loss is expected to be similar for the individual fragments (here, fragments **5** and **15**) as for the final fragments linked molecule (here, compound **13**). This rigid body entropy loss has been estimated as 15–20 kJ/mol at 298 K by Murray and Verdonk [72]. Thus, superadditivity with respect to the starting fragments binding affinities, in the range of three orders of magnitude, is expected when fragments are optimally linked. Adapting the following equation from Nazaré et al. [71],

$$\Delta {\text{G}}_{\text{link}}=\Delta {\text{G}}_{\text{final}}-\Delta {\text{G}}_{\text{frag1}}-\Delta {\text{G}}_{\text{frag2}}$$

to the present example yields:

Δ*G*_link_ = Δ*G*_compound **13**_ - Δ*G*_fragment **5**_ - Δ*G*_fragment **15**_ = -53.1 kJ/mol + 23.7 kJ/mol + 16.3 kJ/mol = -13.1 kJ/mol. This linker contribution of -13.1 kJ/mol corresponds to an improvement by about 2.3 orders of magnitude relative to the individual fragment affinities, and agrees well with the estimated rigid body entropy loss of 15–20 kJ/mol [72]. This example shows the reward from superadditive affinity gain by joining two weakly binding fragments with a near optimal linker.

Fragment expansion/linking, based on compound **9**, with different P1 fragments are summarized in Fig. 2. It is worth noting that the 6-methyl-3,4-dihydro-1H-quinolin-2-one P1 fragment **17** was not identified as a hit in NMR but after the expansion that resulted in compound **18** yielded a FXIa IC_50_ of 10 nM, emphasizing that fragments with very weak binding affinities can provide useful starting points for expansion. The expansion/linking of the aminoquinoline fragment **6** with the P1’-P2’ part of compound **9** resulted in compound **8** with an IC_50_ of 120 nM. The choice of the 5-position of **6** as site for initial expansion of the aminoquinoline was based on synthetic feasibility considerations. However, analyses of the aligned structures suggested that analogous expansion/linking from the 4-position might be more favorable, *i*.*e*. expanding from the methyl position in compound **19** (an equipotent analogue to compound **6**).

Analogous expansion/linking work was also performed based on compound **10** with amide bonds reversed as compared to compound **9**, see data in Fig. 2. None of the designed linked compounds, **20–24**, reached as high potency as compound **10**. However, compound **20** with an IC_50_ of 330 nM, suggests feasibility of including reverse amides in expanded structures.

The structure aided expansion/linking work described above is briefly summarized as follows: Both the X-ray structures and docking studies presented here showed that the hydrogen bond acceptor and donor positions highlighted in Fig. 7 are clearly defined in space and thus key features to utilize in modeling. The novel FXIa P1s, chloroquinolinone, dihydroquinolinone and aminoquinoline, can upon expansion yield potent and selective P1-P1’-P2’ compounds. The 4-position in chloroquinolinones and dihydroquinolinones is a favorable point for expansion. Amides and reverse amides are both acceptable, but with compounds synthesized so far, the amide nitrogen closer to the P1 part yield more potent binders.

### Synthesis

**Fig 10:**
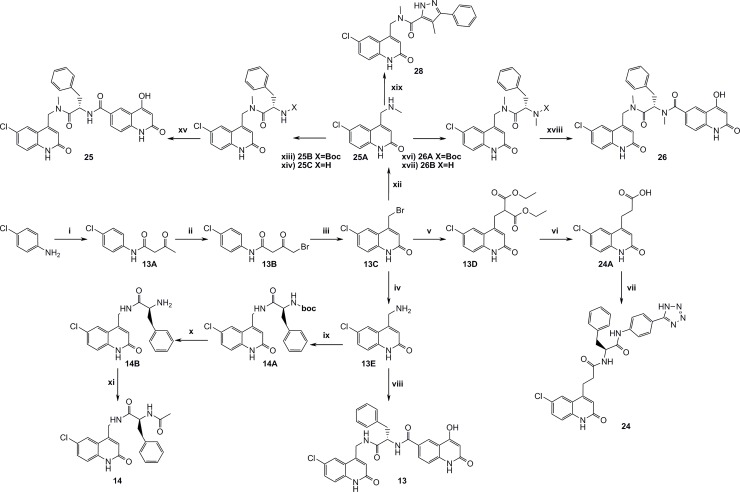
Synthesis of intermediate 13C and routes to compounds 13, 14, 25, 26 and 28. i) Methyl acetoacetat, DMAP, Pyridine, xylenes, reflux, 8h, ii) AcOH, Br2, r.t, 3h, iii) Neat H2SO4, 45°C, 46h, iv) Hexamine, DCM, reflux, 5h, then conc. HCl, reflux, 2h, v) Diethylmalonate, THF, NaH, reflux, 1h, vi) 20% HCl, reflux, 12h, vii) TBTU, TEA, DMF, 20B, r.t, 16h, viii) 15B, T3P, 13E, TEA, 120°C, 20 min., ix) HOBt, EDAC, 13E, BOC-L-phenylalanine, TEA, DMF, r.t, 16h, x) TFA, DCM, r.t, 16h, xi) AcOH, TBTU, 14B, pyridine, DMF, r.t, 16h, xii) 2M MeNH2, TEA, THF, 50°C, 1h, xiii) (S)-2-(tert-butoxycarbonylamino)-3-phenylpropanoic acid, HOBt, EDAC, DIPEA, DMF, r.t, 16h, xiv) 4M HCl in dioxane, r.t, 2h, xv) 15A, HOBt, EDAC, DIPEA, DMF, xvi) (S)-2-(tert-butoxycarbonyl(methyl)amino)-3-phenylpropanoic acid, HOBt, EDAC, DIPEA, DMF, r.t, 16h, xvii) 4M HCl in dioxane, r.t, 3h, xviii) 15A, TBTU, EDAC, DIPEA, DMSO, r.t, 48h, xix) 4-methyl-3-phenyl-1H-pyrazole-5-carboxylic acid, HOBt, EDAC, DIPEA, DMSO, r.t, 16h.

The main synthetic schemes to achieve the expanded P1-P1’ and the P1-P1’-P2’ structures are depicted in Fig. 10. One key intermediate was compound **13C** (4-(bromomethyl)-6-chloroquinolin-2(1H)-one), from which the final screen compounds **13, 14, 25, 26** and **28** were synthesized.

**Fig 11:**
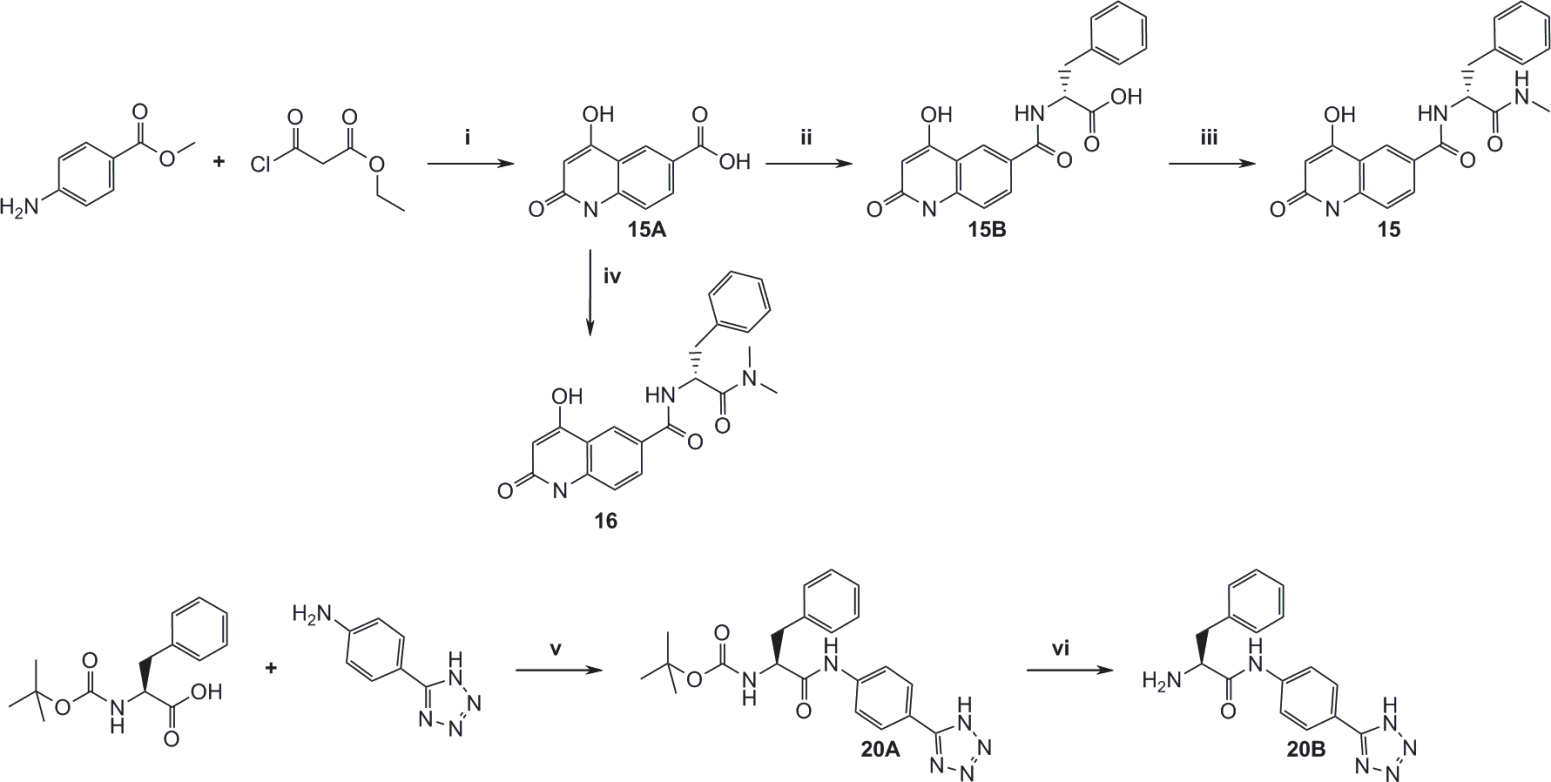
Synthesis of P1’-P2’ fragments. i) DCM, r.t, 16h, then LiOH, water, THF, r.t, 16h, then PPA, 120°C, 2h, ii) TBTU, DIPEA, DMF, L-phenylalanine methylester, r.t, 16h, iii) TBTU, pyridine, MeNH2xHCl, DMF, r.t, 16h, iv) TBTU, (S)-2-amino-N,N-dimethyl-3-phenylpropanamide hydrochloride, TEA, DMF, r.t, 16h, v) TBTU, TEA, DCM, DMF, r.t, 16h, vi) neat TFA, r.t, 0.5h.

Fig. 11 outlines the synthesis of P2’ and P1’-P2’ structures that were used for complete fragment expansion originating from P1 structures.

**Fig 12:**
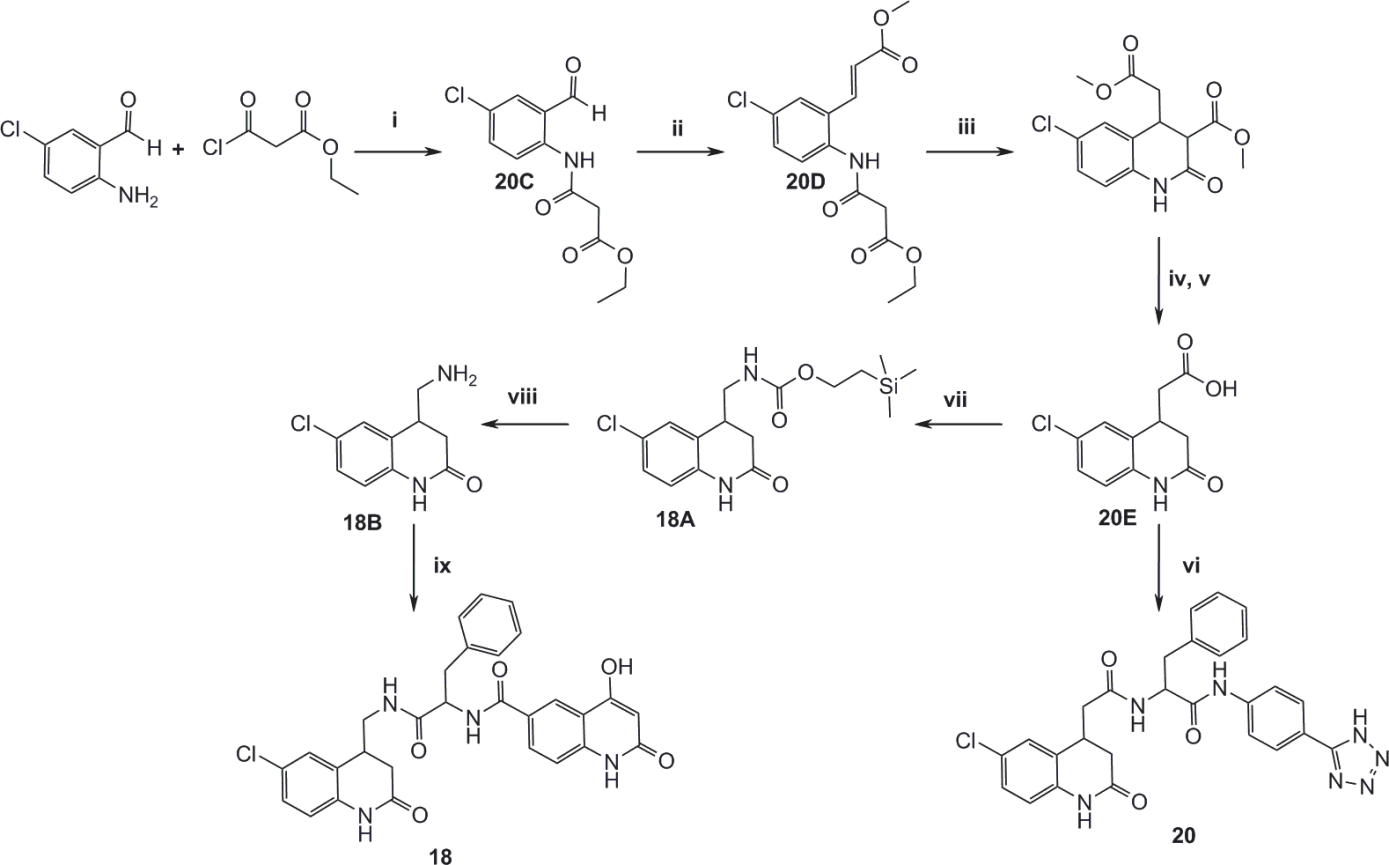
Synthesis of the dihydroquinolinone derivatives 18 and 20. i) DCM, TEA, r.t, 2h, ii) Methyl 2-(triphenylphosphoranylidene)acetate, toluene, reflux, 2h, iii) NaOMe, MeOH, r.t, 1h, iv) DMSO, water, NaCl, 150°C, 16h, v) 4M NaOH, r.t, 4h, vi) TBTU, TEA, 20B, THF, r.t, 16h, vii) DPPA, TEA, DMF, then 2-(trimethylsilyl)ethanol, 100°C, 10 min., viii) TBAF, CH3CN, 60°C, 16h, ix) 15B, TBTU, pyridine, DMF, r.t, 16h.

**Fig 13:**
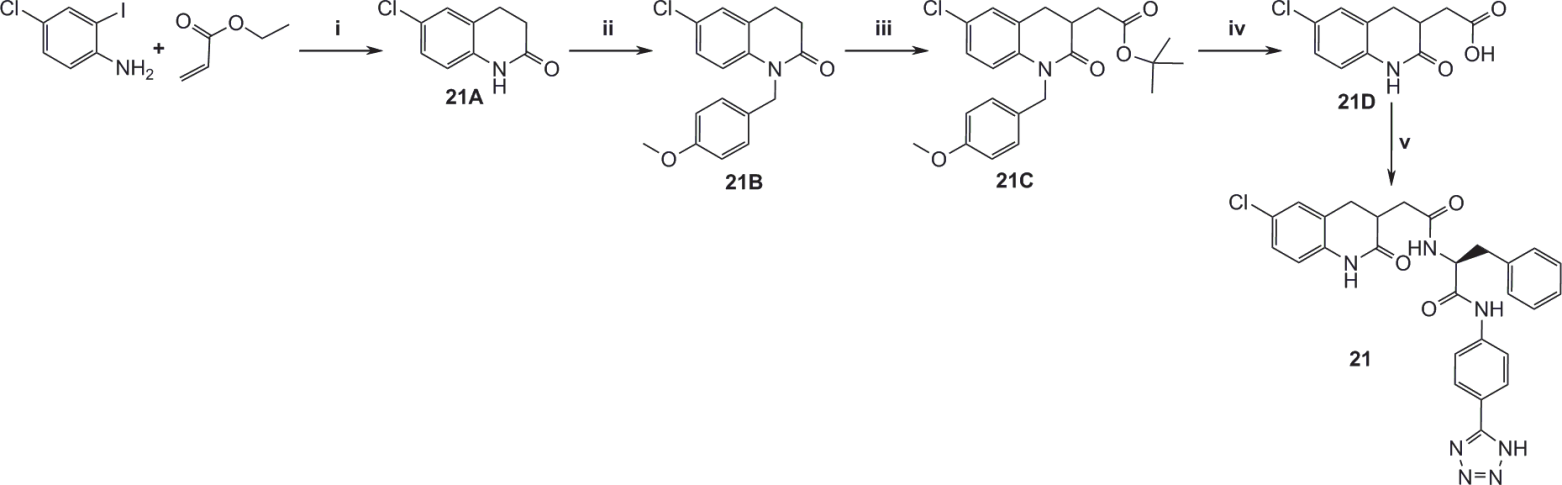
Synthesis of 3 substituted dihydroquinolinone 21. i) SnBu3H, DMSO, 100°C, 16h, ii) 4-Methoxybenzyl chloride, NaH, DMF, r.t, 2h, iii) LDA, tert-butyl 2-bromoacetate, THF, N2, -78°C, iv) Neat TFA, 80°C, 2h, v) 20B, TBTU, TEA, DMF, r.t, 16h.

**Fig 14:**
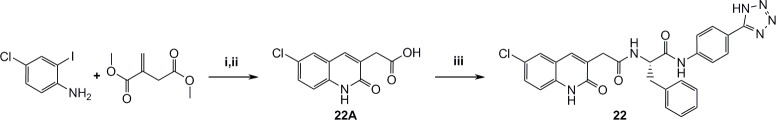
Synthesis of the 3-substituted chloroquinolinone 22. i) Pd(OAc)2, PPh3, NaOAc, dry DMF, N2, 110°C, 16h, ii) 4M NaOH, r.t, 1h, iii) 20B, TBTU, TEA, THF, r.t, 12h.

**Fig 15:**
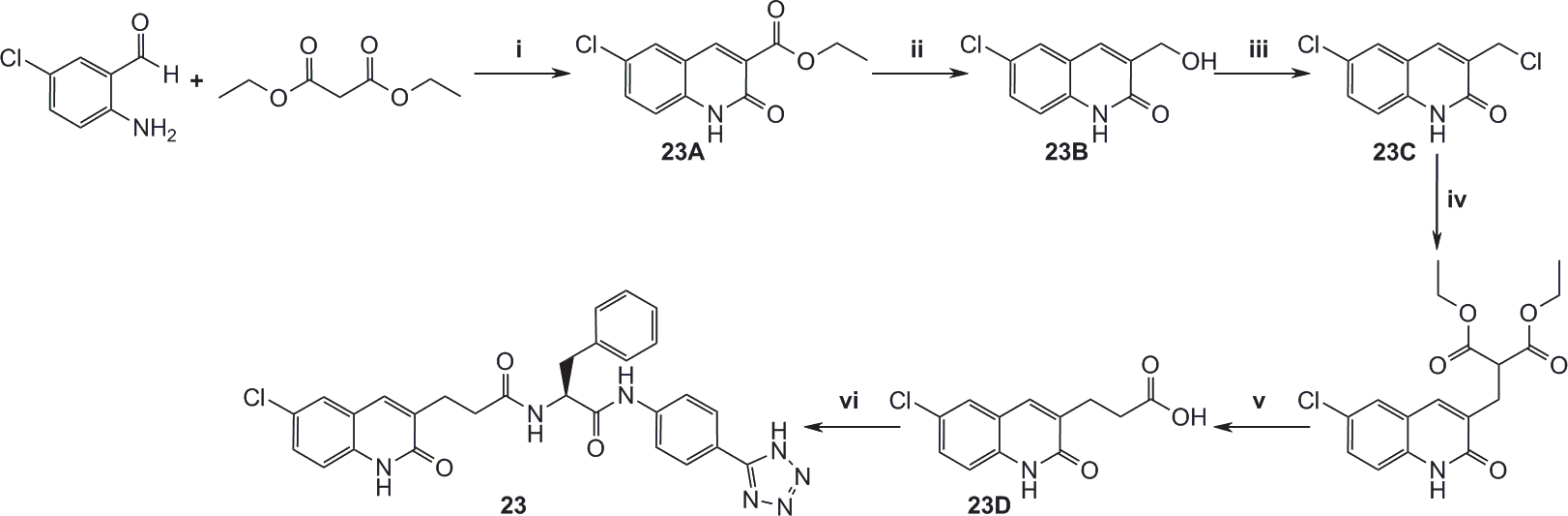
Synthesis of 3-substituted quinolinone 23. i) Piperidine, EtOH, reflux, 6h, ii) DIBAL-H, Et2O, N2, r.t, iii) Neat SOCl2, reflux, 6h, iv) DEM, NaH, THF, N2, reflux, 2h, v) Conc. HCl, reflux, 16h, vi) 20B, TBTU, DIPEA, DMF, r.t, 16h.

**Fig 16:**
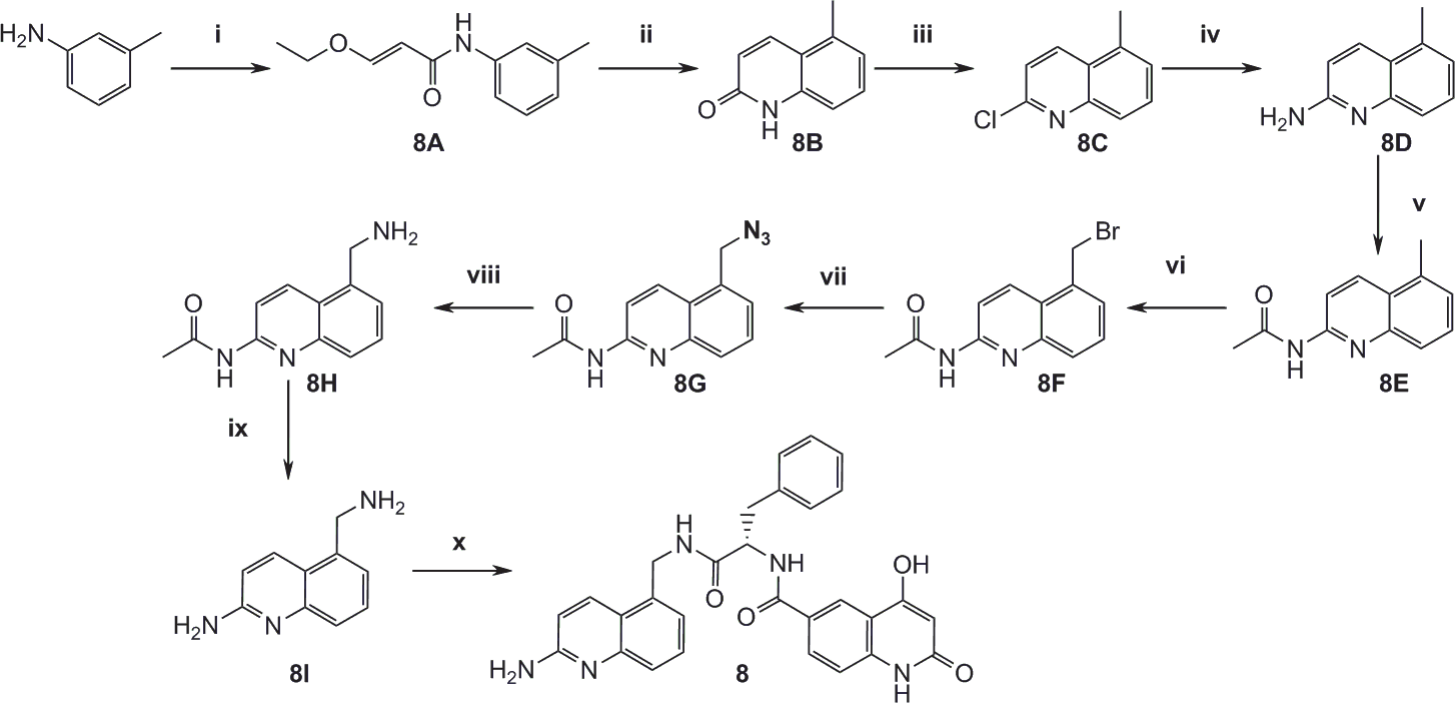
Synthesis of aminoquinoline 8. i) (E)-3-Ethoxyacryloyl chloride, THF, 50°C, 16h, ii) Conc. HCl, 40°C, 1h, iii) Neat POCl3, 80°C, 3h, iv) Xantphos, Pd2(dba)3, tBuOK, PhMe, 100°C, 1h, then 2M HCl and THF, r.t, 2h, v) AcCl, TEA, DCM, N2, 0°C, 2h, then excess MeNH2 r.t, 16h, vi) NBS, benzoyl peroxide, (trifluoromethyl)-benzene, 80°C, 16h, then reflux 2h, vii) NaN3, DMF, 80°C, 1h, viii) Pd(OH)2 on carbon, EtOH, H2, r.t, 16h, ix) 20% NaOH, 120°C, 1h, x) DMF, DIPEA, 15B, TBTU r.t, 16h.

Figs. 12–16 describe the synthetic steps to arrive at remaining screening compounds.

### Compound characterization

It is important to achieve potent FXIa inhibitors with high selectivity *vs*. other serine proteases in order to minimize risk for adverse effects, particularly with regard to bleeding, which limits the utility of current antithrombotic drugs, *e*.*g*. dabigatran and rivaroxaban. Selectivity data for FXa, thrombin, trypsin, plasma kallikrein (PKK), FIXa, tissue plasminogen activator (tPA) and plasmin were therefore generated and are summarized in Fig. 6. Assessment of fragment selectivity is limited both due to the low potencies of the fragments and that the highest compound concentration used in the assay was 99 μM. The P1’ expanded chloroquinolinone, compound **14**, with a FXIa IC_50_ of 33 μM, displayed IC_50_ values above 99 μM for all other proteases in this panel, indicating that some selectivity can be achieved through the P1-P1’ parts. The P1-P1’-P2’ compound **13** displayed more than five orders of magnitude selectivity *vs*. all proteases in the panel, except PKK where an 11-fold selectivity based on *K*i values was observed. A similar selectivity pattern was seen for compound **18** containing the chlorodihydroquinolinone P1 fragment. The full length aminoquinoline **8** displayed about 25-fold selectivity *vs*. PKK.

Regarding species selectivity, it was noted that compound **13** was about 30000-fold less potent against rat FXIa than against human FXIa. We ascribed this discrepancy primarily due to the replacement of Ala190 in human FXIa by Thr190 in rat, which makes the S1 pocket more narrow and hydrophilic and thus disfavor binding of the neutral chloroquinolinone P1. In contrast, benzamidine containing FXIa inhibitors show similar FXIa potencies between human and rat. This finding has implications on choice of species for *in vivo* studies and highlights the importance of doing species analyses early in projects.

**Fig 17:**
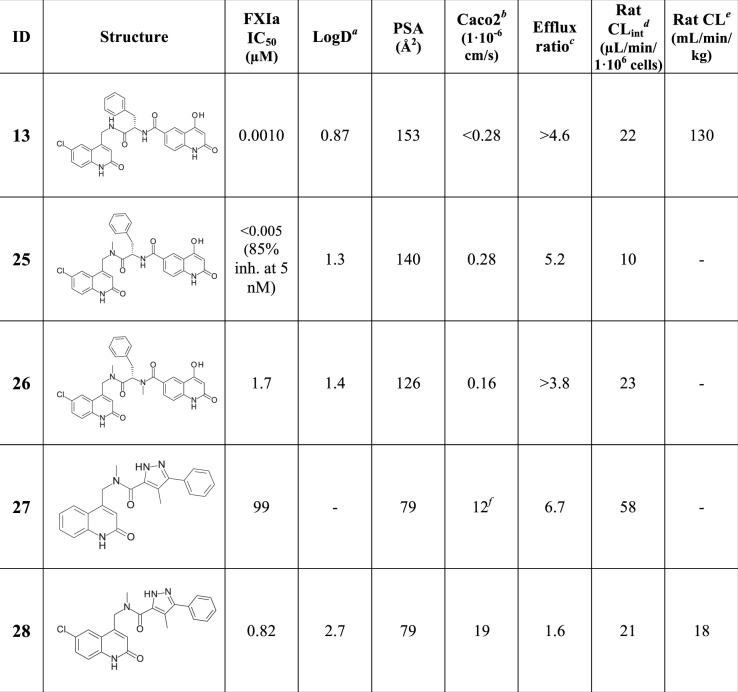
Structures with associated FXIa potencies and selected physicochemical and ADME parameters. ^*a*^logD measured by liquid chromatography. ^*b*^Caco2 A to B permeability measured at pH 6.5. ^*c*^Efflux ratio determined in Caco2 cells by dividing P_app_ B to A with P_app_ A to B permeabilities measured at pH 7.4 at side A. ^*d*^Rat intrinsic clearance determined from measurements in rat hepatocytes. ^*e*^Rat *in vivo* clearance. ^f^Caco2 A to B permeability measured at pH 7.4 at side A.

The next stage of the lead identification work focused on a more balanced approach to address not only potency and selectivity but also physicochemical and ADME properties. As seen in Fig. 17, compound **13** displays high polar surface area (PSA), low permeability, high efflux and high *in vivo* clearance. The high rat *in vivo* clearance despite the low/moderate *in vitro* intrinsic clearance in rat hepatocytes may suggest active transport into bile and/or urine *in vivo*. We hypothesized that, relative to compound **13**, improved permeability and decreased efflux should increase bioavailability and lower clearance *in vivo*. Initial ADME exploration targeted minor modifications of compound **13** to first methylate the P1-P1’ amide nitrogen, as in compound **25**, and subsequently adding a second methyl group on the P1’-P2’ amide nitrogen, yielding compound **26**. Minor improvements in permeability and efflux were observed with compound **25**, and compound **26** provided no further benefit. From the structure of FXIa in complex with compound **11**, the methyl group on the P1-P1’ amide in compound **25** is predicted to point out towards the solution, whereas the methyl group on the P1’-P2’ in compound **26** is predicted to disturb important hydrogen bond interactions with the tightly bound water molecule shown in Fig. 7 and would therefore be detrimental for potency. The IC_50_ values of <0.005 and 1.7 μM for compound **25** and **26**, respectively, support this notion. After the methylation exercise, it was decided to keep the methylated P1-P1’ amide as first step expansion of the P1 fragment and search for further expansion replacing the P1’-P2’ amide with less hydrophilic structures. In this context, 13 compounds available in our corporate compound collection with structures containing the N-methyl-N-[(2-oxo-1H-quinolin-4-yl)methyl]formamide scaffold were screened. Compound **27** with a FXIa IC_50_ of 99 μM was identified, which displayed high permeability (see Fig. 17), but still showed relatively high efflux. The low potency of compound **27** was improved by approximately two orders of magnitude upon addition of the chloro atom to the aminoquinolinone P1 to afford compound **28** with an IC_50_ of 0.82 μM. The ADME properties of compound **28** with high permeability (Caco2 A to B = 19 · 10^–6^ cm/s), moderate rat *in vivo* clearance (18 mL/min/kg) and a measured oral bioavailability in rat of 27% show promise towards the goal of developing an oral drug. Potency improvements of this lead with maintained favorable ADME properties remain to be shown. However, comparing the potencies of the P1-P1’ compounds **28** and **14** (IC_50_ values of 0.82 and 33 μM, respectively), and realizing the potency boost feasible by adding a P2’ moiety (compare compounds **14** and **13** in Fig. 9), suggest exciting opportunities for development of a potent, selective and oral FXIa inhibitor drug.

## Summary and Conclusion

A virtual screen based on docking experiments was performed to select a fragment library, and NMR was used as the primary screen with minimal risk of false positives. SPR and enzymatic screening quantified the affinities of the NMR hits, and X-ray structures provided the basis for fragment expansion and linking design.

The structure aided fragment based lead generation strategy adopted in this work successfully identified two novel FXIa P1 fragments that were expanded towards the S1’-S2’ pockets to yield potent and selective inhibitors. The chloroquinolinone is neutral and the aminoquinoline is only weakly basic and should provide opportunities to be developed into bioavailable full length FXIa inhibitors without relying on a prodrug approach. It remains to be explored whether the FXIa chloroquinolinone and aminoquinoline P1s will have more general utility as serine protease P1s. The very potent and selective compound **13** gives promise for further optimization. The future challenge will be to improve the physicochemical properties of the chloroquinolinone and aminoquinoline P1 based compounds presented here to optimize the combined potency, selectivity, bioavailability and clearance profiles. Compound **28** indicates progress towards this goal. A success in this endeavor could be well rewarded in the form of an effective and safe antithrombotic drug.

## Materials and Methods

### Protein expression and purification

Protein expression and purification of the catalytic domain (residues 370–607) was carried out as described by Jin et al. [59]. To improve crystallisability this protein contains the four mutations: S434A, K437A, T475A and C482S (S75A, K78A, T115A, C123S, trypsin numbering). This protein was used for crystallisation, NMR and SPR measurements.

### NMR screening

NMR samples were prepared in aqueous buffer containing 50 mM deuterated TRIS, pH 7.4, 3 mM NaN_3_, and 10% D_2_O. FXIa CD stock solution was buffer exchanged and concentrated to approximately 100 μM (2.7 mg/mL) (UV absorption ε_280_ = 60390 cm^-1^) using 10 kDa cut-off Millipore filter centrifugation tubes. Control experiments with pMeBza and compound **2** were done on all protein preparations (Fig. 3). Stock solutions of test compounds were prepared in randomised mixtures of six by pooling 10 μL aliquots of 10 mM DMSO solutions from the AZ compound library in microtiter plates followed by vacuum evaporation of the solvent. The solid compound mixtures were then redissolved in 20 μL DMSO-d_6_ yielding 5 mM compound stock solutions. Samples for retesting of individual compounds were prepared from original compound library solution in regular DMSO, necessitating the simultaneous suppression of solvent and DMSO peaks (see below). Samples were prepared “just in time” using a Genesis liquid handling robot (TECAN, CH) and transferred to the NMR spectrometer via a SampleRail system (Bruker Biospin, DE). Mixtures were screened at 100 μM compound concentration in aqueous buffer with 5 μM FXIa CD added in the second step. At the third step, 10 μM inhibitor (compound **2**) was added.

NMR data were collected at 20^o^C on a Bruker Avance III 600 MHz NMR spectrometer equipped with a cryo probe head. 1D ^1^H *T*_1ρ_ NMR spectra [56] were recorded with 256 scans, 3.2 s interscan delay, 180 ms spin lock time and 64 kHz spin lock field. Solvent suppression was achieved by applying an 8 ms excitation sculpting sequence [58] before acquisition. For samples prepared from non-deuterated DMSO stock solutions, simultaneous suppression of the solvent and DMSO peaks was achieved by replacing the selective 180 degree flip back pulses with bilinear pulses created with the Pulse tool module in TopSpin (Bruker, CH). Spectra were analyzed in TopSpin and compounds were considered hits if they displayed significant signal amplitude changes upon addition of protein and inhibitor.

### FXIa surface plasmon resonance assay

The tethered free amine ligand **4** was covalently linked to the dextran layer on the CM5 sensor chip (GE Healthcare/Biacore) using the Amine Coupling Kit (Healthcare/Biacore) and performed according to the manufacturer’s recommendations. Carboxyl groups on the dextran layer were activated by injecting a 1:1 mixture of 0.5 M N-ethyl-N’ (3-dimethylaminopropyl)-carbodiimide and 0.5 M N-hydroxysuccinimide at 10 μL/min for 7 min. The activated carboxyl groups on the control channels were blocked by injecting a 1 M ethanolamine solution for 7 min at 10 μL/min. The tethered ligand was coupled to the enzyme binding channels by injecting a 100 μM solution of the compound (10 mM 4-(2-hydroxyethyl)-1-piperazineethanesulfonic acid, pH 7.4, 0.15 M NaCl, 0.005% (v/v) polysorbate 20, 2% DMSO) over the chip surface at 10 μL/min for 1 min. The remaining activated groups were blocked with 1 M ethanolamine. Compounds were tested in a concentration response curve. The compounds were serially diluted 1:3 in DMSO to form a 10-point concentration response curve with 5 mM for the highest concentration. The FXIa CD was added to the plate at a final concentration of 50 nM and the mixture was incubated for 15 min at RT. The plate was then sealed and placed in the Biacore 3000 instrument. Samples were injected at 20 μL/min for 2 min. The signal generated on the control channel was subtracted from the signal of the channel with the tethered ligand. The value used to calculate percent inhibition of binding was the slope of the response measured 8 s after the start of the injection. The surface of the chip was regenerated by injecting 0.5% SDS for a period of 36 s.

### Crystallisation and data collection

In order to obtain ligand complexes with compounds **5**, **6**, **8**, **10**, **11**, **12** and **13**, co-crystallisation was used. FXIa (≈ 1 mg/ml in 20 mM Tris pH 7.4, 200 mM NaCl) was mixed with respective compound to a concentration of 1–5 mM and incubated at room temperature for around 1 h. The complexes were then concentrated to about 20 mg/ml. Crystallisations was set up at 20°C using the hanging-drop vapour diffusion method with the precipitation solution 1.8–2.2 M (NH_4_)_2_SO_4_ and 0.1 M Tris pH 8.5. Cubic shaped crystals appeared after a few days. For cryo protection a quick dip in 3.1 M (NH_4_)_2_SO_4_ and 0.1 M Tris pH 8.5 was done for compound **5**, **6**, **8**, **12** and **13** and in 20% glycerol, 2 M (NH_4_)_2_SO_4_ and 0.1 M Tris pH 8.5 for compound **10** and **11** before flash-freezing in liquid nitrogen. For compound **5**, **6**, **8**, **12** and **13** data was collected on a Rigaku FRE+ rotating anode with a CCD-A200-CU detector. Data for compound **10** was collected at the ID23–1 beam line and data for **11** was collected at the ID14–4 beam line of the European Synchrotron Radiation Facility in Grenoble, France. Summary of data collection parameters and statistics are listed in Table 1.

**Table 1:** X-ray crystallography.

	FXIa:compound **1**	FXIa:compound **5**	FXIa:compound **6**
PDB ID	4CRG	4CR5	4CR9
Space group	I23	I23	I23
Cell dimensions (Å)	*a = b = c =* 120.9	*a = b = c =* 121.0	*a =* 121.3
Resolution (Å)	49.3–1.3 (1.33–1.25)^1^	24.7–2.0 (2.05–2.0)^1^	21.5–1.7 (1.74–1.7)^1^
*R*_merge_	0.071 (0.745)	0.153 (0.623)	0.079 (0.83)
<*I* / σ*I*>	14.8 (1.8)	11.1 (3.3)	15.2 (2.4)
Completeness (%)	92.8 (75.0)	99.9 (99.9)	100.0 (100.0)
Redundancy	5.2 (2.5)	8.7 (8.1)	7.7 (7.2)
Refinement			
Measured / unique refl.	388302/75396	174104 / 20045	236553 / 32745
*R*_work_ / *R*_free_	0.176/ 0.195	0.203 / 0.247	0.179 / 0.208
No. atoms			
Protein	1986	1894	1914
Water/ Ligand	333/58	197/12	298/13
Average *B*-factors			
Protein (Å^2^)	18.3	14.8	18.3
Water (Å^2^)	36.0	21	29.8
Ligand (Å^2^)	18.4	17.2	16.6
R.m.s deviations			
Bond lengths (Å)	0.010	0.008	0.006
Bond angles (°)	1.1	1.2	1.1
	FXIa:compound **8**	FXIa:compound **9**	FXIa:compound **10**
PDB ID	4CRA	4CRB	4CRC
Space group	I23	I23	I23
Cell dimensions (Å)	*a = b = c = 121.1*	*a = b = c = 120.8*	*a = b = c = 117.8*
Resolution (Å)	23.8–1.8 (1.85–1.80)^1^	50–1.85 (1.9–1.85)^1^	41.6–1.6 (1.64–1.60)^1^
*R*_merge_	0.087 (0.943)	0.08 (0.63)	0.08 (0.66)
<*I* / σ*I*>	12.3 (1.6)	13.9 (2.2)	12.9 (2.6)
Completeness (%)	99.1 (98.1)	99.5 (100.0)	99.6 (100.0)
Redundancy	4.6 (4.3)	5.1 (5.1)	7.0 (7.0)
Refinement			
Measured / unique refl.	125895 / 27223	127473 / 25023	248924 / 35732
*R*_work_ / *R*_free_	0.177 / 0.204	0.19 / 0.213	0.185 / 0.213
No. atoms			
Protein	1918	1881	1918
Water/Ligand	215/38	122/40	195/39
Average *B*-factors			
Protein (Å^2^)	23.0	28.6	21.8
Water (Å^2^)	32.5	34.8	32.4
Ligand (Å^2^)	21.9	25.5	17.1
R.m.s deviations			
Bond lengths (Å)	0.007	0.007	0.006
Bond angles (°)	1.1	1.2	1.2
	FXIa:compound **11**	FXIa:compound **12**	FXIa:compound **13**
PDB ID	4CRD	4CRE	4CRF
Space group	I23	I23	I23
Cell dimensions (Å)	*a = b = c = 120.5*	*a = b = c =* 121.1	*a = b = c = 121.3*
Resolution (Å)	50–2.1 (2.15–2.10)^1^	23.8–1.7 (1.77–1.73)^1^	32.4–2.3 (2.36–2.30)^1^
*R*_merge_	0.082 (0.508)	0.068 (0.559)	0.227 (1.12)
<*I* / σ*I*>	11.4 (2.4)	21.4 (2.9)	9.5 (2.1)
Completeness (%)	99.5 (100.0)	99.9 (98.7)	100.0 (100.0)
Redundancy	5.8 (4.3)	9.3 (6.3)	9.9 (9.5)
Refinement			
Measured / unique refl.	98743 / 17077	288638 / 31069	131774 / 13350
*R*_work_ / *R*_free_	0.203 / 0.234	0.172 / 0.197	0.181 / 0.244
No. atoms			
Protein	1881	1928	1892
Water/Ligand	76/35	287/13	95/39
Average *B*-factors			
Protein (Å^2^)	41.7	17.2	28.6
Water (Å^2^)	39.9	29.0	29.2
Ligand (Å^2^)	32.9	21.0	22.6
R.m.s deviations			
Bond lengths (Å)	0.010	0.005	0.011
Bond angles (°)	1.4	1.1	1.4

Data collection and refinement statistics.

^1^Values in parentheses refer to highest-resolution shell.

For obtaining ligand complexes with compounds **1** and **9**, powder soaks to preformed FXIa-benzamidine crystals were performed. FXIa-benzamidine crystals were obtained by mixing diluted FXIa (≈ 1 mg/mL in 20 mM Tris pH 7.4, 200 mM NaCl) with benzamidine to a concentration of 10 mM and incubating at room temperature for 1 h. The complex was concentrated to ≈ 20 mg/mL and crystallisation set up with the precipitate solution of 1.8–2.2 M (NH_4_)_2_SO_4_ and 0.1 M Tris pH 8.5. Single crystals were transferred to a solution consisting of ≈ 2 M (NH_4_)_2_SO_4_ and 0.1 M Tris pH 8.5 to which excess of compound powder was added. The soaking lasted for 24 h for compound **9** and 10 days for compound **1**. The crystals were briefly soaked in a cryoprotection solution containing 20% glycerol, 2 M (NH_4_)_2_SO_4_ and 0.1 M Tris pH 8.5 before being flash-frozen in liquid nitrogen. The data was collected at the ID14–4 beam line of the European Synchrotron Radiation Facility in Grenoble, France. A summary of data collection parameters and statistics are listed in Table 1.

### Data processing and structure determination

The crystals belong to the spacegroup I23 with one copy of the FXIa CD in the asymmetric unit. The data for the FXIa:compound **1** complex was processed using autoPROC [73] and XDS [74] and scaled with Aimless [75]. All other data was processed with the programs Mosflm [76] and Scala [75,77]. Structure determination by molecular replacement was conducted with the program Molrep [75,78], Phaser [79] or rigid body refinement with Refmac [75,80], using an in house high-resolution crystal structure as search model. Rebuilding of the model and addition of water molecules were done in the program Coot [62]. The structure of the FXIa:compound 1 complex was refined with Refmac and autoBUSTER (BUSTER version 2.11.5. Cambridge, United Kingdom: Global Phasing Ltd) and the final protein model comprised residues 16–130 and 133–244 (chymotrypsin numbering). Compound **1** was well defined in the electron density, except for the benzyl group which was modelled in two different orientations, the predominant orientation is depicted in Fig. 4. All other datasets were refined using Refmac [80] and all protein residues, 16–245, were included in the final models. The ligands were placed in the F_o_F_c_ difference maps during the last steps of refinement. Details from data collection, refinement and the final models are summarised in Table 1. The atomic coordinates and structure factors have been deposited in the protein data bank with accession codes 4CR5, 4CR9, 4CRA, 4CRB, 4CRC, 4CRD, 4CRE, 4CRF and 4CRG.

### Enzymatic assays

Once the compounds were synthesized and characterized, enzymatic assays were performed to evaluate the inhibitory activity of the resulting compounds against FXIa and other serine proteases. All protease activities were determined by chromogenic assays monitoring the release of para-nitroanilide at 405 nm. IC_50_ values for the compounds were determined using equation:

$$\text{Q}=\text{ V}/\left(\text{1}+{\left(\left[\text{I}\right]/{\text{IC}}_{\text{5}0}\right)}^{\text{n}}\right)$$

Q denotes the assay readout, V denotes the uninhibited velocity of the enzyme, [I] denotes the inhibitor concentration, n denotes the slope and IC_50_ is the inhibitor concentration at V/2.

The IC_50_ values were converted into *K*_i_ values for competitive inhibitors using the equation:

$$K_{\text{i}}={\text{ IC}}_{\text{5}0}/\left(\text{1 }+\left[\text{S}\right]/{\text{K}}_{\text{m}}\right)$$

and the respective K_m_ values for the substrate in the assay. [S] denotes the substrate concentration.

**FXIa**. Human FXIa was from HTI, Essex Junction, USA. Inhibition by compounds was measured using 420 μM pyroGlu-Pro-Arg-pNA·HCl (Bachem, Bubendorf, Switzerland, K_m_ = 420 μM) as substrate in 50 mM Tris pH 7.4, 100 mM NaCl, 5 mM CaCl_2_, 0.1 mg/mL BSA, 1% DMSO (v/v).

**Trypsin**. Recombinant human trypsin was from Polymun, Vienna, Austria. Inhibition by compounds was measured using 300 μM Bz-Val-Gly-Arg-pNA (Bachem, Bubendorf, Switzerland, K_m_ = 230 μM) as substrate in 50 mM Tris pH 7.4, 40 mM NaCl, 20 mM CaCl_2_, 0.1% Tween 20 (v/v), 1% DMSO (v/v).

**Thrombin**. Human recombinant thrombin was prepared in house. Inhibition by compounds was measured using 300 μM pyroGlu-Pro-Arg-pNA·HCl (Km = 280 μM) as substrate in 50 mM Tris/HCl pH 7.4, 100 mM NaCl, 0.2% BSA (w/v), 1% DMSO (v/v).

**Plasmin**. Human plasmin was from Enzyme Research Laboratories, Swansea, UK. Inhibition by compounds was measured using 300 μM pyroGlu-Pro-Arg-pNA·HCl (K_m_ = 275 μM) as substrate in 50 mM Tris pH 7.4, 100 mM NaCl, 0.1% Tween 80 (v/v), 1% DMSO (v/v).

**FIXa**. Human FIXa was from Enzyme Research Laboratories, Swansea, UK. Inhibition by compounds was measured using 500 μM CH_3_SO_2_-(D)-CHG-Gly-Arg-pNA·AcOH (HTI, Essex Junction, USA, K_m_ = 1300 μM) as substrate in 33 mM Tris/HCl pH 7.4, 66 mM NaCl, 3.3 mM CaCl_2_, 33% Ethylene glycol (v/v), 0.3% BSA (w/v), 1% DMSO (v/v).

**FXa**. Human FXa was from HTI, Essex Junction, USA. Inhibition by compounds was measured with 100 μM Z-D-Arg-Gly-Arg-pNA (Bachem, Bubendorf, Switzerland, K_m_ = 50 μM) as substrate in 50 mM Tris pH 7.4, 100 mM NaCl, 5 mM CaCl_2_, 0.2% BSA (w/v), 1% DMSO (v/v), 1% DMSO (v/v).

**tPa**. Human tPA was from Biopool, Umeå, Sweden. Inhibition by compounds was measured with 250 μM CH_3_SO_2_-D-HHT-Gly-Arg-pNA (Pentapharm, Basel, Switzerland, K_m_ = 140 μM) in 50 mM Tris/HCl pH 7.4, 100 mM NaCl, 0.032% Tween 80 (v/v), 1% DMSO (v/v).

**PKK**. Human PKK was from Calbiochem, Darmstadt, Germany. Inhibition by compounds was measured with 400 μM D-Pro-Phe-Arg-pNA·HCl (Hyphen Biomed, Neuville-Sur-Oise, France, Km = 105 μM) in 50 mM Tris/HCl pH 7.4, 100 mM NaCl, 5 mM CaCl2, 0.2% BSA (v/w), 1% DMSO (v/v).

### Chromatographic determination of lipophilicity

The chromatographic data was obtained utilizing a Waters Aquity UPLC chromatographic system running a gradient from 5–95% acetonitrile at pH7.4. The stationary phase was a Waters BEH C18 2.1x50 mm. Standardized retention data were obtained by normalization against a set of compounds with precisely determined (octanol/water) logD-values. Lipophilicity values for samples were derived from a corresponding calibration curve.

### Caco2 permeability and efflux

A monolayer of Caco2 cells, cultured on semi-permeable polycarbonate surfaces, was used to study the permeability in the apical to basolateral direction. The process was atomized by a robotic Tecan EVO platform (Männerdorf, Switzerland) and 24-transwell plates from Costar (Cambridge, MA, USA). HBSS buffer pH 7.4 was dispensed to the basal side of the monolayer. The assay was initiated by adding the test substrate (10 **μ**mol/l in HBSS buffer pH 6.5) to the apical side of the monolayer. Samples for quantitative analysis by LC/MS/MS were withdrawn before the addition of the test substrate and at 45 and 120 min post addition of the test substrate. During incubation the transwell plates were placed in a shaking incubator at 37°C between sampling. The peak areas were exported to Excel where Papp-values and recoveries were calculated. Twenty-two reference compounds with known human oral bioavailability were used to establish a correlation curve with P_app_ versus the fraction of the oral dose absorbed (f_a_).

Efflux was studied in the same Caco2 cells with the modification that HBSS buffer pH 7.4 was used on both sides of the monolayer. The permeability was studied in the apical to basolateral direction (P_app_ AB) as well as in the basolateral to apical direction and (P_app_ BA). Efflux ratios were derived from the following equation: P_app_ BA/P_app_ AB.

### Rat hepatocyte metabolic stability assay

Rat hepatocyte metabolic stability was determined in accordance with the method described by Jacobson et al. [81]. Cryo preserved hepatocytes at a concentration of 10^6^ viable cells/mL were used. After thawing, hepatocytes were incubated for 10 min to warm to 37°C and test compounds, dissolved in acetonitrile, were added to give a final concentration of 1 μM. At 0.5, 5, 15, 30, 45, 60, 80, 100 and 120 min, the incubation system was mixed and 20 μL aliquots were transferred at each time point to wells in a separate plate filled with 80 μL acetonitrile to stop the reaction. The quenching plate was then vortexed followed by centrifugation, and supernatants were analyzed by LC/MS/MS. Peak areas were determined from extracted ion chromatograms, and the *in vitro* intrinsic clearance (*in vitro* CL_int_, in μL/min/10^6^ cells) of parent compound was calculated from the slope in the regression analysis of the natural logarithm of parent concentration *vs*. time curve.

### *In vivo* rat PK

Two days prior to dosing, female Sprague Dawley rats were prepared by cannulation of the left carotid artery for blood sampling and by cannulation of the right jugular vein for intravenous administration. The catheters were filled with heparin (100 IU/mL), exteriorized at the nape of the neck and sealed. The surgery was performed under isoflurane (Forene®, Abbott) anesthesia. After surgery the rats were housed individually and had free access to food and water. About 16 h prior to dosing the animals were deprived of food, and fasted until 4 h after dosing. The rats had free access to drinking water during the experiment. On the experiment day, the test item formulation was administered orally by gavage or intravenously in the jugular vein. At pre-defined time points, blood samples of about 0.150 mL were withdrawn from the carotid artery up to 24h after dosing. A total of 10 samples were withdrawn. The blood samples were collected in heparinized plastic tubes and centrifuged, within 30 min, for five min at 10 000 g and +4°C. The plasma was transferred to a 96 well plate and stored at -20°C until analysis by LC/MS/MS. The studies were approved by the Göteborg Animal Research Ethical Board.

### Chemistry

Chemicals and solvents from commercially available sources was purchased and used without further purification. ^1^H NMR spectra were recorded on a Bruker biospin GmbH 400, 500 or 600 MHz spectrometer. Chemical shifts are reported in parts-per-million (δ) relative to DMSO-d_6_ at 2.50 ppm, CDCl_3_ at 7.26 ppm or CD_3_OD at 3.31 ppm as an internal standard. High-resolution mass spectrometry (HRMS) analysis was performed using a Waters XEVO-qTOF instrument (Waters, Milford, USA) with MassLynx 4.1 software. If necessary, the purity was determined by ultra high performance liquid chromatography (U(H)PLC). Purity of all final compounds was 95% or higher at 210 nm. The instrument was a Waters U(H)PLC Acquity system (Binary pump, Sample Manager, Column Compartment and UV-PDA detector). The column was a Waters Acquity BEH C18, 1.7 μm particle size (100 mm × 2.1 mm).

Heating of reactions with microwaves was performed on a Biotage Initiator 2.45 GHz, 400W microwave for the times indicated. Compounds **1**, **2**, **4** and **27** were obtained from the AstraZeneca corporate compound collection, compounds **3**, **5**, **6**, **7**, **12**, **17**, and **19** were commercially available and compounds **9** [31], **10** [31] and **11** [30] were synthesized according to literature. Experimental and spectroscopic details for all other non-commercially available compounds are given below or are available in S1 File.

In general, optimizations of the reaction procedures were not performed and yields are given for isolated materials.

**N-(3-(2-(2-aminoethoxy)ethoxy)phenyl)-6-carbamimidoyl-4-(pyrimidin-2-ylamino)-2-naphthamide (4)**. Compound 4 was synthesized in an 8 step sequence from methyl 6-cyano-2-naphthoate as described in the S1 File. ^1^H NMR (500 MHz, CD_3_OD) δ 3.16 (s, 2H), 3.74–3.87 (m, 2H), 3.92 (s, 2H), 4.22 (d, 2H), 6.78 (d, 1H), 6.90 (t, 1H), 7.22 (d, 1H), 7.29 (t, 1H), 7.61 (s, 1H), 7.91 (d, 1H), 8.27 (d, 1H), 8.38 (s, 1H), 8.43–8.55 (m, 3H), 8.76 (s, 1H).

**(E)-3-Ethoxy-N-m-tolylacrylamide (8A)**. To a solution of (E)-3-ethoxyacryloyl chloride (8.20 g, 60.9 mmol) in THF (275 mL) was added 3-Methylaniline (13.1 g, 122 mmol), forming a pale orange precipitate. The reaction mixture was heated at 50°C for 16 h. The reaction was allowed to cool to room temperature and then filtered. The filtrate contained crude compound 8A (12.5 g, 100%) sufficiently pure to be used in the next reaction step without further purifications. ^1^H NMR (400 MHz, CDCl_3_) δ 1.24–1.32 (3H, m), 2.28 (3H, d, *J* = 6.9 Hz), 3.82 (2H, q, *J* = 7.1, 7.1, 7.1 Hz), 5.38 (1H, d, *J* = 12.1 Hz), 6.87 (1H, d, *J* = 7.5 Hz), 7.15 (1H, t, *J* = 7.8, 7.8 Hz), 7.32 (1H, d, *J* = 7.8 Hz), 7.39 (1H, s), 7.59 (1H, d, *J* = 12.1 Hz), 7.67 (1H, d, *J* = 16.3 Hz).

**5-Methylquinolin-2(1H)-one (8B)**. (E)-3-Ethoxy-N-m-tolylacrylamide (8A) was dissolved in warm (40°C) concentrated HCl (40 mL) and stirred for 1 h, allowing the reaction to slowly reach room temperature. The reaction mixture was then poured into a beaker with crushed ice, forming an orange precipitate. The precipitate was isolated by filtration, washed with cold water and then dried under reduced pressure. The crude solid contained a mixture of regioisomers (40:60, 3.60 g, 41%) with the desired product in minority. The regioisomer mixture was separated by preparative HPLC to give the desired regioisomer 8B (0.601 g, 17%). ^1^H NMR (400 MHz, CDCl_3_) δ 2.44 (3H, s), 6.63 (1H, d, *J* = 9.4 Hz), 7.03 (1H, d, *J* = 7.4 Hz), 7.14 (1H, s), 7.43 (1H, d, *J* = 8.0 Hz), 7.76 (1H, d, *J* = 9.5 Hz), 11.35 (1H, s).

**2-Chloro-5-methylquinoline (8C)**. 5-Methylquinolin-2(1H)-one (8B) (0.50 g, 3.15 mmol) was heated in POCl_3_ (7.43 g, 48.4 mmol) at 80°C for 3 h. The reaction was allowed to reach room temperature and poured into a beaker containing crushed ice and stirred until all ice had melted. Resulting suspension was extracted with chloroform (3 x 15 mL). The combined organic fractions was dried (MgSO_4_), filtered, and then concentrated under reduced pressure to afford compound 8C as a crude black solid (0.480 g, 87%).

**5-Methylquinolin-2-amine (8D)**. A mixture of 2-chloro-5-methylquinoline 8C (0.484 g, 2.72 mmol), Xantphos (0.126 g, 0.220 mmol), tris(dibenzylideneacetone)dipalladium (0.100 g, 0.110 mmol) and potassium *tert*-butoxide (0.917 g, 8.17 mmol) in Toulene (15 mL) was heated at 100°C for 1 h using a microwave reactor. The reaction was quenched with water and the phases separated. The aqueous phase was extracted with DCM (3 x 20 mL), the combined organic fractions was passed through a phase separator to remove water and palladium residues. The filtrate was concentrated under reduced pressure, the residue re-dissolved in a mixture of THF (10 mL) and 2M HCl (10 mL), and stirred at room temperature for 2 h. The reaction was concentrated under reduced pressure to give compound 8D as a crude solid (0.427 g, 99%). The crude product was used in the next reaction step without further purifications.

**N-(5-Methylquinolin-2-yl)acetamide (8E)**. A mixture of 5-methylquinolin-2-amine (8D) (0.427 g, 2.72 mmol) and triethylamine (TEA) (3.79 mL, 27.2 mmol) in DCM (10 mL) was stirred at 0°C under an inert atmosphere of nitrogen. Acetyl chloride (1.94 mL, 27.2 mmol) was added drop wise and resulting mixture stirred for 2 h. A large excess methylamine (2M in THF) was then added and resulting mixture stirred for 16 h to cleave di-acetylated products. The reaction was concentrated under reduced pressure and resulting residue filtered through a plug of silica using EtOAc/ heptane (1:3) as the eluent. The crude compound 8E (0.270 g, 50%) was sufficiently pure to be used in the next reaction step without further purifications.

**N-(5-(Bromomethyl)quinolin-2-yl)acetamide (8F)**. A solution of N-(5-methylquinolin-2-yl)acetamide 8E (0.270 g, 1.35 mmol), NBS 0.528 g, 2.97 mmol), benzoyl peroxide (0.098 g, 0.400 mmol) was dissolved in (trifluoromethyl)-benzene (6 mL) and the resulting mixture heated at 80°C for 16 h. The reaction proceeded slowly and was heated at reflux for 2 h to achieve complete conversion of the starting material. The reaction was diluted with water and then extracted with DCM (3 x 15 mL). The combined organic fractions was dried (MgSO_4_), filtered and then concentrated to give compound 8F as a crude pale brown oil (0.377 g, 100%). The crude material was used without further purifications in the next reaction step.

**N-(5-(Azidomethyl)quinolin-2-yl)acetamide (8G)**. A solution of N-(5-(bromomethyl)quinolin-2-yl)acetamide 8F (0.377 g, 1.35 mmol) and sodium azide (0.176 g, 2.70 mmol) in DMF (2 mL) was heated at 80°C for 1 h using a microwave reactor. The crude reaction mixture was diluted with 95% ethanol (6 mL) and used directly in the next reaction step.

**N-(5-(Aminomethyl)quinolin-2-yl)acetamide (8H)**. The crude (DMF/Ethanol solution, 2 mL:6 mL) solution of N-(5-(azidomethyl)quinolin-2-yl)acetamide 8G was transferred to a 50 mL round bottom flask and palladium hydroxide on carbon (0.050 g, 0.36 mmol) was added. The reaction mixture was stirred under an atmosphere of hydrogen for 16 h. The resulting mixture was filtered through a phase separator to remove palladium residues and then concentrated under reduced pressure to give crude compound 8H (0.291 g, 100%) as a brown oil. The crude oil was used directly in the next reaction step without further purifications.

**5-(Aminomethyl)quinolin-2-amine (8I)**. A mixture of the crude N-(5-(aminomethyl)quinolin-2-yl)acetamide 8H (0.291 g, 1.35 mmol) was heated in 8 mL 20% NaOH at 120°C for 1 h. LCMS analysis showed full conversion of the acetamide to corresponding amino-quinoline and a pyridone by-product in an estimated 1:1 ratio. The mixture was concentrated under reduced pressure, re-dissolved in methanol and filtered. The crude product was purified by preparative HPLC, relevant fractions were pooled and freeze dried to give compound 8I as a white solid (0.028 g, 12%). Compound 8I was used directly without further purifications in the next reaction step.

**(S)-N-(1-{(2-Aminoquinolin-5-yl)methylamino}-1-oxo-3-phenylpropan-2-yl)-4-hydroxy-2-oxo-1,2-dihydroquinoline-6-carboxamide (8)**. To a solution of 5-(aminomethyl)quinolin-2-amine 8I (0.056 g, 0.330 mmol), 2-(1H-benzo[d][1,2,3]triazol-1-yl)-1,1,3,3-tetramethylisouronium tetrafluoroborate (0.118 g, 0.370 mmol) in DMF (2 mL) was added N,N-Diisopropylethylamine (DIPEA) (0.114 mL, 0.650 mmol) and (*S*)-2-(4-hydroxy-2-oxo-1,2-dihydroquinoline-6-carboxamido)-3-phenylpropanoic acid 15B. Resulting mixture was stirred at room temperature for 16 h. Excess DMF was removed under reduced pressure and the resulting residue was taken up in methanol and purified by preparative HPLC. Fractions containing the desired product was pooled and excess solvent removed by freeze drying to give compound 8 as a white solid (0.016 g, 10%). ^1^H NMR (400 MHz, CD_3_OD) δ 3.13 (2H, ddd, *J* = 7.3, 13.5, 39.8 Hz), 4.67 (2H, dd, *J* = 14.7, 41.8 Hz), 4.82 (1H, t, *J* = 7.4, 7.4 Hz), 6.77 (1H, d, *J* = 9.1 Hz), 7.03 (1H, d, *J* = 6.5 Hz), 7.18 (5H, s), 7.30 (1H, d, *J* = 8.6 Hz), 7.39–7.52 (8H, m), 7.89 (1H, dd, *J* = 2.1, 8.6 Hz), 8.00 (1H, d, *J* = 9.2 Hz), 8.39 (1H, d, *J* = 2.0 Hz). HRMS: calcd. for [M+H]^+^ C_29_H_25_N_5_O_4_ 508.1985, found 508.1988.

**N-(4-Chlorophenyl)-3-oxobutanamide (13A)**. A mixture of methyl 3-oxobutanoate (3.38 mL, 31.4 mmol), 4-chloroaniline (1.00 g, 7.84 mmol), DMAP (0.958 g, 7.84 mmol) and pyridine (4 mL) was refluxed in xylenes (20 mL) for 8 h. The reaction mixture was partitioned between 2M HCl (20 mL) and EtOAc (20 mL), the aqueous phase was extracted with EtOAc (2x10 mL) and the combined organic fractions was dried (Na_2_SO_4_) and then concentrated to produce compound 13A as a pale brown solid (1.330 g, 80%). ^1^H NMR (600 MHz, DMSO-d_6_) δ 2.20 (3H, s), 3.55 (2H, s), 7.36 (2H, d, *J* = 8.8 Hz), 7.60 (2H, d, *J* = 8.8 Hz), 10.22 (1H, s).

**4-Bromo-N-(4-chlorophenyl)-3-oxobutanamide (13B)**. To a stirred solution of N-(4-chlorophenyl)-3-oxobutanamide (13A) (18.2 g, 86.1 mmol) in AcOH (80 mL) at room temperature was dropwise added a solution of bromine (5.32 mL, 103 mmol) in AcOH (20 mL) over 3 h. Resulting mixture was stirred for 3 h, producing a brown precipitate. The reaction mixture was poured into a 1L beaker filled to 2/3 with crushed ice. A crude brown precipitate containing a mixture of various brominated species was collected by filtration. The product was purified by silica column chromatography (EtOAc/ DCM gradient from 0–5% EtOAc, with 0.5% TEA). Relevant fractions was pooled and concentrated under reduced pressure to give compound 13B as a pale brown solid (14.0 g, 56%). ^1^H NMR (400 MHz, DMSO-d_6_) δ 2.19 (2H, s), 3.53 (2H, s), 7.31–7.47 (2H, m), 7.54–7.61 (2H, m), 10.18 (1H, s).

**4-(Bromomethyl)-6-chloroquinolin-2(1H)-one (13C)**. 4-Bromo-N-(4-chlorophenyl)-3-oxobutanamide (13B) (12.0 g, 41.3 mmol) was heated at 45°C in sulfuric acid (60 mL) for 24 h. Due to slow conversion of the starting material another portion of sulfuric acid (60 mL) was added and the reaction was continued until complete conversion was observed (LCMS), a total of 46 h. The reaction was allowed to reach ambient temperature and then poured into a 2L beaker filled to 2/3 with crushed ice, producing a pink precipitate. The precipitate was collected by filtration, washed with ice-cold water and then dried on a freeze-drier giving compound 13C as a pink solid (10.2 g, 90%). ^1^H NMR (500 MHz, DMSO-d_6_) δ 4.93 (2H, s), 6.80 (1H, s), 7.35 (1H, d, *J* = 8.8 Hz), 7.59 (1H, dd, *J* = 2.2, 8.8 Hz), 7.89 (1H, d, *J* = 2.2 Hz), 11.95 (1H, s).

**Diethyl 2-{(6-chloro-2-oxo-1,2-dihydroquinolin-4-yl)methyl}malonate (13D)**. To a stirred solution of diethyl malonate (3.62 mL, 23.85 mmol) in THF (15 mL) at room temperature was added NaH (0.286 g, 11.93 mmol) in portions. Resulting mixture was stirred at room temperature for 10 min before a slurry of 4-(bromomethyl)-6-chloroquinolin-2(1H)-one (13C) (0.65 g, 2.39 mmol) in THF (15 mL) was added dropwise. The resulting brown suspension was refluxed for 1 h and then concentrated under reduced pressure to give a brown oil. Water was then added until a precipitate formed. The precipitate was filtered and washed with cold water and then dried on a freeze-drier, giving compound 13D as a white solid (0.688 g, 82%). ^1^H NMR (500 MHz, DMSO-d_6_) δ 1.12 (6H, t, *J* = 7.1, 7.1 Hz), 3.30 (2H, d, *J* = 8.0 Hz), 3.93 (1H, t, *J* = 8.0, 8.0 Hz), 4.10 (4H, q, *J* = 7.0, 7.0, 7.1 Hz), 6.38 (1H, s), 7.32 (1H, d, *J* = 8.8 Hz), 7.56 (1H, dd, *J* = 2.2, 8.8 Hz), 7.87 (1H, d, *J* = 2.2 Hz), 11.83 (1H, s).

**3-(6-Chloro-2-oxo-1,2-dihydroquinolin-4-yl)propanoic acid (24A)**. A suspension of diethyl 2-{(6-chloro-2-oxo-1,2-dihydroquinolin-4-yl)methyl}malonate (13D) (0.688 g, 1.96 mmol) in 20% HCl (aq) (30 mL) was refluxed for 12 h. The reaction was then cooled under stirring on an ice bath to form a precipitate. The precipitate was filtered, washed with cold water and dried on a freeze-drier to give compound 24A as a white solid (0.421 g, 86%). 1H NMR (500 MHz, DMSO-d6) δ 2.62 (2H, t, J = 7.4, 7.4 Hz), 3.03 (2H, t, J = 7.3, 7.3 Hz), 6.40 (1H, s), 7.32 (1H, d, J = 8.8 Hz), 7.55 (1H, dd, J = 2.2, 8.8 Hz), 7.80 (1H, d, J = 2.2 Hz), 11.77 (1H, s), 12.30 (1H, s).

**4-(Aminomethyl)-6-chloroquinolin-2(1H)-one (13E)**. A mixture of 4-(bromomethyl)-6-chloroquinolin-2(1H)-one (13C) (0.300 g, 1.10 mmol) and hexamethylene tetraamine (0.193 g, 1.38 mmol) in dichloroethane (20 mL) was refluxed for 5 h. A white precipitate formed and was collected by filtration. The intermediate solid was then refluxed in concentrated hydrochloric acid (10 mL) for 2 h. The reaction was stirred on an ice-bath and quenched with water, forming a new precipitate. The precipitate was filtered, washed with cold water and then dried on a freeze-drier to give compound 13E as a white solid (0,213 g, 93%). ^1^H NMR (500 MHz, DMSO-d_6_) δ 4.10 (2H, d, *J* = 0.9 Hz), 6.66 (1H, s), 6.97 (9H, s), 7.36 (1H, d, *J* = 8.8 Hz), 7.57 (1H, dd, *J* = 2.3, 8.8 Hz), 7.81 (1H, d, *J* = 2.3 Hz).

**(S)-N-(1-{(6-Chloro-2-oxo-1,2-dihydroquinolin-4-yl)methylamino}-1-oxo-3-phenylpropan-2-yl)-4-hydroxy-2-oxo-1,2-dihydroquinoline-6-carboxamide (13)**. A mixture of (*S*)-2-(4-hydroxy-2-oxo-1,2-dihydroquinoline-6-carboxamido)-3-phenylpropanoic acid (15B) (0.233 g, 0.660 mmol), T3P (0.788 mL, 1.32 mmol), 4-(aminomethyl)-6-chloroquinolin-2(1H)-one (13E) (0.138 g, 0.66 mmol) and TEA (0.277 mL, 1.99 mmol) was heated at 120°C for 20 min using a microwave reactor. Excess DMF was removed under reduced pressure to produce a crude brown oily residue, containing the desired product and an inseparable phosphorous ester of the product and the coupling reagent. The crude mixture was treated with 6M HCl at room temperature for 1h to cleave phosphorous ester byproducts. The reaction was concentrated and then purified by preparative HPLC to give compound 13 as a pale pink solid (0.109 g, 30%). ^1^H NMR (400 MHz, DMSO-d_6_) δ 2.97–3.08 (1H, m), 3.13 (1H, dd, *J* = 4.5, 13.7 Hz), 4.41–4.56 (2H, m), 4.67–4.76 (1H, m), 6.46 (1H, s), 6.72 (1H, s), 7.11 (1H, t, *J* = 7.2, 7.2 Hz), 7.19 (2H, t, *J* = 7.4, 7.4 Hz), 7.24–7.31 (3H, m), 7.34 (1H, d, *J* = 8.8 Hz), 7.47 (1H, s), 7.53 (1H, dd, *J* = 2.1, 8.8 Hz), 7.78 (1H, d, *J* = 2.1 Hz), 7.88 (1H, dd, *J* = 1.9, 8.6 Hz), 8.29 (1H, d, *J* = 1.8 Hz), 8.71 (1H, t, *J* = 5.7, 5.7 Hz), 8.77 (1H, d, *J* = 8.1 Hz). 2 protons were not observed. HRMS: calcd. for [M+H]^+^ C_29_H_23_ClN_4_O_5_ 543.1435, found 543.1447.

**(S)-Tert-butyl 1-{(6-chloro-2-oxo-1,2-dihydroquinolin-4-yl)methylamino}-1-oxo-3-phenylpropan-2-ylcarbamate (14A)**. 1H-Benzo[d][1,2,3]triazol-1-ol hydrate (0.468 g, 3.06 mmol) and N1-{(ethylimino)methylene}-N3,N3-dimethylpropane-1,3-diamine hydrochloride (0.693 g, 3.61 mmol) were added to a suspension of 4-(aminomethyl)-6-chloroquinolin-2(1H)-one (13E) (2.00 g, 2.78 mmol), (S)-2-(tert-butoxycarbonylamino)-3-phenylpropanoic acid (0.738 g, 2.78 mmol), and TEA (1.16 mL, 8.34 mmol) in DMF (15 mL). The mixture was stirred at room temperature for 16 h, diluted with ethanol (10 mL) and then filtered through a pad of Celite®. Excess solvent was removed under reduced pressure and the crude residue was purified by recrystallisation from EtOAc. Compound 14A was isolated as a white solid (0.965 g, 76%). 1H NMR (400 MHz, DMSO-d6) δ 1.30 (8H, s), 2.79 (1H, dd, J = 10.4, 13.5 Hz), 2.96 (1H, dd, J = 4.6, 13.7 Hz), 4.21 (1H, td, J = 4.6, 10.2, 10.3 Hz), 4.49 (2H, d, J = 5.5 Hz), 6.44 (1H, s), 7.08 (1H, d, J = 8.6 Hz), 7.18 (1H, dd, J = 2.6, 5.8 Hz), 7.24 (4H, dd, J = 5.4, 7.5 Hz), 7.33 (1H, d, J = 8.8 Hz), 7.55 (1H, dd, J = 2.2, 8.8 Hz), 7.84 (1H, t, J = 9.7, 9.7 Hz), 8.53 (1H, t, J = 5.8, 5.8 Hz), 11.71 (1H, s).

**(S)-2-Amino-N-{(6-chloro-2-oxo-1,2-dihydroquinolin-4-yl)methyl}-3-phenylpropanamide (14B)**. A solution of (*S*)-*tert*-butyl (1-[{(6-chloro-2-oxo-1,2-dihydroquinolin-4-yl)methyl}amino]-1-oxo-3-phenylpropan-2-yl)carbamate (14A) (0.086 g, 0.19 mmol) in DCM (8 mL) and TFA (3 mL) was stirred at room temperature for 16 h. Excess solvents was removed under reduced pressure to give compound 14B as a white solid (0.075 g, 100%). ^1^H NMR (600 MHz, DMSO-d_6_) δ 3.05 (2H, ddd, *J* = 7.1, 13.7, 32.2 Hz), 4.11 (1H, d, *J* = 4.5 Hz), 4.44–4.56 (2H, m), 6.43 (1H, s), 7.22 (5H, ddd, *J* = 6.7, 11.0, 14.2 Hz), 7.34 (1H, dd, *J* = 5.7, 17.9 Hz), 7.57 (1H, dd, *J* = 2.2, 8.8 Hz), 7.81 (1H, t, *J* = 9.2, 9.2 Hz), 8.32 (2H, s), 9.01 (1H, t, *J* = 5.8, 5.8 Hz), 11.84 (1H, s).

**(S)-2-Acetamido-N-{(6-chloro-2-oxo-1,2-dihydroquinolin-4-yl)methyl}-3-phenylpropanamide (14)**. Acetic acid (0.017 mL, 0.30 mmol) and 2-(1H-benzo[d][1,2,3]triazol-1-yl)-1,1,3,3-tetramethylisouronium tetrafluoroborate (0.095 g, 0.30 mmol) was dissolved in DMF (4 mL) and stirred at room temperature for 10 min. Pyridine (0.032 mL, 0.39 mmol) was added and the reaction mixture was stirred for another 10 min. (*S*)-2-amino-N-{(6-chloro-2-oxo-1,2-dihydroquinolin-4-yl)methyl}-3-phenylpropanamide (14B) (0.035 g, 0.10 mmol) was added and the resulting mixture was stirred at room temperature for 16 h. Excess DMF was removed under reduced pressure and resulting crude material was purified by preparative HPLC to give compound 14 as a white solid (0.023 g, 59%). ^1^H NMR (400 MHz, DMSO-d_6_) δ 1.78 (3H, s), 2.78 (1H, dd, *J* = 10.0, 13.5 Hz), 2.99 (1H, dd, *J* = 4.8, 13.6 Hz), 4.52 (3H, ddd, *J* = 5.2, 14.1, 23.7 Hz), 6.38 (1H, s), 7.16 (1H, dd, *J* = 4.3, 8.6 Hz), 7.23 (4H, d, *J* = 4.3 Hz), 7.33 (1H, d, *J* = 8.8 Hz), 7.55 (1H, dd, *J* = 1.9, 8.8 Hz), 7.82 (1H, d, *J* = 1.9 Hz), 8.26 (1H, d, *J* = 8.3 Hz), 8.62 (1H, t, *J* = 5.5, 5.5 Hz), 11.69 (1H, s). HRMS: calcd. for [M+H]^+^ C_21_H_20_ClN_3_O_3_ 398.1271, found 398.1270.

**4-Hydroxy-2-oxo-1,2-dihydroquinoline-6-carboxylic acid (15A)**. To a solution of methyl 4-aminobenzoate (2.34 g, 15.5 mmol) in DCM (40 mL) was dropwise added ethyl 3-chloro-3-oxopropanoate (2.58 mL, 20.1 mmol) and resulting mixture was stirred at room temperature for 16 h. The reaction mixture was washed with 1M NaOH (2 x 40 mL) and brine (40 mL). The organic layer was dried (Na_2_SO_4_), filtered and then concentrated under reduced pressure. The residue was hydrolyzed directly by stirring in a mixture of THF (10 mL), water (10 mL) and lithium hydroxide (1.85 g, 77.4 mmol) at room temperature for 16 h. Excess THF was removed under reduced pressure before the pH was adjusted to 3 with 2M HCl. The product precipitated and was collected by filtration. The solid material was then heated in polyphosphoric acid (5.24 mL, 15.5 mmol) at 120°C for 2 h. The reaction mixture was cooled on an ice bath and water (100 mL) was added under stirring. A white solid was formed and collected by filtration and washed with ice-cold water giving compound 15A as a white solid (2.30 g, 72%). ^1^H NMR (400 MHz, DMSO-d_6_) δ 5.77 (1H, s), 7.32 (1H, t, *J* = 13.8, 13.8 Hz), 7.96–8.07 (1H, m), 8.38 (1H, d, *J* = 1.8 Hz), 11.50 (1H, s), 11.61 (1H, s).

**(S)-2-(4-Hydroxy-2-oxo-1,2-dihydroquinoline-6-carboxamido)-3-phenylpropanoic acid (15B)**. A mixture of 4-hydroxy-2-oxo-1,2-dihydroquinoline-6-carboxylic acid (15A) (0.57 g, 2.78 mmol), 2-(1H-benzo[d][1,2,3]triazol-1-yl)-1,1,3,3-tetramethylisouronium tetrafluoroborate (1.07 g, 3.33 mmol), and N-ethyl-N-isopropylpropan-2-amine (1.94 mL, 11.1 mmol) in DMF (15 mL) was stirred at room temperature for 10 min, giving a yellow suspension. (*S*)-methyl 2-amino-3-phenylpropanoate hydrochloride (0.719 g, 3.33 mmol) was then added and resulting mixture stirred at room temperature for 16 h. The yellow mixture was partitioned between water and EtOAc, the aqueous layer was washed with DCM, dried (Na_2_SO_4_), filtered and concentrated under reduced pressure to give an orange oil. Ice-cold water was added to the crude oil forming an orange solid. The solid was washed with cold water and dried under reduced pressure to give the crude ester intermediate. The crude solid was hydrolyzed directly by stirring in a mixture of water, THF and lithium hydroxide for 2 h. Excess THF was removed under reduced pressure and 1M HCl was then added to the residue, forming a white precipitate. Compound 15B was collected by filtration (0.430 g, 44%). ^1^H NMR (500 MHz, DMSO-d_6_) δ 3.09 (1H, dd, *J* = 10.5, 13.8 Hz), 3.19 (1H, dd, *J* = 4.6, 13.8 Hz), 4.63 (1H, ddd, *J* = 4.6, 8.2, 10.6 Hz), 5.76 (1H, s), 7.17 (1H, t, *J* = 7.2, 7.2 Hz), 7.22–7.34 (5H, m), 7.90 (1H, dt, *J* = 7.3, 7.3, 14.6 Hz), 8.31 (1H, d, *J* = 1.9 Hz), 8.78 (1H, d, *J* = 8.1 Hz), 11.39 (1H, s), 11.54 (1H, s), 12.74 (1H, s).

**(S)-4-Hydroxy-N-(1-(methylamino)-1-oxo-3-phenylpropan-2-yl)-2-oxo-1,2-dihydroquinoline-6-carboxamide (15)**. A mixture of (*S*)-2-(4-hydroxy-2-oxo-1,2-dihydroquinoline-6-carboxamido)-3-phenylpropanoic acid (15B) (0.070 g, 0.20 mmol), O-(7-azabenzotriazol-1-yl)-N,N,N',N'-tetramethyluronium hexafluorophosphate (0.189 g, 0.50 mmol) was stirred in DMF (5 mL) at room temperature for 10 min before pyridine (0.321 mL, 3.97 mmol) and methylamine hydrochloride (0.268 g, 3.97 mmol) was added, resulting mixture was stirred at room temperature for 16 h. DMSO (5 mL) was added and excess DMF was then removed under reduced pressure. The remaining DMSO solution was purified by preparative HPLC to give compound 15 as a white solid (0.0683 g, 94%). ^1^H NMR (600 MHz, DMSO-d_6_) δ 2.60 (3H, t, *J* = 5.8, 5.8 Hz), 2.88–2.97 (2H, m), 3.04 (2H, dd, *J* = 3.2, 13.3 Hz), 4.49–4.55 (1H, m), 7.12–7.21 (2H, m), 7.25–7.31 (4H, m), 7.39 (1H, s), 7.41–7.47 (2H, m), 8.00 (1H, d, *J* = 4.7 Hz), 8.43 (1H, d, *J* = 8.4 Hz), 10.40 (1H, s). HRMS: calcd. for [M+H]^+^ C_20_H_19_N_3_O_4_ 366.1454, found 366.1462.

**(S)-N-(1-(Dimethylamino)-1-oxo-3-phenylpropan-2-yl)-4-hydroxy-2-oxo-1,2-dihydroquinoline-6-carboxamide (16)**. A suspension of 4-hydroxy-2-oxo-1,2-dihydroquinoline-6-carboxylic acid (15A) (0.050 g, 0.24 mmol) and O-(7-azabenzotriazol-1-yl)-N,N,N',N'-tetramethyluronium hexafluorophosphate (0.190 g, 0.50 mmol) was stirred in DMF (5 mL) at room temperature for 10 min before (S)-2-amino-N,N-dimethyl-3-phenylpropanamide hydrochloride (0.067 g, 0.29 mmol) and TEA (0.085 mL, 0.61 mmol) was added, resulting mixture was stirred at room temperature for 16 h. DMSO (5 mL) was added and excess DMF was then removed under reduced pressure. The remaining DMSO solution was purified by preparative HPLC to give compound 16 as a white solid (0.051 g, 55%). 1H NMR (500 MHz, DMSO-d6) δ 2.79 (3H, s), 2.94 (3H, s), 3.01 (2H, t, J = 7.5, 7.5 Hz), 5.07 (1H, d, J = 6.6 Hz), 5.73 (1H, s), 7.17 (1H, d, J = 7.3 Hz), 7.2–7.32 (5H, m), 7.93 (1H, d, J = 8.6 Hz), 8.34 (1H, s), 8.82 (1H, d, J = 8.0 Hz), 11.34 (1H, s), 11.43–11.78 (1H, m). HRMS: calcd. for [M+H]+ C21H21N3O4 380.1610, found 380.1601.

**2-(Trimethylsilyl)ethyl (6-chloro-2-oxo-1,2,3,4-tetrahydroquinolin-4-yl)methylcarbamate (18A)**. A mixture of 2-(6-chloro-2-oxo-1,2,3,4-tetrahydroquinolin-4-yl)acetic acid (20E) (0.577 g, 2.41 mmol), diphenyl phosphorazidate (0.623 mL, 2.89 mmol), TEA (0.671 mL, 4.82 mmol) in DMF (5 mL) was stirred at room temperature for 1 h before 2-(trimethylsilyl)ethanol (13.8 mL, 96.3 mmol) was added. The resulting mixture was heated at 100°C under microwave irradiation for 10 min. Excess solvent was removed under reduced pressure and the residual crude material was purified by preparative HPLC. Fractions containing the product were pooled and freeze-dried to give compound 18A as a colorless oil (0.628 g, 74%), that solidified over time. ^1^H NMR (500 MHz, CD_3_CN) δ -0.03–0.1 (9H, m), 0.88–1 (2H, m), 2.46 (1H, dd, *J* = 2.9, 16.6 Hz), 2.68 (1H, dd, *J* = 6.4, 16.6 Hz), 3.11 (1H, d, *J* = 4.5 Hz), 3.16–3.23 (2H, m), 4.04–4.13 (2H, m), 5.59 (1H, s), 6.83 (1H, t, *J* = 6.0, 6.0 Hz), 7.16–7.23 (2H, m), 8.34 (1H, s).

**4-(Aminomethyl)-6-chloro-3,4-dihydroquinolin-2(1H)-one (18B)**. To a solution of 2-(trimethylsilyl)ethyl ({6-chloro-2-oxo-1,2,3,4-tetrahydroquinolin-4-yl}methyl)carbamate (18A) (0.628 g, 1.77 mmol) in acetonitrile (25 mL) was added tetrabutylammonium fluoride (5.31 mL, 5.31 mmol). Resulting mixture was stirred at 60°C for 16 h. The conversion was slow and additional tetrabutylammonium fluoride (0.885 mL, 0.880 mmol) was added and the stirring continued at 60°C for 1 h. Excess solvents was removed under reduced pressure, the crude material was retaken in DMSO and purified by preparative HPLC. Fractions containing the product were pooled and freeze-dried to give compound 18B as a colorless oil. Tetrabutylammonium salt was still present in the NMR and additional purification was needed. The oil was dissolved in EtOAc/THF/MeOH and hydrochloric acid in dioxane (0.442 mL, 1.77 mmol) was added. Resulting mixture was stirred at room temperature for 16 h, forming a precipitate. The precipitate was collected by filtration and dried under reduced pressure to give compound 18B as a white solid (0.103 g, 24%). The material was used without further purifications. ^1^H NMR (400 MHz, CD_3_OD) δ 2.60 (1H, dd, *J* = 3.5, 16.8 Hz), 2.90 (1H, dd, *J* = 6.3, 16.8 Hz), 3.02–3.17 (2H, m), 3.2–3.27 (1H, m), 6.94 (1H, d, *J* = 8.4 Hz), 7.27–7.37 (2H, m). 3 protons were not observed.

**N-({2S}-1-({6-Chloro-2-oxo-1,2,3,4-tetrahydroquinolin-4-yl}methylamino)-1-oxo-3-phenylpropan-2-yl)-4-hydroxy-2-oxo-1,2-dihydroquinoline-6-carboxamide (18)**. A mixture of 4-(aminomethyl)-6-chloro-3,4-dihydroquinolin-2(1H)-one x HCl (18B) (0.055 g, 0.220 mmol), (S)-2-(4-hydroxy-2-oxo-1,2-dihydroquinoline-6-carboxamido)-3-phenylpropanoic acid (0.086 g, 0.240 mmol), 2-(1H-benzo[d][1,2,3]triazol-1-yl)-1,1,3,3-tetramethylisouronium tetrafluoroborate (0.107 g, 0.330 mmol), and pyridine (0.090 mL, 1.11 mmol) in DMF (4 mL) was stirred at room temperature for 16 h. LCMS analysis indicated the presence of starting material, additional 2-(1H-benzo[d][1,2,3]triazol-1-yl)-1,1,3,3-tetramethylisouronium tetrafluoroborate (0.036 g, 0.110 mmol) and pyridine (0.018 mL, 0.220 mmol) was added and resulting mixture was stirred for 10 h, achieving complete conversion of the starting material. Water (12 mL) was then added to the mixture forming a precipitation that was collected by filtration and then washed with ice cold water. The solid was dissolved in DMSO and purified by preparative HPLC. Fractions containing the product were collected and freeze dried to give compound 18 as a white solid. NMR analysis showed the presence of acetamide (singlet at 1.75 ppm in DMSO-d6) a byproduct from the basic buffer used in the preparative HPLC-system. The product was dissolved in 1M HCl and extracted with EtOAc. The combined organic fractions was dried (Na2SO4), filtered and concentrated under reduced pressure to give compound 18 as a white solid diasteriomeric mixture (0.045 g, 37%). 1H NMR (600 MHz, DMSO-d6) δ 2.37 (1H, ddd, J = 3.3, 16.3, 19.1 Hz), 2.59–2.67 (1H, m), 2.93–3.14 (4H, m), 3.20 (2H, ddd, J = 6.6, 13.4, 27.0 Hz), 4.67 (1H, s), 5.38 (1H, s), 6.87 (1H, dd, J = 2.6, 8.4 Hz), 7.09–7.35 (8H, m), 7.80 (1H, d, J = 7.0 Hz), 8.23 (1H, dd, J = 17.4, 39.9 Hz), 8.33–8.4 (1H, m), 8.53 (1H, d, J = 14.0 Hz), 10.25 (1H, d, J = 10.8 Hz), 10.63 (1H, d, J = 100.9 Hz). HRMS: calcd. for [M+H]+ C29H25ClN4O5 545.1592, found 545.1580.

**(S)-Tert-butyl 1-(4-(1H-tetrazol-5-yl)phenylamino)-1-oxo-3-phenylpropan-2-ylcarbamate (20A)**. A mixture of (S)-2-(tert-butoxycarbonylamino)-3-phenylpropanoic acid (3.95 g, 14.9 mmol), 2-(1H-benzo[d][1,2,3]triazol-1-yl)-1,1,3,3-tetramethylisouronium tetrafluoroborate (4.78 g, 14.9 mmol) and TEA (4.30 mL, 31.0 mmol) in DCM (50 mL) and DMF (20 mL) was stirred at room temperature for 30 min before 4-(1H-tetrazol-5-yl)aniline (2.00 g, 12.4 mmol) was added. Resulting slurry was stirred at room temperature for 16 h and then diluted with DMSO (5 mL). Excess solvent was removed under reduced pressure and the residual DMSO solution was purified by repeated runs on a preparative HPLC. Fractions containing the product were collected and concentrated under reduced pressure forming a precipitate in the remaining aqueous phase. The precipitate was collected by filtration and dried under reduced pressure to give compound 20A as a white solid (3.50 g, 69%). 1H NMR (500 MHz, DMSO-d6) δ 1.26 (9H, d, J = 45.6 Hz), 2.81–2.87 (1H, m), 2.97–3.01 (1H, m), 4.34 (1H, s), 7.16–7.32 (5H, m), 7.79 (2H, d, J = 8.8 Hz), 7.97 (2H, d, J = 8.7 Hz), 10.34 (1H, s). 2 protons are overlapping with the solvent peak.

**(S)-N-(4-(1H-Tetrazol-5-yl)phenyl)-2-amino-3-phenylpropanamide (20B)**. A suspension of (*S*)-*tert*-butyl 1-(4-(1H-tetrazol-5-yl)phenylamino)-1-oxo-3-phenylpropan-2-ylcarbamate (20A) (3.50 g, 8.57 mmol) in neat 2,2,2-trifluoroacetic acid (5.00 mL, 67.3 mmol) was stirred at room temperature for 1 h, producing a slight yellow solution. The solution was poured on crushed ice and stirred for 30 min to produce a white precipitate. The precipitate was collected by filtration and dried under reduced pressure to give compound 20B as a white trifluoroacetic acid salt (1.00 g, 28%). ^1^H NMR (500 MHz, DMSO-d_6_) δ 3.13 (2H, ddd, *J* = 7.1, 13.9, 39.8 Hz), 4.17 (1H, t, *J* = 6.9, 6.9 Hz), 7.29 (5H, dt, *J* = 7.5, 7.5, 11.9 Hz), 7.71 (2H, d, *J* = 8.7 Hz), 8.01 (2H, d, *J* = 8.7 Hz), 8.33 (3H, s), 10.64 (1H, s).

**Ethyl 3-(4-chloro-2-formylphenylamino)-3-oxopropanoate (20C)**. To a mixture of 2-amino-5-chlorobenzaldehyde (2.01 g, 12.9 mmol) and TEA (1.80 mL, 12.9 mmol) in DCM (40 mL), under an atmosphere of nitrogen at room temperature, was dropwise added ethyl 3-chloro-3-oxopropanoate (2.81 mL, 22.0 mmol) and the resulting orange-red solution was stirred for 1 h. Additional ethyl 3-chloro-3-oxopropanoate (0.496 mL, 3.88 mmol) was added and the reaction stirred for 1 h to drive the reaction to completion. The reaction mixture was washed with water, NaHCO_3_ and brine, dried (Na_2_SO_4_) and concentrated under reduced pressure to give compound 20C as a crude yellow solid (3.51 g, 100%). The material was used directly in the next reaction step, without further purifications.

**(E)-Methyl 3-(5-chloro-2-(3-ethoxy-3-oxopropanamido)phenyl)acrylate (20D)**. To a mixture Ethyl 3-(4-chloro-2-formylphenylamino)-3-oxopropanoate (20C) (3.46 g, 12.8 mmol) in toluene (40 mL) was slowly added methyl 2-(triphenylphosphoranylidene)acetate (5.58 g, 16.7 mmol) and the resulting mixture was refluxed for 2 h. Upon cooling to room temperature a precipitate formed. The precipitate was collected by filtration and dried under reduced pressure. The crude product was purified by silica gel column chromatography using a gradient from 20% to 50% of EtOAc in heptane as the eluent. Fractions containing the product were pooled together and the solvent was removed under reduced pressure to give compound 20D as a white solid (1.29 g, 31%). The material was used in the next reaction step without further purifications.

**2-(6-Chloro-2-oxo-1,2,3,4-tetrahydroquinolin-4-yl)acetic acid (20E)**. To a mixture of sodium methanolate (1.82 mL, 9.82 mmol) in MeOH at 0°C and under an atmosphere of nitrogen, (*E*)-methyl 3-(5-chloro-2-(3-ethoxy-3-oxopropanamido)phenyl)acrylate (20D) (1.28 g, 3.93 mmol) was slowly added and the resulting mixture was stirred for 1 h. The reaction was quenched with saturated NH_4_Cl and then extracted with DCM (3 x 100 mL). The combined organic fractions was washed (brine), dried (Na_2_SO_4_) and concentrated under reduced pressure to give 1.25 g of the di-ester as a slightly yellow oil that solidified upon standing. The crude di-ester was directly dissolved in a mixture of DMSO (9.2 mL), water (0.7 mL) and sodium chloride (0.351 g, 6.01 mmol) and then heated at 150°C for 16 h. The reaction was diluted with EtOAc and water, the phases separated and the water phase was extracted with EtOAc (3 x 100 mL). The combined organic fractions was washed with water and brine, dried (Na_2_SO_4_) and concentrated under reduced pressure. The crude mono-ester was hydrolyzed directly by stirring in 4M NaOH (10 mL) at room temperature for 4 h. The reaction mixture was washed with EtOAc, the organic fraction was discarded, and the aqueous phase was acidified with concentrated HCl. The acidic phase was extracted with EtOAc and the combined organic fractions was dried over Na_2_SO_4_, filtered and concentrated under reduced pressure to give compound 20E as a white solid (0.580 g, 61%). ^1^H NMR (400 MHz, DMSO-d_6_) δ 2.36 (1H, dd, *J* = 5.2, 16.3 Hz), 2.52–2.7 (3H, m), 3.34–3.42 (1H, m), 3.59 (3H, s), 6.83–6.92 (1H, m), 7.18–7.27 (2H, m), 10.27 (1H, s).

**N-(4-(1H-Tetrazol-5-yl)phenyl)-2-(2-(6-chloro-2-oxo-1,2,3,4-tetrahydroquinolin-4-yl)acetamido)-3-phenylpropanamide (20)**. A mixture of 2-(6-chloro-2-oxo-1,2,3,4-tetrahydroquinolin-4-yl)acetic acid (20E) (0.025 g, 0.100 mmol), TEA (0.036 mL, 0.260 mmol) and 2-(1H-benzo[d][1,2,3]triazol-1-yl)-1,1,3,3-tetramethylisouronium tetrafluoroborate (0.037 g, 0.110 mmol) in THF (3 mL) was stirred at room temperature for 10 min, before (*S*)-N-(4-(2H-tetrazol-5-yl)phenyl)-2-amino-3-phenylpropanamide 2,2,2-trifluoroacetate (20B) (0.044 g, 0.100 mmol) was added and the resulting mixture was stirred at room temperature for 16 h. The reaction was diluted with DMSO (1 mL) and excess solvent was removed under reduced pressure. The residual DMSO solution was purified by preparative HPLC to give compound 20 as a white solid diasteriomeric mixture (0.041 g, 75%). ^1^H NMR (600 MHz, DMSO-d_6_) δ 2.16–2.3 (2H, m), 2.37 (2H, d, *J* = 14.2 Hz), 2.82 (1H, s), 3.00 (2H, s), 4.67 (1H, s), 6.78–6.85 (1H, m), 6.98 (1H, s), 7.11–7.31 (7H, m), 7.76 (2H, d, *J* = 2.2 Hz), 7.94 (2H, s), 8.41 (1H, s), 10.18 (1H, s), 10.39 (1H, s). HRMS: calcd. for [M+H]^+^ C_27_H_24_ClN_7_O_3_ 530.1707, found 530.1704.

**6-Chloro-3,4-dihydroquinolin-2(1H)-one (21A)**. To a mixture of 4-chloro-2-iodoaniline (0.500 g, 1.97 mmol) in DMSO (10 mL) was added ethyl acrylate (0.643 mL, 5.92 mmol), tributyltin hydride (0.793 mL, 2.96 mmol) and (E)-2,2'-(diazene-1,2-diyl)bis(2-methylpropanenitrile) in toluene (1.97 mL, 0.39 mmol). The resulting mixture was heated at 100°C for 16 h. The reaction was diluted with DCM and washed with brine. The organic layer was dried (Na_2_SO_4_), filtered and concentrated under reduced pressure. The residue was filtered through a silica plug, using 40% EtOAc in Heptane as the eluent. Fractions containing the desired product was pooled and concentrated under reduced pressure to give compound 21A as a crude solid (0.210 g, 59%). The crude material was used in the next reaction step without further purifications.

**6-Chloro-1-(4-methoxybenzyl)-3,4-dihydroquinolin-2(1H)-one (21B)**. Sodium hydride in mineral oil (0.057 g, 1.09 mmol) was carefully added to a mixture of 6-chloro-3,4-dihydroquinolin-2(1H)-one (21A) (0.152 g, 0.84 mmol) in DMF (3 mL). After stirring at room temperature for 30 min, 1-(chloromethyl)-4-methoxybenzene (0.131 mL, 0.92 mmol) was added in one portion and resulting mixture stirred for 2 h at room temperature. The reaction mixture was partitioned between DCM and water and the organic layer was collected, dried (Na_2_SO_4_) and concentrated under reduced pressure. The crude residue containing compound 21B (0.200 g, 79%) was used directly in the next reaction step without further purifications.

**Tert-butyl 2-(6-chloro-1-(4-methoxybenzyl)-2-oxo-1,2,3,4-tetrahydroquinolin-3-yl)acetate (21C)**. To a solution of 6-chloro-1-(4-methoxybenzyl)-3,4-dihydroquinolin-2(1H)-one (21B) (0.200 g, 0.660 mmol) in dry THF (3 mL), at -78°C under an atmosphere of nitrogen, was dropwise added lithium diisopropylamide (0.552 mL, 0.990 mmol). After complete addition resulting mixture was stirred at -78°C for 10 min and then allowed to slowly reach room temperature. The reaction was again cooled to -78°C and *tert*-butyl 2-bromoacetate (0.150 mL, 0.93 mmol) was added by dropwise addition. Resulting mixture was stirred at -78°C for 1 h, followed by 1 h at 0°C. The reaction mixture was concentrated under reduced pressure, purified by silica column chromatography, using 0–100% EtOAc in heptane as the eluent. Fractions containing the desired product was pooled and concentrated under reduced pressure to give compound 21C as a pale yellow solid (0.071 g, 26%). ^1^H NMR (500 MHz, CDCl_3_) δ 1.48 (9H, s), 2.41 (1H, dt, *J* = 7.2, 7.2, 14.3 Hz), 2.81–3.01 (3H, m), 3.10 (1H, ddt, *J* = 5.6, 5.6, 7.8, 11.2 Hz), 3.77 (3H, s), 5.08 (2H, dd, *J* = 16.0, 110.8 Hz), 6.83 (3H, tt, *J* = 3.6, 3.6, 7.3, 7.3 Hz), 7.05–7.15 (4H, m).

**2-(6-Chloro-2-oxo-1,2,3,4-tetrahydroquinolin-3-yl)acetic acid (21D)**. A neat solution of *tert*-butyl 2-(6-chloro-1-(4-methoxybenzyl)-2-oxo-1,2,3,4-tetrahydroquinolin-3-yl)acetate (21C) (0.071 g, 0.170 mmol) in 2,2,2-trifluoroacetic acid (1 mL, 13.5 mmol) was heated to 80°C for 2 h. The reaction mixture was concentrated under reduced pressure to give compound 21D as a crude pale yellow solid (0.049 g, 98%). The crude material was used directly without further purifications in the next reaction step.

**N-(4-(1H-Tetrazol-5-yl)phenyl)-2-(2-(6-chloro-2-oxo-1,2,3,4-tetrahydroquinolin-3-yl)acetamido)-3-phenylpropanamide (21)**. A mixture of 2-(6-chloro-2-oxo-1,2,3,4-tetrahydroquinolin-3-yl)acetic acid (21D) (0.040 g, 0.17 mmol), 2-(1H-benzo[d][1,2,3]triazol-1-yl)-1,1,3,3-tetramethylisouronium tetrafluoroborate (0.070 g, 0.220 mmol) and TEA (0.069 mL, 0.500 mmol) in DMF (1 mL) was stirred for 10 min at room temperature before addition of (S)-N-(4-(1H-tetrazol-5-yl)phenyl)-2-amino-3-phenylpropanamide (20B) (0.085 g, 0.200 mmol). Resulting mixture was stirred at room temperature for 16 h. The reaction was diluted with DMSO (3 mL) and excess solvent was removed under reduced pressure. Resulting DMSO solutions was purified by preparative HPLC to give compound 21 as a white diasteriomeric mixture (0.034 g, 38%). 1H NMR (500 MHz, DMSO-d6) δ 2.20 (1H, ddd, J = 8.3, 14.9, 31.3 Hz), 2.55–2.68 (2H, m), 2.68–2.81 (2H, m), 2.82–2.92 (1H, m), 3.13 (1H, ddd, J = 4.7, 13.8, 34.1 Hz), 4.67–4.79 (1H, m), 6.84 (1H, dd, J = 4.3, 8.4 Hz), 7.07 (1H, d, J = 30.2 Hz), 7.13–7.35 (7H, m), 7.73 (2H, t, J = 8.1, 8.1 Hz), 7.95 (2H, d, J = 7.9 Hz), 10.19–10.31 (1H, m). The 1H NMR showed traces of acetamide (singlet at 1.75ppm in DMSO-d6) a byproduct from the basic buffer used in the preparative HPLC-systems. 2 protons were not observed. HRMS: calcd. for [M+H]+ C27H24ClN7O3 530.1707, found 530.171**1**.

**2-(6-Chloro-2-oxo-1,2-dihydroquinolin-3-yl)acetic acid (22A)**. A suspension of 4-chloro-2-iodoaniline (4.01 g, 15.8 mmol), dimethyl 2-methylenesuccinate (4.45 mL, 31.7 mmol), diacetoxypalladium (0.089 g, 0.400 mmol), triphenyl phosphine (0.208 g, 0.790 mmol) and sodium acetate (3.89 g, 47.5 mmol) in dry DMF (50 mL) was heated under an inert atmosphere at 110°C for 16 h. Excess solvent was removed under reduced pressure to give the crude ester as a brown solid. The ester was hydrolyzed directly by stirring at room temperature in 4M NaOH for 1 h. The reaction mixture was washed with EtOAc (4 x 100 mL) and then acidified to pH 1 with concentrated HCl, forming a precipitation. The precipitate was collected by filtration and washed with cold water and then dried under reduced pressure. The residual solid was retaken in THF and passed through a celite plug using EtOAc as the eluent. The filtered solution was dried (MgSO4) and concentrated under reduced pressure to give compound 22A (3.03 g, 81%) as a pale brown solid. 1H NMR (500 MHz, DMSO-d6) δ 3.09 (2H, s), 7.16 (1H, d, J = 8.7 Hz), 7.45 (1H, dd, J = 2.5, 8.7 Hz), 7.64 (1H, d, J = 2.4 Hz), 7.75 (1H, s), 10.45 (1H, s), 12.11–13.4 (1H, m).

**N-(4-(1H-Tetrazol-5-yl)phenyl)-2-(2-(6-chloro-2-oxo-1,2-dihydroquinolin-3-yl)acetamido)-3-phenylpropanamide (22)**. A mixture of 2-(6-chloro-2-oxo-1,2-dihydroquinolin-3-yl)acetic acid (22A) (0.020 g, 0.080 mmol), TEA (0.029 mL, 0.210 mmol) and 2-(1H-benzo[d][1,2,3]triazol-1-yl)-1,1,3,3-tetramethylisouronium tetrafluoroborate (0.030 g, 0.090 mmol) in THF (3 mL) was stirred at room temperature for 10 min before (*S*)-N-(4-(2H-tetrazol-5-yl)phenyl)-2-amino-3-phenylpropanamide 2,2,2-trifluoroacetate (20B) (0.036 g, 0.080 mmol) was added. The resulting mixture was stirred at room temperature for 12 h and then diluted with DMSO (3 mL). Excess solvents was removed under reduced pressure and the residual DMSO solution was purified by preparative HPLC to give compound 22 as a white solid (0.032 g, 72%). ^1^H NMR (500 MHz, DMSO-d_6_) δ 2.90 (1H, dd, *J* = 9.7, 13.8 Hz), 3.15 (1H, dd, *J* = 4.8, 13.8 Hz), 4.67 (1H, td, *J* = 4.9, 8.9, 9.0 Hz), 7.13–7.35 (6H, m), 7.50 (1H, dd, *J* = 2.3, 8.8 Hz), 7.64–7.7 (2H, m), 7.87 (2H, d, *J* = 8.6 Hz), 7.99 (2H, d, *J* = 8.6 Hz), 8.53 (1H, d, *J* = 8.0 Hz), 10.22 (1H, s), 12.01 (1H, s). 2 protons overlap with the water peak and 1 proton was not observed. HRMS: calcd. for [M+H]^+^ C_27_H_22_ClN_7_O_3_ 528.1551, found 528.1534.

**Ethyl 6-chloro-2-oxo-1,2-dihydroquinoline-3-carboxylate (23A)**. A mixture of 2-amino-5-chlorobenzaldehyde (5.00 g, 32.1 mmol), diethyl malonate (48.8 mL, 321 mmol) and piperidine (12.7 mL, 129 mmol) in EtOH (60 mL) was refluxed for 6 h. Upon cooling to room temperature a precipitation was formed. The precipitation was collected by filtration, washed with cold EtOH and dried under reduced pressure to give compound 23A (7.10 g, 88%) as a crude solid. The crude material was used without further purifications. ^1^H NMR (500 MHz, DMSO-d_6_) δ 1.29 (3H, t, *J* = 7.1, 7.1 Hz), 4.27 (2H, q, *J* = 7.1, 7.1, 7.1 Hz), 7.32 (1H, d, *J* = 8.8 Hz), 7.63 (1H, dd, *J* = 2.4, 8.8 Hz), 7.95 (1H, d, *J* = 2.3 Hz), 8.45 (1H, s), 12.16 (1H, s).

**6-Chloro-3-(hydroxymethyl)quinolin-2(1H)-one (23B)**. To a slurry of ethyl 6-chloro-2-oxo-1,2-dihydroquinoline-3-carboxylate (23A) (2.05 g, 8.15 mmol) Et_2_O (30 mL), under an atmosphere of nitrogen at 0°C, was dropwise added DIBAL-H (24.4 mL, 24.4 mmol). Resulting yellow-orange solution was allowed to slowly reach room temperature upon stirring over night. The reaction mixture was cooled to 0°C MeOH and 4M HCl was then carefully added. Resulting mixture was extracted with EtOAc (3 x 100 mL), the combined organic fractions was washed with brine, dried (Na_2_SO_4_), filtered and concentrated under reduced pressure to give compound 23B as a pale brown oil (0.861 g, 50%). The crude material contained traces of byproducts with a reduced ring system, but was used without further purifications. ^1^H NMR (400 MHz, DMSO-d_6_) δ 4.40 (2H, dd, *J* = 1.6, 5.4 Hz), 5.28 (1H, t, *J* = 5.4, 5.4 Hz), 7.31 (1H, d, *J* = 8.8 Hz), 7.48 (1H, dd, *J* = 2.4, 8.8 Hz), 7.78–7.93 (2H, m), 11.91 (1H, s).

**6-Chloro-3-(chloromethyl)quinolin-2(1H)-one (23C)**. A neat mixture of 6-chloro-3-(hydroxymethyl)quinolin-2(1H)-one (23B) (0.400 g, 1.91 mmol) and SOCl_2_ (10 mL) was refluxed for 6 h. Excess SOCl_2_ was removed under reduced pressure and the residue carefully quenched with saturated NaHCO_3_. The mixture was diluted with EtOAc and the layers were separated. The aqueous phase was extracted with EtOAc (3 x 20 mL). The combined organic fractions was dried (Na_2_SO_4_), filtered and concentrated under reduced pressure to give compound 23C as a crude pale yellow solid (0.460 g, 100%). The crude material was used directly in the next reaction step without further purifications.

**3-(6-Chloro-2-oxo-1,2-dihydroquinolin-3-yl)propanoic acid (23D)**. A mixture of diethyl malonate (1.99 mL, 13.0 mmol), sodium hydride (0.416 g, 10.4 mmol) in THF (15 mL) was stirred at room temperature under an atmosphere of nitrogen for 10 min. A slurry of 6-chloro-3-(chloromethyl)quinolin-2(1H)-one (23C) (0.474 g, 2.08 mmol) in THF (15 mL) was dropwise added to the reaction mixture and the resulting pale yellow suspension was refluxed for 2 h. Excess solvent was removed under reduced pressure and the residue poured on crushed ice, forming a white precipitate. The precipitate was collected by filtration, washed with cold water and decarboxylated immediately by adding concentrated HCl (20 mL) and refluxing the resulting suspension for 16 h. The yellow solution was allowed to reach room temperature and then stirred on an ice bath to form a new precipitate. The precipitate was filtered, washed with cold water and dried under reduced pressure to give compound 23D as a pale yellow solid (0.313 g, 60%). ^1^H NMR (500 MHz, DMSO-d_6_) δ 2.55 (2H, t, *J* = 7.5, 7.5 Hz), 2.71 (2H, t, *J* = 7.5, 7.5 Hz), 7.30 (1H, t, *J* = 9.8, 9.8 Hz), 7.43–7.52 (1H, m), 7.73 (2H, dd, *J* = 4.9, 9.9 Hz), 11.90 (1H, s). 1 proton was not observed.

**(S)-N-(4-(1H-Tetrazol-5-yl)phenyl)-2-(3-(6-chloro-2-oxo-1,2-dihydroquinolin-3-yl)propanamido)-3-phenylpropanamide (23)**. A mixture of 3-(6-chloro-2-oxo-1,2-dihydroquinolin-3-yl)propanoic acid (23D) (0.043 g, 0.170 mmol), 2-(1H-benzo[d][1,2,3]triazol-1-yl)-1,1,3,3-tetramethylisouronium tetrafluoroborate (0.066 g, 0.210 mmol), and N-ethyl-N-isopropylpropan-2-amine (0.119 mL, 0.680 mmol) dissolved in DMF (2 mL) was stirred at room temperature for 10 min before (*S*)-N-(4-(1H-tetrazol-5-yl)phenyl)-2-amino-3-phenylpropanamide 2,2,2-trifluoroacetate (20B) (0.072 g, 0.170 mmol) was added. Resulting mixture was stirred at room temperature for 16 h and then diluted with DMSO (5 mL). Excess solvent was removed under reduced pressure and the residual DMSO solution was purified by preparative HPLC. Fractions containing the product was pooled and freeze-dried to give compound 23 as a white solid (0.048 g, 52%). ^1^H NMR (500 MHz, DMSO-d_6_) δ 2.32–2.44 (2H, m), 2.64 (2H, d, *J* = 7.3 Hz), 2.85 (1H, d, *J* = 13.7 Hz), 3.03 (1H, dd, *J* = 4.9, 13.8 Hz), 4.67 (1H, s), 7.21 (6H, ddt, *J* = 7.2, 7.2, 38.4, 46.0 Hz), 7.44 (1H, dd, *J* = 2.3, 8.8 Hz), 7.53–7.62 (2H, m), 7.70 (2H, d, *J* = 8.6 Hz), 7.93 (2H, d, *J* = 8.6 Hz), 8.39 (1H, d, *J* = 8.0 Hz), 10.30 (1H, s), 11.84 (1H, s). 1 proton was not observed. HRMS: calcd. for [M+H]^+^ C_28_H_24_ClN_7_O_3_ 542.1707, found 542.1732.

**(S)-N-(4-(1H-Tetrazol-5-yl)phenyl)-2-(3-(6-chloro-2-oxo-1,2-dihydroquinolin-4-yl)propanamido)-3-phenylpropanamide (24)**. A mixture of 3-(6-chloro-2-oxo-1,2-dihydroquinolin-4-yl)propanoic acid (24A) (0.080 g, 0.320 mmol), 2-(1H-benzo[d][1,2,3]triazol-1-yl)-1,1,3,3-tetramethylisouronium tetrafluoroborate (0.122 g, 0.380 mmol) and TEA (0.133 mL, 0.950 mmol) in DMF (4 mL) was stirred at room temperature for 10 min before (*S*)-N-(4-(1H-tetrazol-5-yl)phenyl)-2-amino-3-phenylpropanamide (20B) (0.108 g, 0.350 mmol) was added. Resulting mixture was stirred at room temperature for 16 h and then diluted with DMSO (5 mL). Excess solvent was removed under reduced pressure and the residual DMSO solution was purified by preparative HPLC. Fractions containing the product was pooled and freeze-dried to give compound 24 as a white solid (0.064 g, 37%). ^1^H NMR (600 MHz, DMSO-d_6_) δ 2.83 (1H, dd, *J* = 9.3, 13.8 Hz), 2.89 (2H, t, *J* = 7.5, 7.5 Hz), 3.01 (1H, dd, *J* = 5.4, 13.8 Hz), 4.70 (1H, d, *J* = 5.2 Hz), 6.33 (1H, s), 7.15 (1H, d, *J* = 6.6 Hz), 7.19–7.25 (4H, m), 7.29 (1H, d, *J* = 8.8 Hz), 7.51 (1H, dd, *J* = 2.3, 8.8 Hz), 7.70 (1H, d, *J* = 2.2 Hz), 7.75 (2H, d, *J* = 8.7 Hz), 7.94 (2H, d, *J* = 8.7 Hz), 8.41 (1H, d, *J* = 8.1 Hz), 10.39 (1H, s), 11.72 (1H, s). 2 protons overlap with the solvent peak and 1 proton was not observed. HRMS: calcd. for [M+H]^+^ C_28_H_24_ClN_7_O_3_ 542.1707, found 542.1701.

**6-Chloro-4-({methylamino}methyl)quinolin-2(1H)-one (25A)**. A mixture of 4-(bromomethyl)-6-chloroquinolin-2(1H)-one (13C) (0.300 g, 1.10 mmol), 2M methanamine in THF (6 mL, 12 mmol) and TEA (0.460 mL, 3.30 mmol) was stirred at 50°C for 1 h. A solid precipitate formed upon cooling to room temperature, the precipitate was collected by filtration and washed with cold THF. The crude solid of compound 25A (0.0930 g, 38%) was used directly in the next reaction step without further purifications.

**(S)-Tert-butyl 1-[{(6-chloro-2-oxo-1,2-dihydroquinolin-4-yl)methyl}(methyl)amino]-1-oxo-3-phenylpropan-2-ylcarbamate (25B)**. A mixture of 6-chloro-4-({methylamino}methyl)quinolin-2(1H)-one (25A) (0.200 g, 0.900 mmol), (*S*)-2-(*tert*-butoxycarbonylamino)-3-phenylpropanoic acid (0.262 g, 0.990 mmol), 1H-benzo[d][1,2,3]triazol-1-ol (0.167 g, 0.990 mmol), N1-({ethylimino}methylene)-N3,N3-dimethylpropane-1,3-diamine hydrochloride (0.224 g, 1.17 mmol) and N-ethyl-N-isopropylpropan-2-amine (0.469 mL, 2.69 mmol) in DMF (5 mL) was stirred at room temperature for 12 h. Excess DMF was removed under reduced pressure, the crude product was retaken in DCM and washed with saturated NaHCO_3_ and brine. The organic layer was dried (Na_2_SO_4_), filtered and then concentrated under reduced pressure to give compound 25B as a crude solid (0.300 g, 71%). The crude product was used in the next reaction step without further purifications.

**(S)-2-Amino-N-({6-chloro-2-oxo-1,2-dihydroquinolin-4-yl}methyl)-N-methyl-3-phenylpropanamide (25C)**. A solution of (S)-tert-butyl 1-[{(6-chloro-2-oxo-1,2-dihydroquinolin-4-yl)methyl}(methyl)amino]-1-oxo-3-phenylpropan-2-ylcarbamate (25B) (0.300 g, 0.640 mmol) in excess of 4M HCl in dioxane (6 mL) was stirred at room temperature for 2 h. LCMS indicated full conversion to the desired product. The solvent was evaporated to give compound 25C as a crude solid. The crude product used without further purifications in the next reaction step.

**(S)-N-(1-[{(6-Chloro-2-oxo-1,2-dihydroquinolin-4-yl)methyl}(methyl)amino]-1-oxo-3-phenylpropan-2-yl)-4-hydroxy-2-oxo-1,2-dihydroquinoline-6-carboxamide (25)**. A mixture of (*S*)-2-amino-N-{(6-chloro-2-oxo-1,2-dihydroquinolin-4-yl)methyl}-N-methyl-3-phenylpropanamide (25C) (0.240 g, 0.650 mmol), 4-hydroxy-2-oxo-1,2-dihydroquinoline-6-carboxylic acid (15A) (0.133 g, 0.650 mmol), 1H-benzo[d][1,2,3]triazol-1-ol (0.110 g, 0.710 mmol), N1-{(ethylimino)methylene}-N3,N3-dimethylpropane-1,3-diamine hydrochloride (0.162 g, 0.840 mmol) and N-ethyl-N-isopropylpropan-2-amine (0.339 mL, 1.95 mmol) in DMF (2 mL) was stirred at room temperature for 12 h and then diluted with DMSO (3 mL). Excess solvent was removed under reduced pressure and the residual DMSO solution was purified by preparative HPLC. Fractions containing the product were pooled and freeze-dried to give compound 25 as a white solid (0.011 g, 3%). ^1^H NMR (600 MHz, DMSO-d_6_) δ 2.83 (1H, d, *J* = 95.6 Hz), 2.99 (3H, d, *J* = 51.9 Hz), 3.11 (2H, qd, *J* = 8.8, 13.7, 14.1, 14.1 Hz), 4.65–5.23 (3H, m), 5.73 (1H, d, *J* = 27.8 Hz), 6.22 (1H, d, *J* = 160.4 Hz), 7.05–7.3 (5H, m), 7.36 (2H, dd, *J* = 8.0, 14.6 Hz), 7.57 (1H, tdd, *J* = 2.0, 8.7, 25.5, 25.5 Hz), 7.76 (1H, dd, *J* = 2.1, 86.8 Hz), 7.94–8.05 (1H, m), 8.34 (1H, t, *J* = 65.4, 65.4 Hz), 8.97 (1H, dd, *J* = 8.0, 81.4 Hz), 11.33 (1H, d, *J* = 51.0 Hz), 11.72 (1H, d, *J* = 141.3 Hz). The complexity of the ^1^H proton NMR was a result of rotamers. HRMS: calcd. for [M+H]^+^ C_30_H_25_ClN_4_O_5_ 557.1592, found 557.1571.

**(S)-tert-butyl 1-[{(6-chloro-2-oxo-1,2-dihydroquinolin-4-yl)methyl}(methyl)amino]-1-oxo-3-phenylpropan-2-yl(methyl)carbamate (26A)**. A mixture of 6-chloro-4-({methylamino}methyl)quinolin-2(1H)-one (25A) (0.270 g, 1.21 mmol), (*S*)-2-(*tert*-butoxycarbonyl(methyl)amino)-3-phenylpropanoic acid (0.373 g, 1.33 mmol), 1H-benzo[d][1,2,3]triazol-1-ol (0.205 g, 1.33 mmol), N1-({ethylimino}methylene)-N3,N3-dimethylpropane-1,3-diamine hydrochloride (0.302 g, 1.58 mmol) and N-ethyl-N-isopropylpropan-2-amine (0.634 mL, 3.64 mmol) in DMF (5 mL) was stirred at room temperature for 16 h. The reaction was diluted with DCM (20 mL) and then washed with saturated NaHCO3 (2 x 20 mL), water (20 mL) and brine (20 mL). The organic layer was dried using a phase separator and then concentrated under reduced pressure to give compound 26A as a crude solid (0.401 g, 68%). The crude material was used without further purifications in the next reaction step.

**(S)-N-({6-chloro-2-oxo-1,2-dihydroquinolin-4-yl}methyl)-N-methyl-2-(methylamino)-3-phenylpropanamide (26B)**. A solution of (*S*)-*tert*-butyl 1-[{(6-chloro-2-oxo-1,2-dihydroquinolin-4-yl}methyl](methyl)amino)-1-oxo-3-phenylpropan-2-yl(methyl)carbamate (26A) (0.401 g, 0.830 mmol) in excess of 4M hydrogen chloride in dioxane (6 mL) was stirred at room temperature for 3 h. The reaction was concentrated under reduced pressure to give the crude compound 26B (0.300 g 95%). The crude material was used without further purifications in the next reaction step.

**(S)-N-(1-[{(6-Chloro-2-oxo-1,2-dihydroquinolin-4-yl)methyl}(methyl)amino]-1-oxo-3-phenylpropan-2-yl)-4-hydroxy-N-methyl-2-oxo-1,2-dihydroquinoline-6-carboxamide (26)**. A mixture of (*S*)-N-{(6-chloro-2-oxo-1,2-dihydroquinolin-4-yl)methyl}-N-methyl-2-(methylamino)-3-phenylpropanamide (26B) (0.200 g, 0.520 mmol), 4-hydroxy-2-oxo-1,2-dihydroquinoline-6-carboxylic acid (15A) (0.107 g, 0.520 mmol), 1H-benzo[d][1,2,3]triazol-1-ol (0.088 g, 0.570 mmol), N1-{(ethylimino)methylene}-N3,N3-dimethylpropane-1,3-diamine hydrochloride (0.130 g, 0.680 mmol) and N-ethyl-N-isopropylpropan-2-amine (0.272 mL, 1.56 mmol) in DMF (3 mL) was stirred at room temperature for 48 h and then diluted with DMSO (3 mL). Excess solvent was removed under reduced pressure and the residual DMSO solution was purified by preparative HPLC. Fractions containing the product were pooled and freeze-dried to give compound 26 as a white solid (0.016 g, 5%). ^1^H NMR (500 MHz, DMSO-d_6_) δ 2.82 (3H, d, *J* = 84.1 Hz), 3.00 (3H, d, *J* = 28.6 Hz), 3.18 (2H, s), 4.11 (1H, s), 4.75 (2H, dd, *J* = 17.7, 49.7 Hz), 5.65 (1H, d, *J* = 20.7 Hz), 5.87 (1H, d, *J* = 7.5 Hz), 6.37 (1H, s), 6.94 (1H, d, *J* = 8.3 Hz), 7.17–7.37 (8H, m), 7.58 (1H, dd, *J* = 2.2, 8.8 Hz), 7.77 (1H, d, *J* = 56.1 Hz), 11.16 (1H, d, *J* = 28.3 Hz), 11.87 (1H, s). The complexity of the ^1^H proton NMR was a result of rotamers. HRMS: calcd. for [M+H]^+^ C_31_H_27_ClN_4_O_5_ 571.1748, found 571.1756.

**N-{(6-Chloro-2-oxo-1,2-dihydroquinolin-4-yl)methyl}-N,4-dimethyl-3-phenyl-1H-pyrazole-5-carboxamide (28)**. A mixture of 6-chloro-4-{(methylamino)methyl}quinolin-2(1H)-one (25A) (0.270 g, 1.21 mmol), 4-methyl-3-phenyl-1H-pyrazole-5-carboxylic acid (0.319 g, 1.58 mmol), 1H-benzo[d][1,2,3]triazol-1-ol (0.225 g, 1.33 mmol), N1-{(ethylimino)methylene}-N3,N3-dimethylpropane-1,3-diamine hydrochloride (0.302 g, 1.58 mmol), and N-ethyl-N-isopropylpropan-2-amine (0.634 mL, 3.64 mmol) in DMF (5 mL) was stirred at room temperature for 16 h and then diluted with DMSO (3 mL). Excess solvent was removed under reduced pressure and the residual DMSO solution was purified by preparative HPLC. Fractions containing the product were pooled and freeze-dried to give compound 28 as a white solid (0.060 g, 12%). ^1^H NMR (500 MHz, DMSO-d_6_) δ 2.23 (3H, d, *J* = 21.8 Hz), 3.09 (3H, d, *J* = 45.8 Hz), 5.04 (2H, d, *J* = 109.3 Hz), 6.47 (1H, dd, *J* = 44.1, 92.5 Hz), 7.35–7.6 (7H, m), 7.71–7.95 (1H, m), 11.8–11.96 (1H, m), 13.30 (1H, d, *J* = 51.7 Hz). The complexity of the ^1^H proton NMR was a result of rotamers. HRMS: calcd. for [M+H]^+^ C_22_H_19_ClN_4_O_2_ 407.1275, found 407.1288.

## Supporting Information

S1 File Additional chemistry experimental details and NMR spectra. (DOCX)
