# Supplementary material for: Creating Novel Activated Factor XI Inhibitors through Fragment Based Lead Generation and Structure Aided Drug Design
Source: PLoS One. 2015 Jan 28;10(1):e0113705. doi: 10.1371/journal.pone.0113705 (PMC4309560; doi:10.1371/journal.pone.0113705)

**Supporting Information**

**Contents:**

1. Additional chemistry materials and methods information S2
2. Scheme S1. Synthesis of compound **4** S3
3. NMR spectra of manuscript compounds S4-S19
4. **Additional chemistry materials and methods information**

Chemicals and solvents from commercially available sources were purchased and used without further purification. High-resolution mass spectrometry (HRMS) analysis was performed using a Waters XEVO-qTOF instrument (Waters, Milford, USA) with MassLynx 4.1 software. If necessary, the purity was determined by ultra high performance liquid chromatography (U(H)PLC). Purity of all final compounds was 95% or higher at 210 nm. The instrument was a Waters U(H)PLC Acquity system (Binary pump, Sample Manager, Column Compartment and UV-PDA detector). The column was a Waters Acquity BEH C18, 1.7 μm particle size (100 mm × 2.1 mm).

Compounds **1**, **2**, **9**, **10**, **11** and **27** were obtained from the AstraZeneca corporate compound collection, compounds **3**, **5**, **6**, **7**, **12**, **17**, and **19** were commercially available and compounds **9**^30^, **10**^30^ and **11**^29^ were synthesized according to literature. Experimental and spectroscopic details for all other non-commercially available compounds are reported in the experimental section and their ^1^H NMR spectras can be found below.

Preparative HPLC was performed on various Waters systems using Kromasil C8 columns (10 μm 250x20 ID mm or 10 μm 250x50 ID mm), with a suitable gradient of acetonitrile in H_2_O/acetonitrile/formic acid over 20-30 minutes and a flow of 19 or 100 mL/min respectively. The compounds were detected by UV at 236, 240, 248, 261, or 270 nm.

1. **Scheme S1. Synthesis of compound 4**

**N-(3-(2-(2-aminoethoxy)ethoxy)phenyl)-6-carbamimidoyl-4-(pyrimidin-2-ylamino)-2-naphthamide (4)** was synthesized in an 8 step sequence from methyl 6-cyano-2-naphthoate according to the sequence described in **Scheme S1**.

**Scheme S1.**

1. **NMR spectra of manuscript compounds**


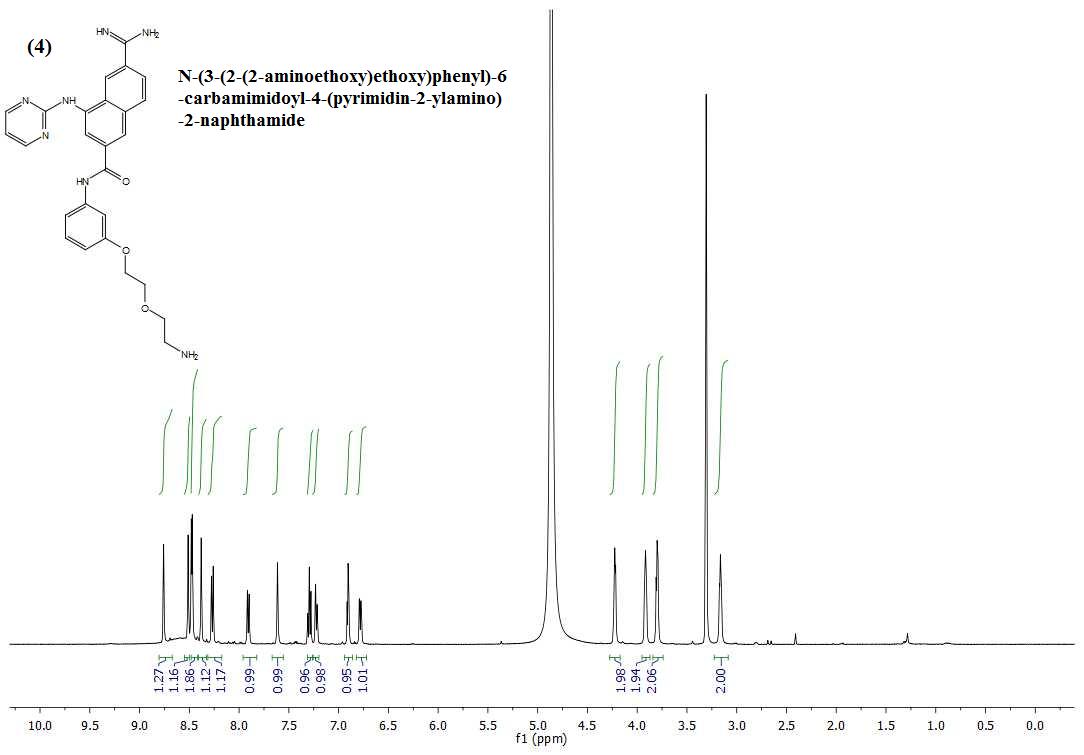


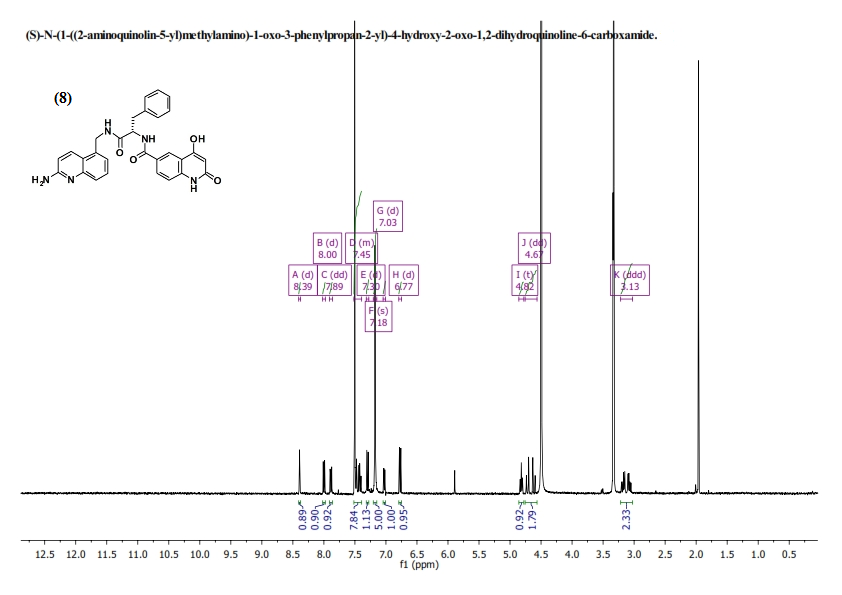


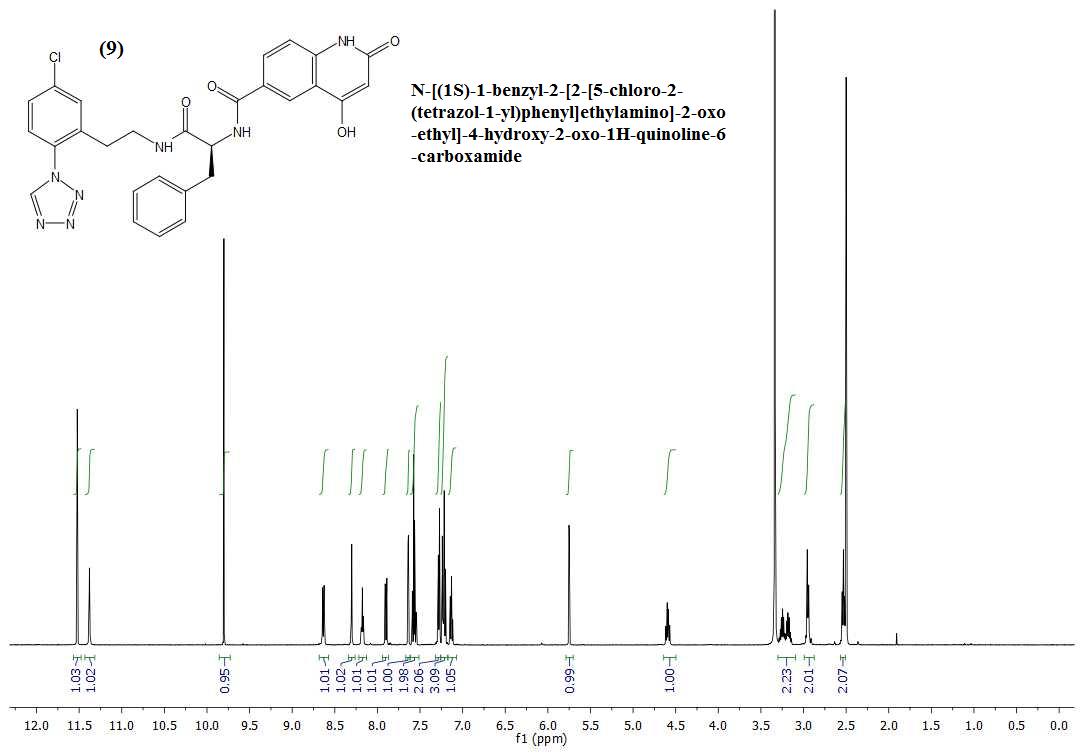


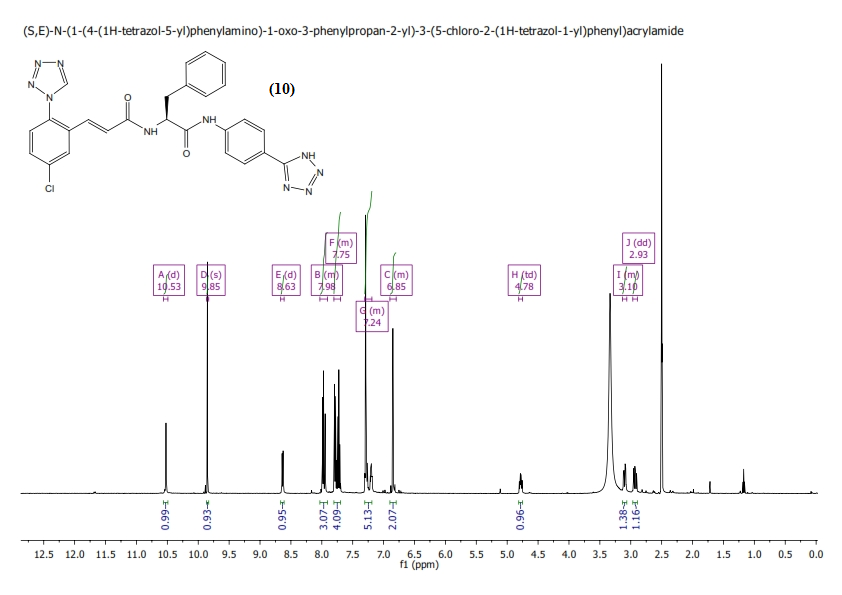


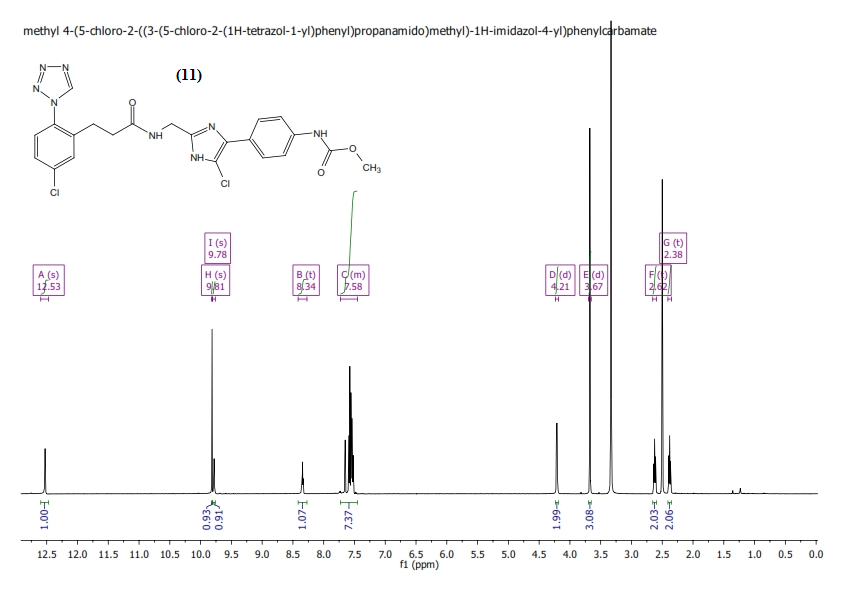


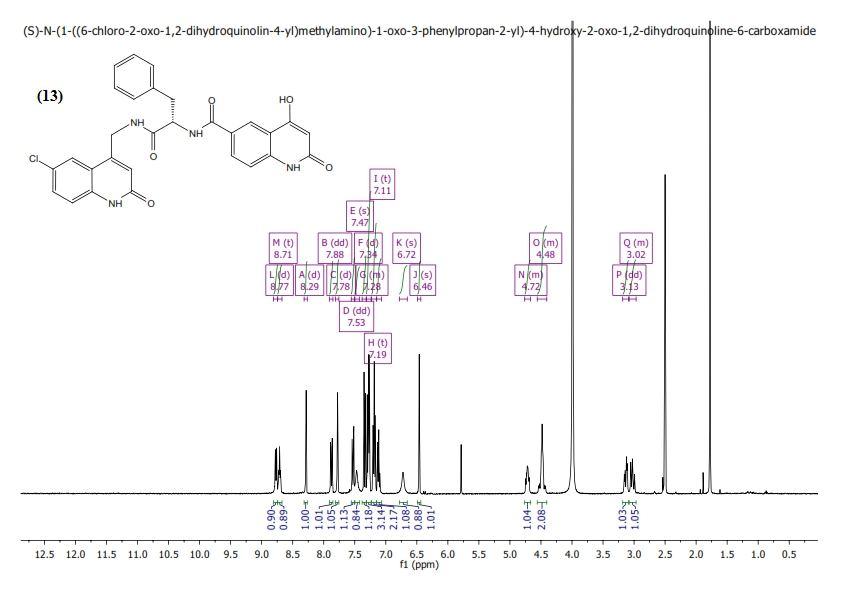


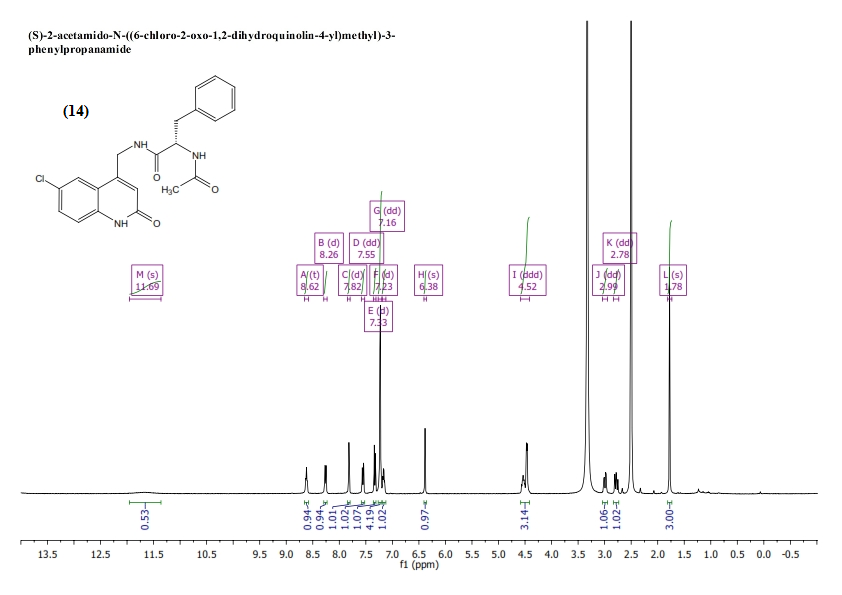


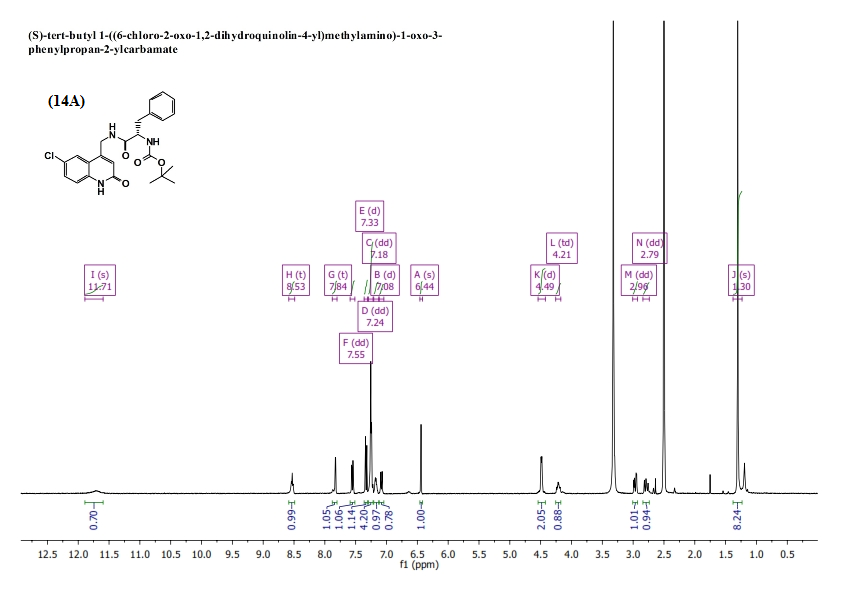


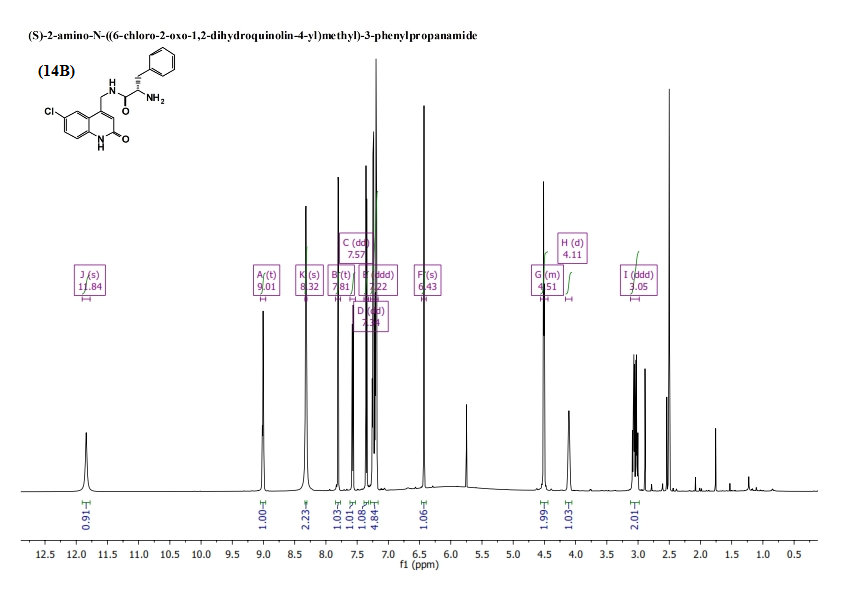


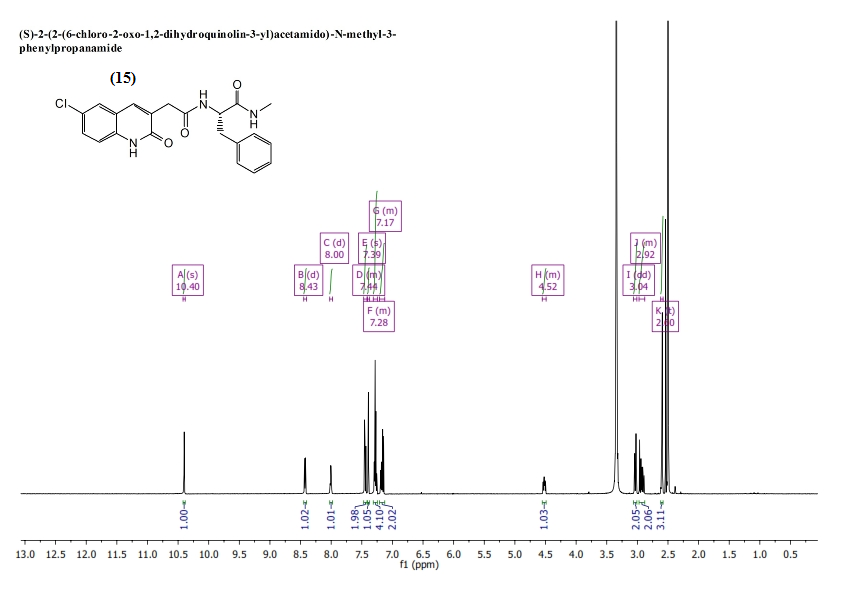


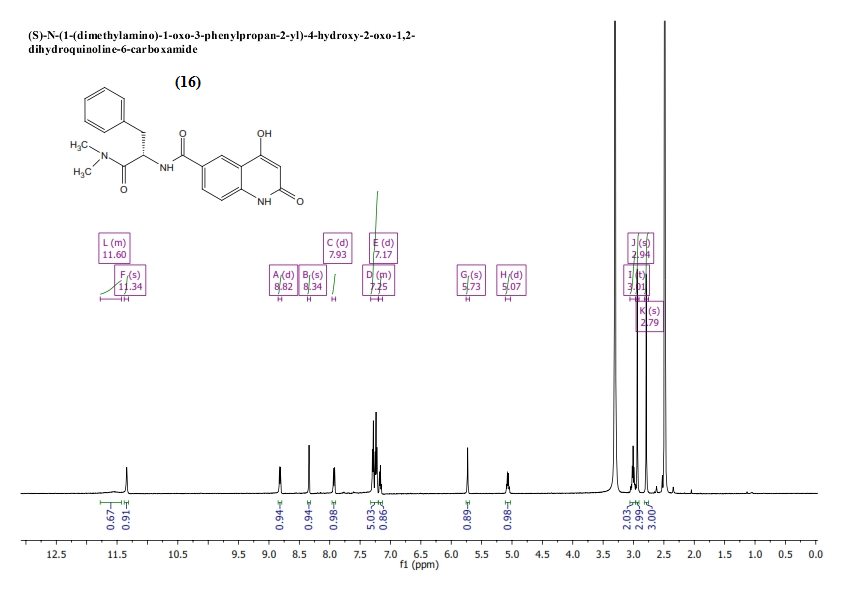


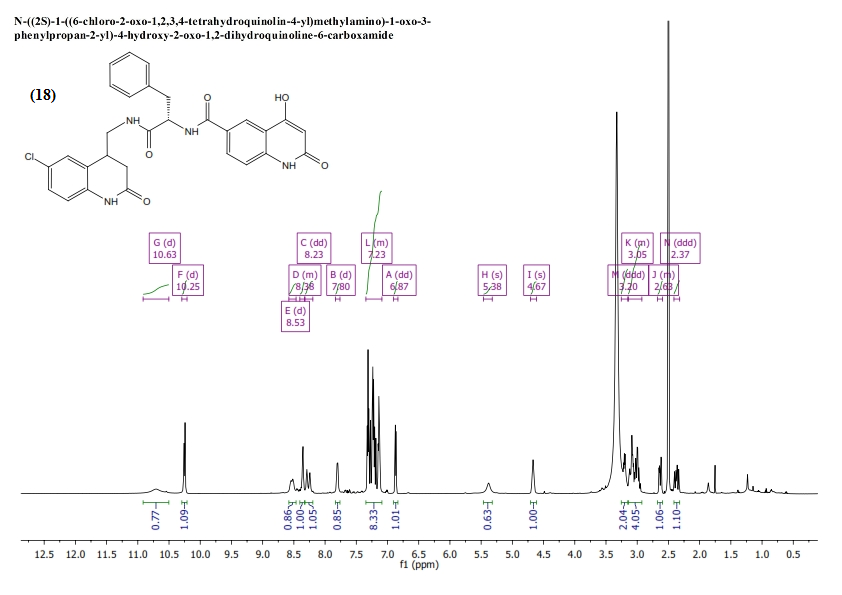


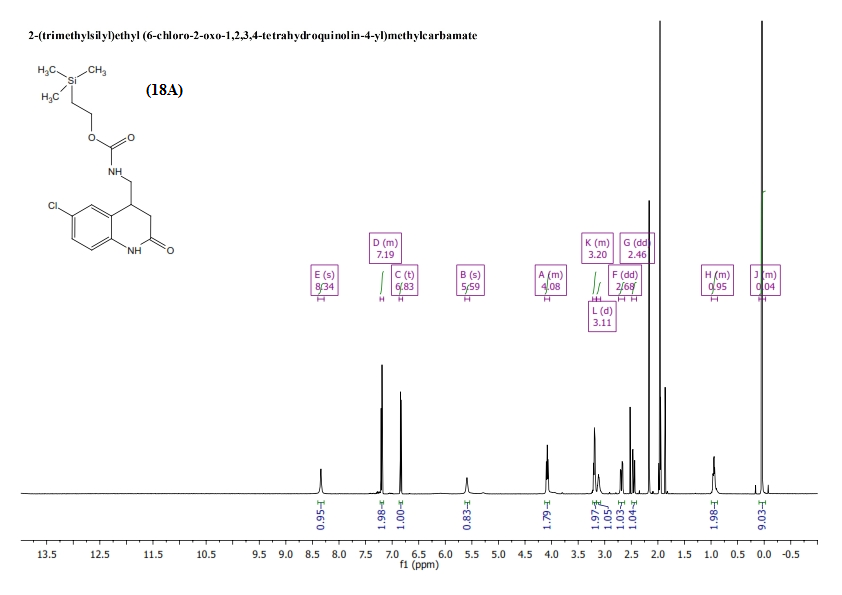


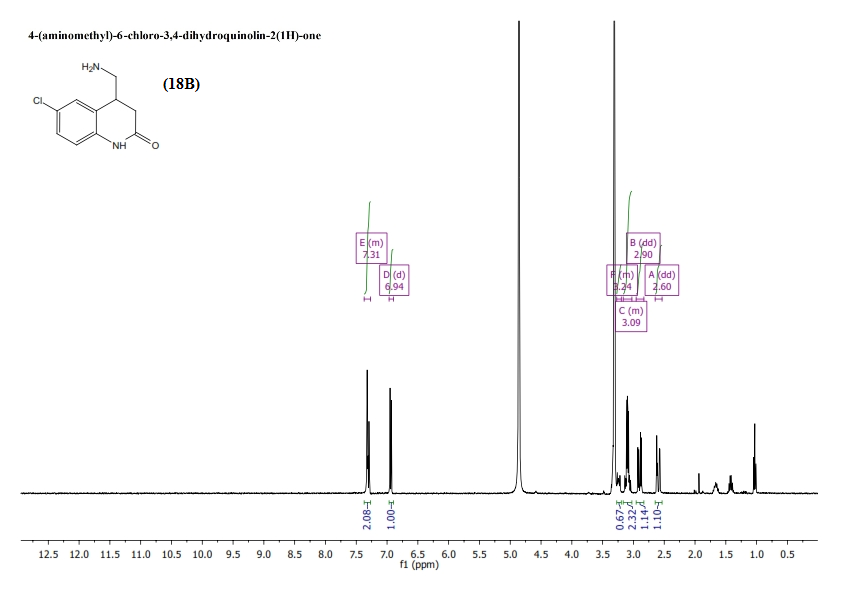


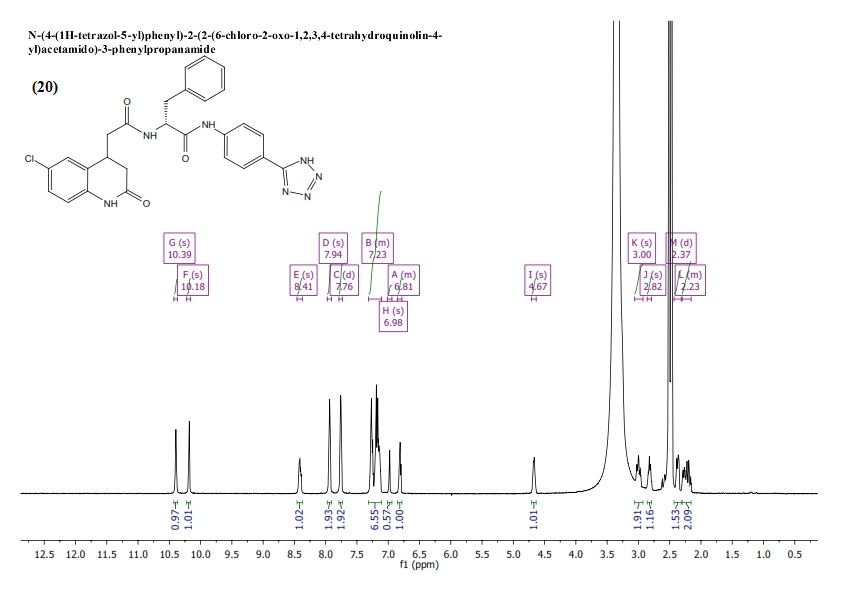


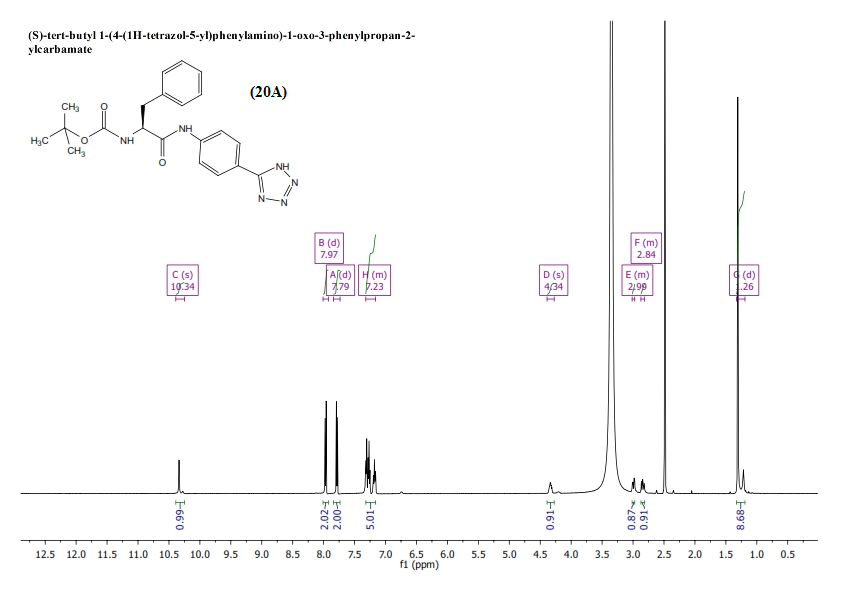


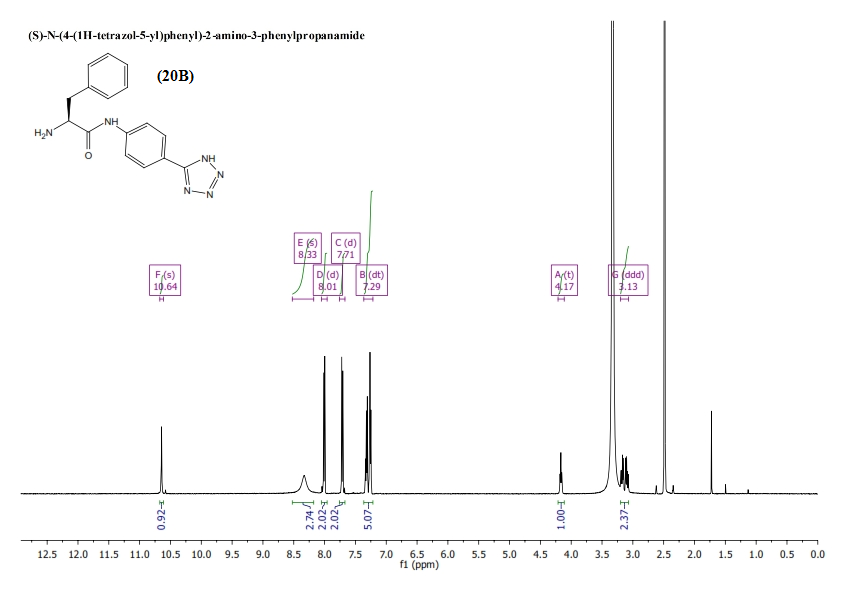


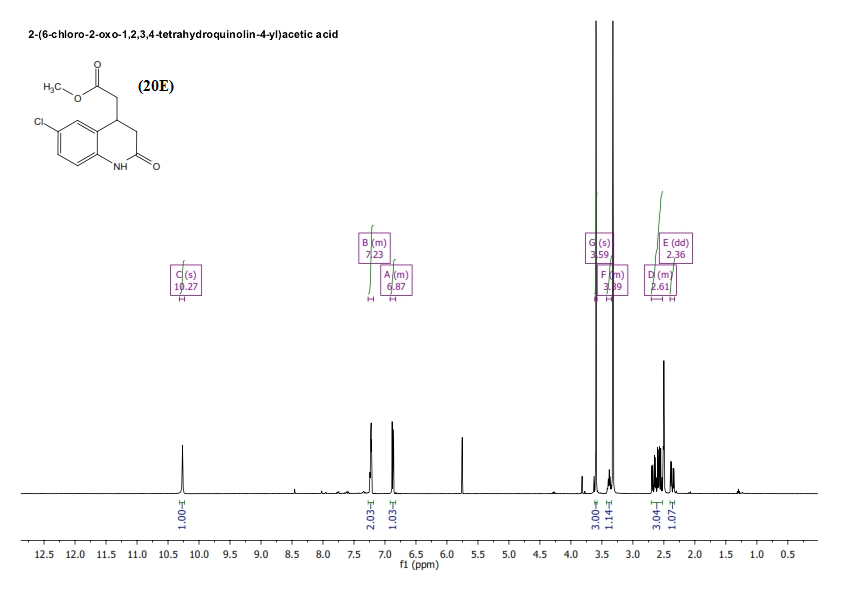


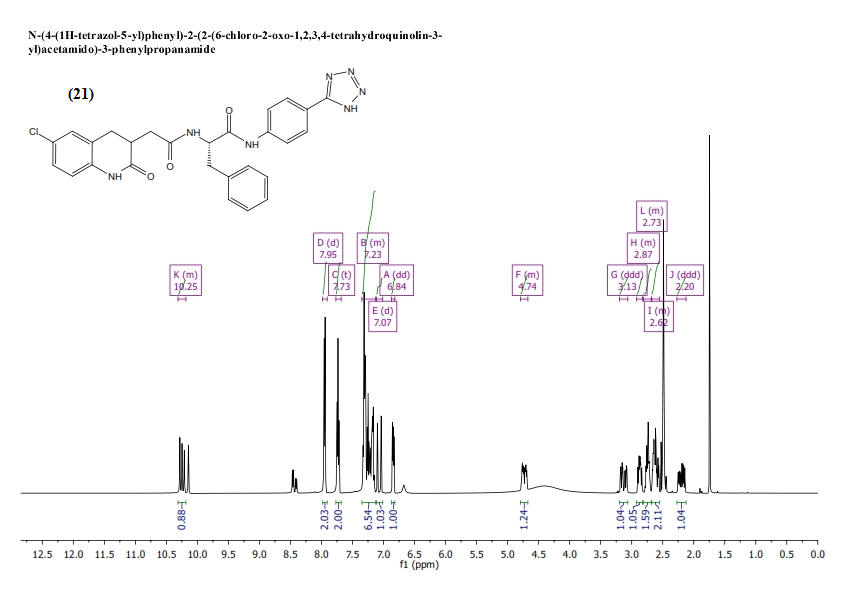


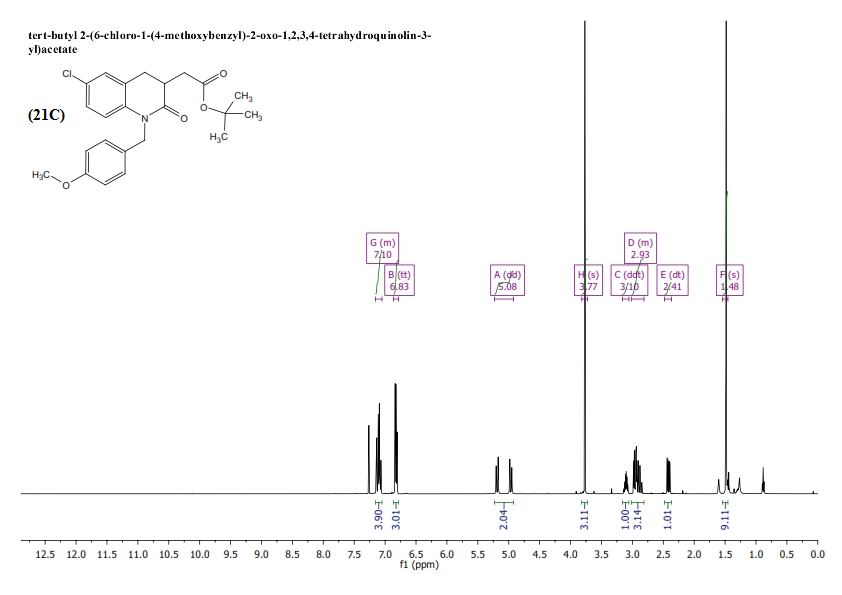


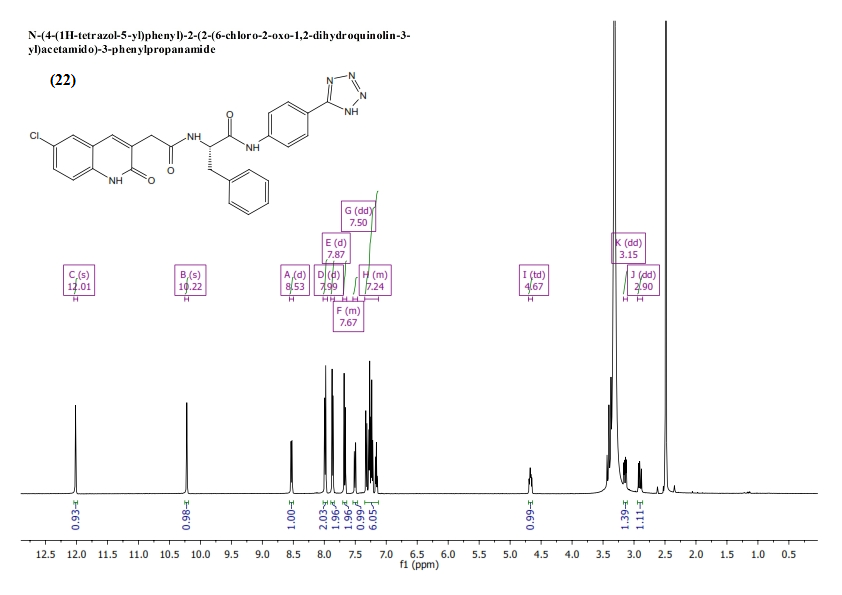


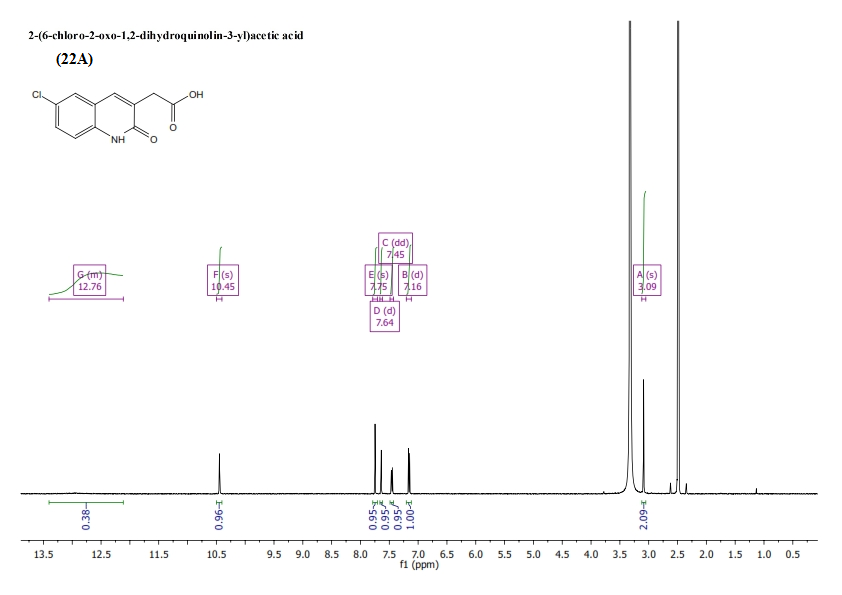


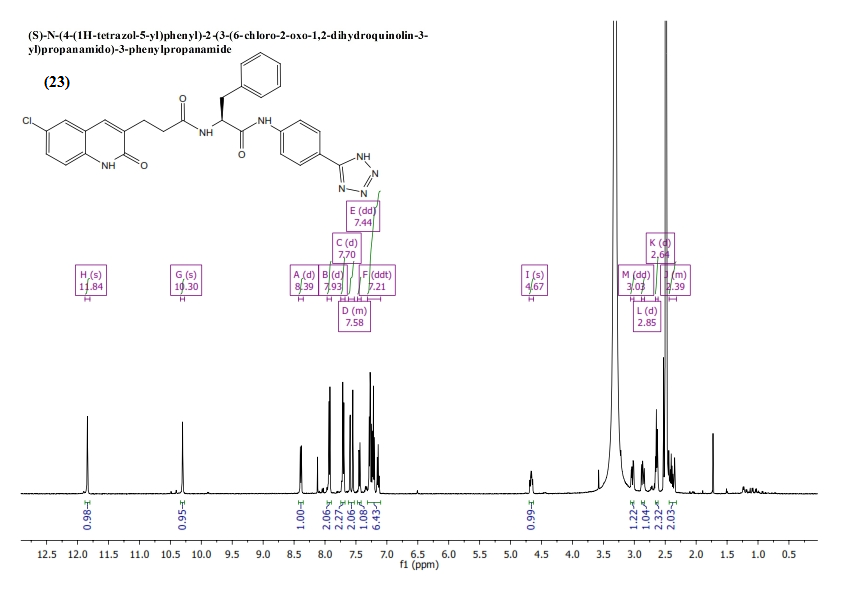


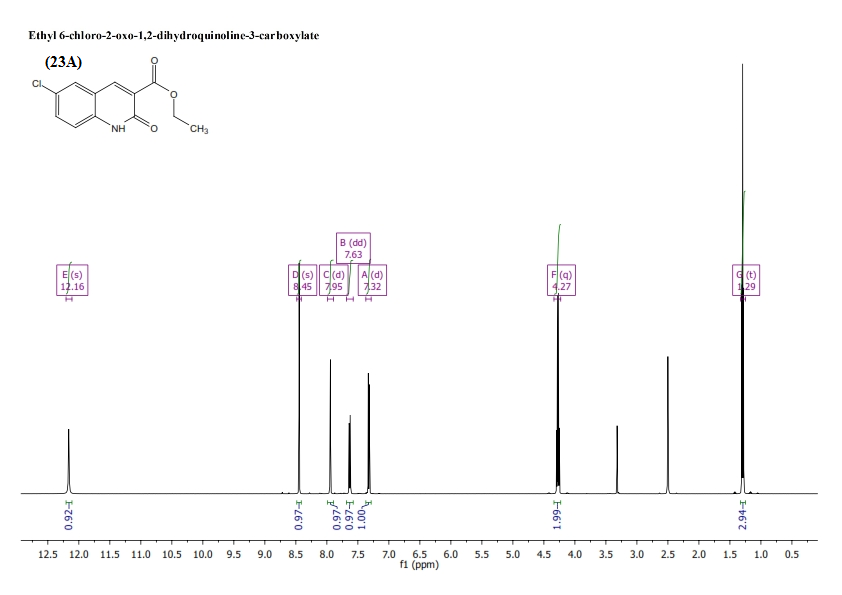


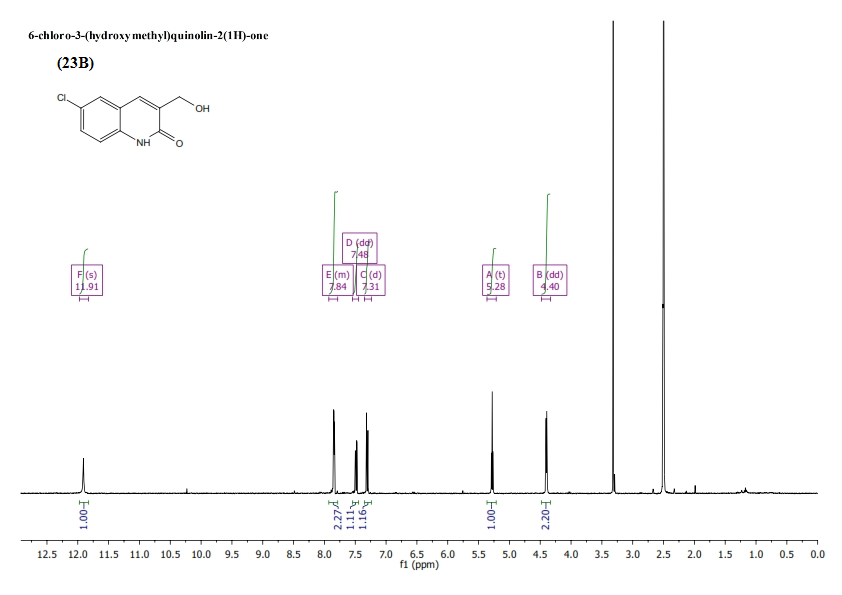


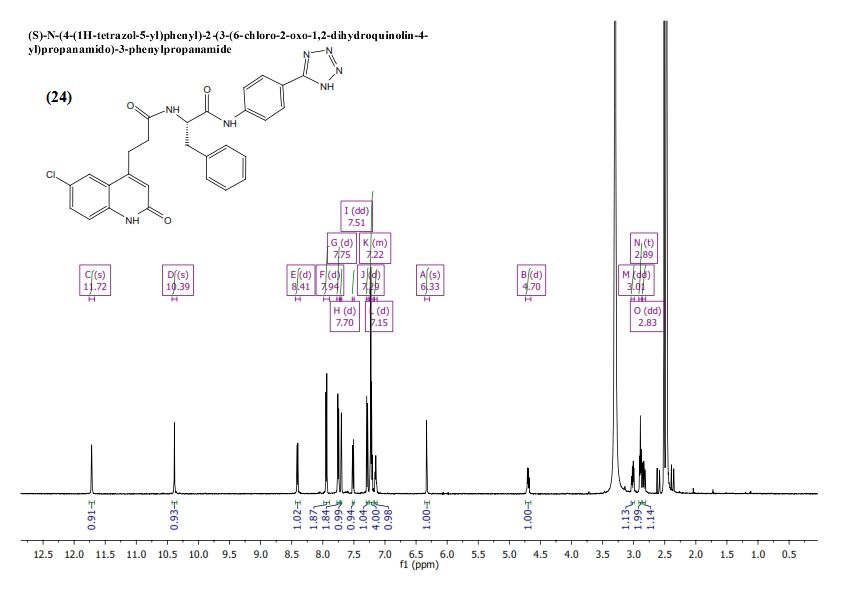


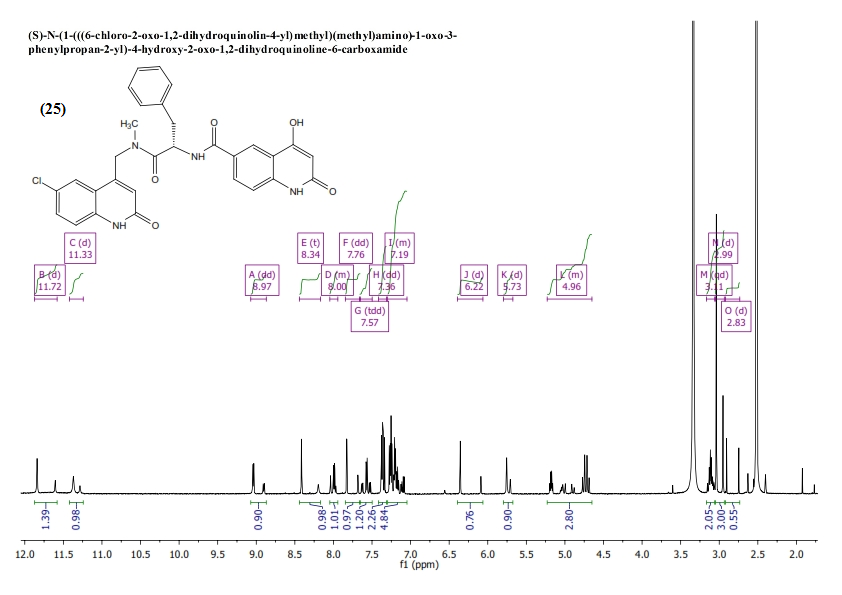


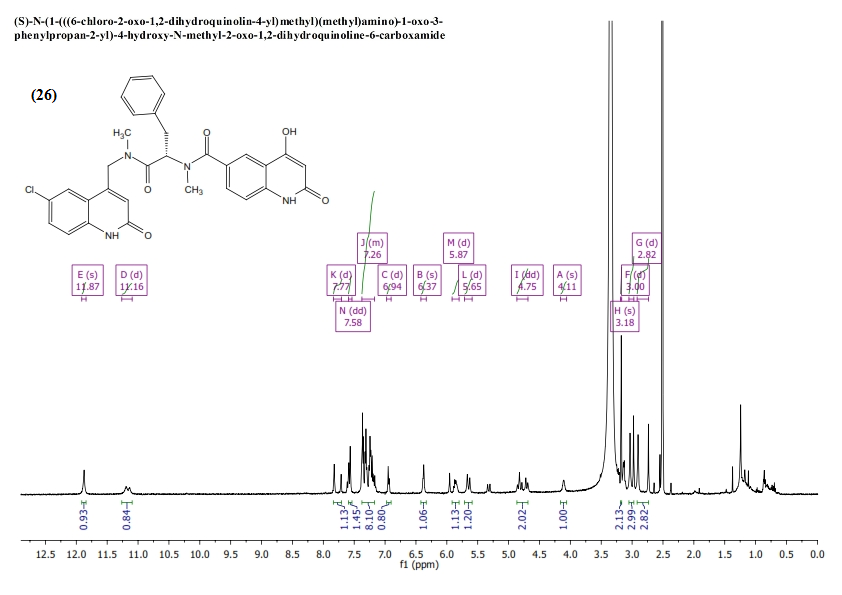


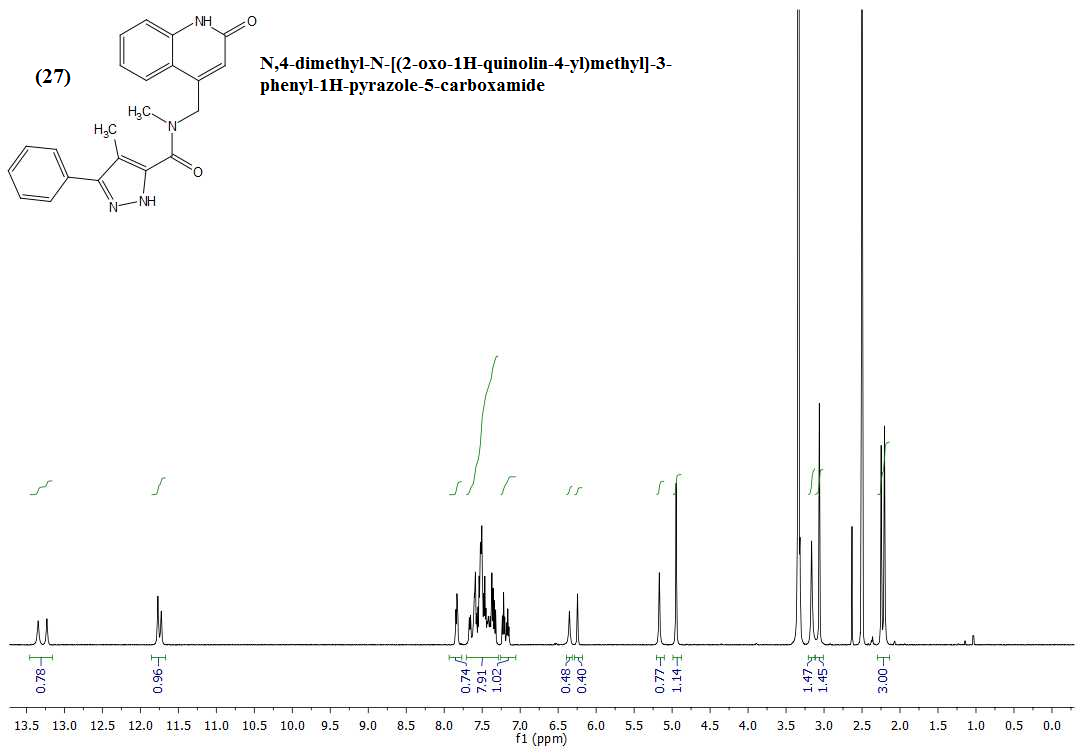


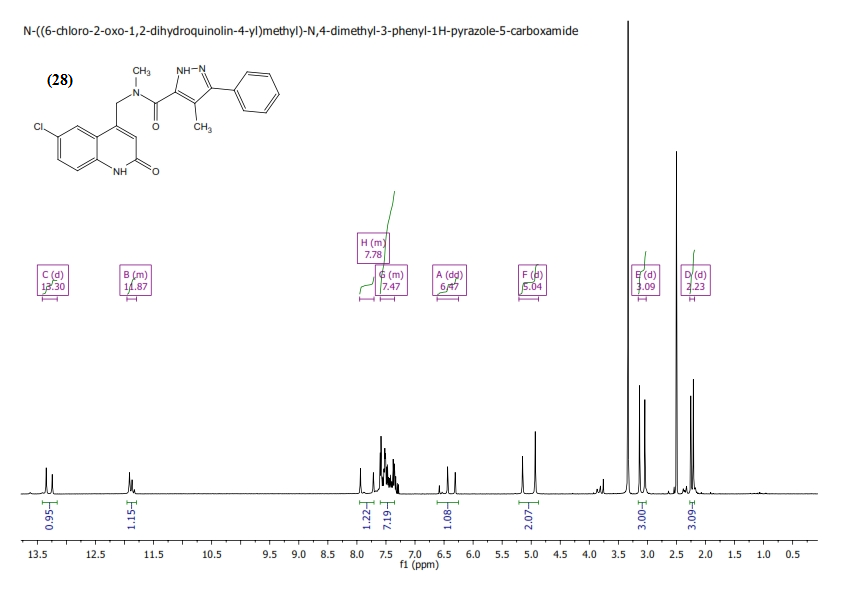

Supplement: S1 File — (DOCX) [file pone.0113705.s001.docx]
